# Investigating the mobilome in clinically important lineages of *Enterococcus faecium* and *Enterococcus faecalis*

**DOI:** 10.1186/s12864-015-1407-6

**Published:** 2015-04-10

**Authors:** Theresa Mikalsen, Torunn Pedersen, Rob Willems, Teresa M Coque, Guido Werner, Ewa Sadowy, Willem van Schaik, Lars Bogø Jensen, Arnfinn Sundsfjord, Kristin Hegstad

**Affiliations:** Research group for Host-microbe Interactions, Department of Medical Biology, Faculty of Health Science, UiT – The Arctic University of Norway, Tromsø, Norway; Norwegian National Advisory Unit on Detection of Antimicrobial Resistance, Department of Microbiology and Infection Control, University Hospital of North Norway, Tromsø, Norway; Department of Medical Microbiology, University Medical Center Utrecht, Utrecht, The Netherlands; Servicio de Microbiologia, Hospital Ramón y Cajal, Instituto Ramón y Cajal de Investigación Sanitaria (IRYCIS), Madrid, Spain; Centro de Investigación Biomédica en Red de Epidemiología y Salud Pública (CIBER-ESP), Madrid, Spain; Division of Nosocomial Pathogens and Antibiotic Resistance, Robert Koch Institute, Wernigerode Branch, Wernigerode, Germany; Department of Molecular Microbiology, National Medicines Institute, ul, Chełmska 30/34, 00-725 Warsaw, Poland; Division of Food Microbiologyt, National Food Institute, Danish Technical University, Copenhagen V, Denmark

**Keywords:** Hospital associated/clinical enterococcus, Horizontal gene transfer, Mobile genetic elements, Antibiotic resistance

## Abstract

**Background:**

The success of *Enterococcus faecium* and *E. faecalis* evolving as multi-resistant nosocomial pathogens is associated with their ability to acquire and share adaptive traits, including antimicrobial resistance genes encoded by mobile genetic elements (MGEs). Here, we investigate this mobilome in successful hospital associated genetic lineages, *E. faecium* sequence type (ST)17 (n=10) and ST78 (n=10), *E. faecalis* ST6 (n=10) and ST40 (n=10) by DNA microarray analyses.

**Results:**

The hybridization patterns of 272 representative targets including plasmid backbones (n=85), transposable elements (n=85), resistance determinants (n=67), prophages (n=29) and clustered regularly interspaced short palindromic repeats (CRISPR)-cas sequences (n=6) separated the strains according to species, and for *E. faecalis* also according to STs. RCR-, Rep_3-, RepA_N- and Inc18-family plasmids were highly prevalent and with the exception of Rep_3, evenly distributed between the species. There was a considerable difference in the replicon profile, with *rep*_*17/pRUM*_, *rep*_*2/pRE25*_, *rep*_*14/EFNP1*_ and *rep*_*20/pLG1*_ dominating in *E. faecium* and *rep*_*9/pCF10*_, *rep*_*2/pRE25*_ and *rep*_*7*_ in *E. faecalis* strains. We observed an overall high correlation between the presence and absence of genes coding for resistance towards antibiotics, metals, biocides and their corresponding MGEs as well as their phenotypic antimicrobial susceptibility pattern. Although most IS families were represented in both *E. faecalis* and *E. faecium*, specific IS elements within these families were distributed in only one species. The prevalence of IS*256*-, IS*3*-, IS*L3*-, IS*200*/IS*605*-, IS*110*-, IS*982*- and IS*4*-transposases was significantly higher in *E. faecium* than *E. faecalis*, and that of IS*110*-, IS*982*- and IS*1182*-transposases in *E. faecalis* ST6 compared to ST40. Notably, the transposases of IS*981*, IS*Efm1* and IS*1678* that have only been reported in few enterococcal isolates were well represented in the *E. faecium* strains. *E. faecalis* ST40 strains harboured possible functional CRISPR-Cas systems, and still resistance and prophage sequences were generally well represented.

**Conclusions:**

The targeted MGEs were highly prevalent among the selected STs, underlining their potential importance in the evolution of hospital-adapted lineages of enterococci. Although the propensity of inter-species horizontal gene transfer (HGT) must be emphasized, the considerable species-specificity of these MGEs indicates a separate vertical evolution of MGEs within each species, and for *E. faecalis* within each ST.

**Electronic supplementary material:**

The online version of this article (doi:10.1186/s12864-015-1407-6) contains supplementary material, which is available to authorized users.

## Background

The emergence of *E. faecalis* and *E. faecium* as leading hospital pathogens worldwide has been associated with their promiscuous nature in acquiring new genetic elements through HGT. HGT facilitates the adaptation of specific genetic lineages to the hospital environment by enabling acquisition of antimicrobial resistance, bacteriocin and virulence determinants that provide selective advantages and promote gastrointestinal colonization [1-8]. Molecular epidemiological studies using multi-locus sequence typing (MLST) and eBURST analysis identified a worldwide polyclonal cluster of hospital adapted *E. faecium* clones, termed at that time clonal complex (CC) 17, which contained sequence type (ST) 17 as well as its possible descendants; single locus variants ST16, ST78, ST63, ST64 and ST174 [9,10]. However, recent Bayesian-based population genetic modelling comparing whole genome sequences, suggests the existence of two clades of *E. faecium* strains (clade A and B), where clade A (A1) includes *E. faecium* associated with human infections from CC17 as opposed to clade B that contains strains of non-hospital human origin [6,11,12]. *E. faecalis,* in general, seem to be less origin- and/or host-restricted as dominant clones are shared between hospitals and the community although some CCs, including CC2, CC40 and CC87 show clear over-representation in hospital-associated infections [13].

Whole genome sequencing, comparative genome analysis and molecular epidemiological studies have provided important information about the content and distribution of MGEs in *E. faecalis* and *E. faecium* [11,14-21]. Plasmids, transposons, and prophages all contribute to the plasticity of enterococcal genomes [2,22] and clinical *E. faecium* strains have twice as many genes associated with MGEs as compared to non-clinical strains [16]. Sequence variability, presence of mosaic structures of plasmids combining modules from various origin and chimeric plasmids indicates a high genetic diversity of enterococcal plasmids [21,23-26]. Further, plasmid-mediated intraspecies chromosome-to-chromosome transfer of large DNA segments has been documented for both *E. faecalis* [3] and *E. faecium* [27]. In hospital-associated enterococci, plasmid stabilizing toxin-antitoxin (TA) systems, including Axe-Txe and ω-ε-ζ, are prevalent [26,28,29]. Notably, such TA-systems are increasingly considered as targets for development of new antimicrobial agents against multidrug resistant pathogens [30-34]*.* The impact of prophages on enterococcal diversity is less understood, but whole genome sequencing of *E. faecium* strains identified prophages as a prominent source of genome diversity [6,15,16].

Our epidemiological knowledge of MGEs in enterococci is limited and have mainly been based on the characterization of a restricted number of strains. The population structure of the examined strains have not been well characterized and their selection has been from a very broad range of origins and/or biased by certain characteristics such as specific antibiotic resistance mechanisms. Thus, we still have a considerable gap in our knowledge concerning the presence and relative distribution of known MGEs in different clinically relevant genetic lineages of enterococci and the potential role of these MGEs in the ecological dominance of enterococcal lineages in hospitals. In order to provide new insight in the complex mobile gene pool of *E. faecalis* and *E. faecium* (the mobilome [35]), we developed a DNA microarray with markers of enterococcal mobile genetic elements (including antimicrobial resistance genes) and CRISPR-cas elements identified so far. The arrays were hybridized with single strain genomic DNA of 40 enterococcal hospital associated strains. The mobilome profiles distinguished enterococci at species and subspecies level. The observed homogeneous hybridization pattern between *E. faecium* STs is in accordance with an evolutionary relatedness of ST17 and ST78 [6].

## Methods

### Bacterial strains

A total of 40 human *E. faecium* and *E. faecalis* strains representing four highly prevalent and clinical relevant STs; *E. faecium* (ST17, n = 10; ST78, n = 10) and *E. faecalis* (ST6, n = 10; ST40, n = 10), were selected. Relevant strain characteristics are given in Table 1. Briefly, the strains originated from nine European countries (Denmark, Germany, Italy, Norway, Poland, Portugal, Spain, Sweden, and The Netherlands) and were isolated between 1992 and 2009. With the exception of one fecal strain, all were clinical strains and some have been associated with hospital outbreaks. The fully sequenced *E. faecalis* V583 [36] was included as a control to monitor the hybridization quality.

**Table 1:** **Clinical enterococcal strains used for hybridization**

**No**	**ST/CC**	**Name**	**Source**	**Country**	**Year**	**Reference**
*E. faecium*						
1	17/17	U0229	Blood	NLD	1995	[37]
2	17/17	TUH2-18	Urine	NOR	1996	[38,39]
3	17/17	U0218	Blood	NLD	1997	[37]
4	17/17	E1463	Blood	ESP	1998	[40]
5	17/17	O2T878	Blood	SWE	2002	[41]
6	17/17	NIZP292/02	Wound	POL	2002	[42]
7	17/17	U0106	Blood	NLD	2004	[37]
8	17/17	VRE-10	Blood	DEN	2005	[43]
9	17/17	UW6900	Blood	GER	2006	[44,45]
10	17/17	VRE-84	VA	DEN	2008	[43]
11	78/17	E1644	Urine	GER	2002	[46]
12	78/17	U0262	Blood	NLD	2004	[37]
13	78/17	U0367	Blood	NLD	2004	[37]
14	78/17	E2603	Blood	NLD	2005	[37]
15	78/17	E4076	Blood	NLD	2006	[37]
16	78/17	UW6847	Blood	GER	2006	[45]
17	78/17	UW6880	Blood	GER	2006	[45,47]
18	78/17	HPH3	Urine	PRT	2007	[47]
19	78/17	VRE-106	Urine	DEN	2008	[43]
20	78/17	VRE0673	Feces	SWE	2008	[48]
*E. faecalis*						
21	6/2	229710	Urine	PRT	1992	[49]
22	6/2	2724	Blood	ITA	1993	[50]
23	6/2	217691	Blood	PRT	1996	[50,51]
24	6/2	E1828	Blood	ESP	2001	[52]
25	6/2	E3450	Blood	NLD	2006	[13]
26	6/2	UW7001	Blood	GER	2006	[13]
27	6/2	340/07	Blood	POL	2007	[13]
28	6/2	1665/07	Blood	DEN	2007	[13]
29	6/2	VRE-115	Blood	DEN	2008	[43]
30	6/2	3962/09	Blood	NLD	2009	[13]
31	40/40	435/96	Urine	POL	1996	[53,54]
32	40/40	UW1833	Urine	GER	1998	This study, [53,54]
33	40/40	7239/99	Urine	POL	1999	[53-55]
34	40/40	HC24	Blood	ESP	2001	[52-54]
35	40/40	457/04	Urine	POL	2004	[53-55]
36	40/40	UW5744	Urine	GER	2004	This study, [53,54]
37	40/40	UW6756	Urine	GER	2006	This study, [53,54]
38	40/40	UW7790	Feces	POL	2007	[53-55]
39	40/40	1638/07	Urine	DEN	2007	[13]
40	40/40	3992/09	Urine	NLD	2009	[13]
Control	6/2	V583	Blood	USA	1981	[36,56]

VA, ventricle aspirate; PF, peritoneal fluid; DEN, Denmark; ESP, Spain; GER, Germany; ITA, Italy; NDL, The Netherlands; NOR, Norway; POL, Poland; SWE, Sweden; USA, The United States of America.

### DNA isolation, hybridization and data acquisition

Single strain genomic DNA for microarray hybridization and PCR was extracted using E.Z.N.A Bacterial DNA kit (Omega Bio-Tek Inc., Norcross, GA) with the following modifications: cell walls were digested with 7 μg/μL lysozyme and 0.5 U/μL mutanolysin for 20 min at 30°C, and DNA was eluted with ddH_2_O. For hybridization, DNA was broken down to 100–600 bp fragments by 1 min sonication at 2 μm amplitude. Genomic DNA (2 μg) was fluorescent labeled using the Kreatech labeling kit with ULS-Cy5 according to the manufacturer’s instructions (Kreatech Biotechnologies, Amsterdam, The Netherlands), and degree of labeling (DoL) was calculated by measuring absorbance at 260 and 650 nm using a Nanodrop spectrophotometer. Hybridization of 4X2K CustomArrays were performed as described by the manufacturer (CustomArray Inc, Mukilteo, WA) at 52°C for 15 hours. Slides were subsequently scanned using an Axon GenePix ® 4000B scanner. Immediately after scanning, the slides were stripped for 90 min according to the manufacturer’s protocol. Control scans were performed each time to monitor possible background of the subsequent re-hybridization. The slides were stripped up to 6 times.

### Data analysis

*E. faecalis* V583 [36] was used as a technical replicate control on each fourplex array. Specifically, given that both the *E. faecalis* V583 genome and array probe sequences are known, number of false positive hybridizations could be monitored. A signal intensity cut-off could therefore be determined for each array, which reduces these hybridizations with more than 95%. The entire dataset was then normalized using quantile normalization. The overall correlation for all technical replicates included in study was 0.95, (see the correlation matrix for quantile normalized technical replicates in Additional file 1: Table S1). A hierarchical cluster diagram of hybridization data made in R calculated the distance by the method “complete”. Principal component analyses (PCA) and a cluster dendrogram were run on the resultant quantile normalized matrix (see Additional file 2: Figure S1A and B, respectively). The presence or absence of each target in each of the 40 clinical *E. faecium* ST17 (n = 10) and ST78 (n = 10) and *E. faecalis* ST6 (n = 10) and ST40 (n = 10) is presented in Additional file 3: Figure S2A-E. Each target is represented as a mean value of its 1–5 probes. Number of probes and probe sequences are listed in Additional file 4: Table S2.

### Probe design and final target list

The bioinformatics analysis and probe design of initially 405 targets sequences was done by CustomArray support (http://customarrays.com/index.htm) (last accessed December 10^th^, 2014). A total of 133 targets were excluded due to sequence similarity (>90%), sequence quality, and misleading annotation in GenBank. For the remaining 272 target sequences, 1–5 probes were designed. Suggested probes were blasted against a database built of the following enterocccal genome sequences: (*E. faecium* DO (GenBank acc. no. AAK03000000), *E. faecium strains* E1039 (NZ_ACOSO1000000), E1071 (NZ_ABQI00000000), E1162 (NZ_ABQJ00000000), E1636 (NZ_ABRY00000000), E1679 (NZ_ABSC00000000), E980 (NZ_ABQA00000000), and U0317 (NZ_ABSW01000000), and the *E. faecalis* strains OG1RF (CP002621), TX0104 (NZ_ACGL00000000), and TX1322 (NZ_ACGM00000000). Probes with most homologues (increased probability of false positive hybridizations) were excluded. The resulting 1250 probes were Tm-balanced by altering their length between 35 and 40 nucleotides. Number of final probes for each target and probes sequences are given in Additional file 4: Table S2.

The final target list included plasmid backbone genes (n = 85) encoding replication initiation proteins, genes associated with plasmid conjugation maintenance and plasmid addiction systems; transposable elements (n = 85) including conjugative transposons (integrases, excisionases and relaxases), Tn*3* family of transposons (transposases and resolvases), transposases associated to known IS elements, and other targets associated to transposable elements; genes encoding resistance (n = 67) towards relevant antibiotics (glycopeptides, aminoglycosides, β-lactamases, tetracyclines, macrolides/lincosamides/streptogramins B (MLS_B_ antibiotics), linezolid, chloramphenicol, and trimethoprim), biocides (disinfectants), and heavy metals; prophage sequences (n = 29) and CRISPR-*cas* sequences (n = 6). Thirty six plasmid replicon variants associated with enterococci were included in the analyses. Previously defined 20 *reps* [21,24] are designated by subscripted number representing replicon type and/or the name of the reference plasmid in Additional file 5: Figure S3A.

### PCR

The presence of important targets rejected during the probe design or targets representing newly described mobile genetic elements were examined by specific PCRs. They included *aac(6’)-aph(2”)-Ia* encoding the bi-functional aminoglycoside-modifying enzyme [57], the *vanA* and *vanB* clusters [38,58,59] as well as genes encoding the replication initiation proteins of plasmids pLG1 [29,60], pCF10 [24] and pIP501 (*repR*) [24]. To detect both *ω-ε-ζ* phylogenetic subgroups [17], the following primer pair specific for conserved regions were used: EU2f: 5′-GGCGGAAACGTAAAAGAAGTTATG-3′ and EU4r: 5′-TTCATTGACCGCCAATACTCATG-3′.

### Antimicrobial susceptibility testing

Antimicrobial susceptibility testing (AST) of the strains was performed to examine the validity of the microarray hybridization results for defining antimicrobial resistance determinants. AST towards ampicillin (2 μg), erythromycin (15 μg), gentamicin (30 μg), linezolid (10 μg), and tetracycline (30 μg) were done according to EUCAST (European Committee for Antimicrobial Susceptibility Testing) disk diffusion method [61]. Vancomycin susceptibility was examined using the brain heart infusion (BHI) agar screen method [62] using BHI supplemented with 6 mg/L vancomycin. The EUCAST clinical breakpoints [63] or clinical breakpoints defined by Clinical and Laboratory Standards Institute (CLSI) for antimicrobials not defined by the EUCAST were used for interpretation of AST.

### Statistical calculations

The statistical differences in hybridization profile of selected targets between either the two species (*E. faecium*/*E. faecalis*) or between STs within the two species (ST17/ST78 or ST6/ST40, respectively) were assessed by chi square (*χ*^2^) calculations without corrections and with 1 degree of freedom using the online calculator http://graphpad.com/quickcalcs/contingency1/ (last accessed December 10^th^, 2014). P-value <0.05 was considered significant.

## Results and discussion

Clinical relevant strains of European origin belonging to four predominant STs from the two decades of 1990 and 2000 were chosen for this study; *E. faecium* ST17 and ST78 which represent Bayesian Analysis of Population Structure (BAPS) groups 3–3 and 2–1 and *E. faecalis* ST6 and ST40 of CC2 and CC40, respectively. The DNA microarray was designed to cover mobile genetic elements (including antimicrobial resistance determinants) and CRISPR-cas elements associated with enterococci and known at that time.

### Overall hybridization profile and quality

A PCA was applied on the overall dataset and visualized in a two-dimensional plot (see Additional file 2: Figure S1A), also a dendrogram was created to visualize the result of a hierarchical clustering calculation of the mobilome (see Additional file 2: Figure S1B). The overall hybridization pattern showed *E. faecium* ST17 and ST78 clustered while the *E. faecalis* ST6 and ST40 formed two valid subgroups. As these data covers genes mainly involved in HGT and to a much less extent vertical descent, this does not describe the clonal relatedness of the strains analyzed. MLST-based data show that *E. faecium* ST17 and ST78 belong to two different BAPS groups (see above) suggesting that both STs are part of different hospital lineages and followed different evolutionary trajectories [64]. Although analyzing different strains than ours, the phylogenetic distance between *E. faecium* ST17 and ST78 is partly addressed through WGS by Lebreton *et al.* showing these strains grouped in one clade (clade A1) [6]. Within this clade, however, ST17 and ST78 cluster in different lineages, suggesting a certain degree of common evolutionary background yet diversification into separate genetic lineages. The highly-common mobilome found in our *E. faecium* ST17 and ST78 strains is most likely the result of sharing a common ecological niche (hospital) in which a common set of accessory genes is necessary to survive and thrive rather than evolutionary relatedness. Complete relatedness can be inferred by next generation sequencing (NGS), however, this is not the scope of our study. The DNA microarray technique was preferred over NGS as we searched only for absence or presence and not localization of specific targets. Also, reliable *de novo* whole genome assemblies from Illumina data addressing the mobile genome content is limited [65-67]. The dots representing every application of the control strain, *E. faecalis* V583 (ST6) form a tight cluster ensuring the comparable quality of the slides. The PCA shows that the selected targets in this array are both species- and ST-specific. Thus, the overall results of the PCA indicate that the dataset is technically accurate and biologically relevant. The accuracy of each repeat of the hybridization was also monitored by correlating the overall hybridizations of the technical replicate *E. faecium* V583 included in each fourplex array (Additional file 1: Table S1 and Materials & Methods). A correlation of 0.95 was obtained. As an additional quality control of the array, the presence and absence of antimicrobial resistance markers were compared with antimicrobial susceptibility testing and by selected consensus PCR analyses. This revealed that the results from the verifications were in agreement with the microarray hybridization results close to the expected 95% accuracy (data not shown).

### Species- and ST-specific patterns of mobile genetic elements

The detailed MGE gene profile of the 40 sample strains is given in Additional file 3: Figure S2A-E. The data are summarized for each of the four STs in Additional file 5: Figure S3A-E, significant differences (p < 0.05) between species and STs are indicated. The major species and ST-specific patterns are categorized and discussed below in relation to difference in content of plasmids, transposable elements, antimicrobial resistance determinants, prophages and CRISPR-*cas* modules.

#### Frequently detected plasmid associated gene targets

Plasmid-encoded genes involved in conjugation and mobilization as well as replication and maintenance were assessed by microarray hybridization analyses or, for a few targets, by PCR (Additional file 3: Figures S2A and Additional 5: Figure S3A). In order to sort out biological relevant patterns within the complexity caused by the modular structure of plasmids and the propensity of inter-plasmid recombination events, we defined the plasmid content in the four STs by presence of *rep* genes encoding replication initiator proteins and TA-encoding genes.

The average number of detected *rep*s per strain for *E. faecium* and *E. faecalis* (n = 6.75 and n = 5.45, respectively) were higher than previously reported for invasive *E. faecium* (3.2; [29]) and for clinical isolates of *E. faecalis* (2.54; [26]). This might reflect higher plasmid content in the selected STs, but may also be due to the higher number of *rep* targets included in the array. We suppose the factual number of plasmids to be lower than the identified *rep* genes since multi replicon plasmids and plasmid remnants in the core chromosome are known for many bacteria including enterococci [21,48].

As shown in Additional file 5: Figure S3A, *rep* targets belonging to the RCR, Rep_3, RepA_N, and Inc18 plasmid families (as defined by [68]) were well represented in both *E. faecium* (n = 34; n = 28; n = 36; and n = 21, respectively) and *E. faecalis* (n = 27; n = 6; n = 36; and n = 28, respectively). With the exception of the Rep_3 family (see below), there were no significant differences between the species or between the STs for the included strains. However, the composition of replicon types (as defined by [24]) within each plasmid family differed highly between *E. faecium* and *E. faecalis* and for some *rep* types between the STs as indicated. Our findings are in accordance with previous reports, although the composition and prevalence of the *rep*s appear to vary between strain collections of different origins [17,21,26,29].

The *rep*_*14*_ belonging to the RCR family was the most abundant replicon type in the strain collection with a significant (p < 0,0001) higher prevalence in *E. faecium* (n = 32) than in *E. faecalis* (n = 8). The *rep*_*14*_ replicons, which include the small mobilizable plasmids pEFNP1, pKQ10 and pRI1, have been considered specific for *E. faecium* [24,68-70]. Sequences originating from pEFNP1 and pKQ10 were prominent in a hospital clade specific *E. faecium* plasmid library [14]. Interestingly, the *rep* of pEFNP1 (target 19) was also found in half of the *E. faecalis* ST40 strains included in our study. Plasmids of *rep*_*7/pUSA02*_ type have been obtained from a broad range of hosts (*Enterococcus*, *Staphylococcus*, *Streptococcus* and *Bacillus*) and were represented in the array (target 431) by a *repD* of an *E. casseliflavus* plasmid [71]. Strikingly, the *rep*_*7*_ replicons were found in 12/20 of the *E. faecalis* strains, but only in two *E. faecium* strains (p < 0.0001). Taken together, the replicons of the RCR family appear to be abundant with a species-specific profile. Nevertheless, our observations support the notion that the examined STs of *E. faecium* and *E. faecalis* could share a pool of cryptic small plasmids through HGT. Their potential role of in the evolution of clinically important lineages of enterococci remains to be examined.

Rep_3 family includes several plasmids previously described in enterococci [72-75]. In our strain collection, a significant higher prevalence of Rep_3 family replicons were detected in *E. faecium* (n = 28) compared to *E. faecalis* (n = 6; p < 0.0001). In *E. faecium* represented by *rep*_*18*_ (n = 11), *rep*_*11*_ (n = 10) and *rep*_*pCIZ2*_ (n = 7), the two latter have a higher prevalence in ST78 compared to ST17 (p = 0.0377 and P = 0.0191, respectively). In *E. faecalis*, Rep_3 plasmids were represented by *rep*_*5/pN315*_ (n = 2), *rep*_*6/pS86*_ (n = 2) and *rep*_*11/pB82*_ (n = 2). This highly species-specific distribution of replicons indicate a narrow host range profile of the Rep_3 family. Their high abundance in the *E. faecium* strains compared to *E. faecalis,* might reflect a specific role of this plasmid family in the development of these important clinical lineages of *E. faecium*.

RepA_N is the most prevalent plasmid family, but with significant differences in *rep* types between the two species for the included strains. This is in accordance with the description of RepA_N replicons as narrow host range plasmids [76]. The *rep*_*9*_ variants of pheromone responsive plasmids [36,77-79] considered specific for *E. faecalis* were frequently detected in these strains but were absent in the *E. faecium* strains (p < 0.0001). Positive PCR results for *prgW* of pCF10 were observed in all *E. faecalis* ST6 strains. The *par* genes, encoding toxin RNAI and antitoxin RNAII originally found on the pheromone responsive plasmid pAD1 in *E. faecalis* [80], were found in 10/20 of the *E. faecalis* strains, were also absent in the *E. faecium* strains (p < 0.0001). On the other hand, the *rep*_*17/pRUM*_ and *rep*_*20/pLG1*_ replicons were present in 18/20 and 14/20 of the *E. faecium* strains, respectively, but absent in *E. faecalis* (p < 0.0001). *rep*_*17/pRUM*_ was the single most dominating replicon type among the selected *E. faecium* strains. This is consistent with previous reports, which also link the *axe-txe* TA-loci to this replicon type [17,21,41,48,81]. Both the *axe* and the *txe* targets (457 and 458) hybridized to 15/20 *E. faecium* strains, while none of the *E. faecalis* strains were positive (p < 0.0001). Moreover, co-hybridization with the *axe-*target was observed for 17/18 of the *rep*_*17/pRUM*_ positive and 12/14 *rep*_*20/pLG1*_ positive (data not shown) *E. faecium* strains. Also for *rep*_*20/pLG1*_, our findings are in agreement with previous investigations, where this *rep* was detected in 90% of *E. faecium* blood culture isolates [26] and linked to *axe-txe* on large conjugative plasmids encoding high-level gentamicin resistance [26] or glycopeptide resistance [19,50,60]. Our confirmatory findings support the importance of *rep*_*20/pLG1*_ megaplasmids in the evolution of hospital-associated lineages of *E. faecium*. Moreover, the contribution of *axe-txe* for the stable maintenance of clinical important RepA_N family plasmids in *E. faecium* is underlined.

The broad host range Inc18 family of conjugative plasmids is known to carry multiple antimicrobial resistance genes, including specific MLS_B_-resistance determinants hosted by streptococci, lactococci, staphylococci, and enterococci [82]. The pRE25 replicon type (*rep*_*2*_) had the same prevalence in *E. faecium* and *E. faecalis* (14/20), while *rep*_*1*_, which includes *rep*s from pAMβ1 and pIP501, was detected in 6/20 and 7/20 of the *E. faecium* and *E. faecalis* strains, respectively. No significant interspecies-differences in distribution of *rep* types between the included species or STs was detected, except for *rep*_*pHTβ*_ (n = 5), which had a higher presence in ST6 (n = 4; p = 0.0233), which underlines the broad host range of the Inc18 plasmid family and their propensity to support genetic exchange between the two species. However, Inc18 plasmids may have been established in enterococci at an early stage and essentially propagated by vertical descent.

The *ω-ε-ζ* TA-sequences included in the array originated from the Inc18 pVEF plasmids [83,84]. A discrepancy in presence between the three targeted TA-genes, which can be partly explained by allelic variation in the operon [17] or presence of the targets in other genetic contexts, was observed. However, to function as a plasmid stabilizing system, the presence of all three gene products is essential [85]. An additional PCR analysis using primers directed towards regions conserved for the two *ω-ε-ζ* phylogenetic subgroups [17] detected the TA-locus in a total of 14/20 of *E. faecium* and 17/20 of *E. faecalis*, where all ST40 strains were positive. In agreement with previous findings [17], co-hybridization to *rep*_*2/pRE25*_ was observed for *E. faecium* (11/14) and for *E. faecalis* ST6 (6/7), confirming the linkage of *ω-ε-ζ* to Inc18 plasmids in gram-positive pathogens and their contribution in stable persistence of clinically important plasmid encoded resistance traits (reviewed by [31]). However, *ω-ε-ζ* was also detected in the absence of *rep*_*2/pRE25*_ in both *E. faecium* (n = 3) and *E. faecalis* (n = 6) indicating linkage to other replicon types as well. Indeed, co-localization with *rep*_*9*_ belonging to the RepA_N family of pheromone responsive plasmids was reported for *E. faecalis* [26].

The *mazEF* originally observed on the *Escherichia coli* chromosome is one of the best-characterized TA-systems and have been identified in a large number of bacterial species [86,87]. For *Enterococcus*, the *E. coli mazEF* sequence has been reported to be ubiquitously present on *vanA*-plasmids in VRE strains including *E. faecium* and *E. faecalis* [28]. However, the *E. coli mazEF* could not be detected in an epidemiologically diverse collection of *E. faecium* strains [17] and BLAST search (http://www.ncbi.nlm.nih.gov/pubmed; last accessed December 10^th^ 2014) among draft genomes and plasmids did not show its presence in *Enterococcus*. In the current study the sequences from *E. faecalis* EnGen0297 strain HH22 (ACIX01000197.1) annotated as putative MazE and MazF were included (targets 464 and 465). All *E. faecalis* strains (n = 20) were positive for both targets, while the *mazEF* targets were not found in the *E. faecium* strains (p < 0.0001). BLAST search (last accessed December 10^h^ 2014) for the *mazEF* target DNA revealed a high prevalence in *E. faecalis* genomes where it appeared to be highly conserved, while no DNA homology was found in the *E. faecium* genomes, which is in accordance with our findings. However, conserved ORFs encoding putative MazF homologues were prevalent among the *E. faecium* draft genomes, indicating evolutional segregation of the two species. The functionality and linkage of these *mazEF* genes to the mobilome of *Enterococcus* need to be further investigated.

#### Widely distributed transposable elements

In addition to plasmids, three groups of transposons have been shown to facilitate flux of antimicrobial resistance determinants in enterococci [2]. Composite transposons transpose accessory DNA due to homologous flanking IS elements. Moreover, the Tn*3* family of transposons encode replicative transposition, and conjugative transposons support their own transfer between bacteria [2]. Our mobilome array included relevant members from all these three groups previously shown to be associated with enterococci. The IS elements belonged to families described by Clewell [68] and IS Finder (http://www-is.biotoul.fr/) (last accessed December 10^th^, 2014).

#### Different transposable elements in *E. faecalis* and *E. faecium*

A total of 12 IS families and 9 transposon groups were represented in our array. Additional file 3: Figure S2B shows the gene profile of all targets included in the microarray, which represents in many cases sequences of several transposases of the same IS family. Representative targets and their distribution are summarized in Additional file 5: Figure S3B, illustrating the predominance of transposable elements in *E. faecium* investigated clones (p < 0.0001). However, most of the selected targets represented transposable elements from this species and thus the relative overrepresentation of these targets among *E. faecium* is not unexpected. In particular, transposases of the IS families IS*256* (p = 0.0026), IS*3* (p < 0.0001), ISL*3* (p < 0.0001), IS*200*/IS*605* (p = 0.0058), IS*110* (p = 0.039), IS*982* (p < 0.0001), and IS*4 (*p = 0.0079*)* were significantly more abundant in *E. faecium* than *E. faecalis.* None of the included IS families were found at higher frequencies in *E. faecalis* strains than in the *E. faecium* strains although 10 of the 11 IS families represented in *E. faecium* were also found in *E. faecalis*. The presence of IS families in both *E. faecalis* and *E. faecium* specific strains imply that these elements are spread by HGT. However, particular IS elements are distributed in only one species suggesting that these IS elements have evolved over time within this species to become different from the other IS family members. Many variants of the Tn*916* family conjugative transposons have been reported (for review see [22]) and BLAST searches (last accessed December 10^th^ 2014) show that both the original Tn*916* and most of the variant transposons are reported in *E. faecalis*. For both reasons it is plausible that the conjugative transposon Tn*916*/Tn*1545* was observed more frequently in the investigated strains of *E. faecalis* (p < 0.0001) than *E. faecium* (Additional file 5: Figure S3B). Furthermore, Tn*916* have also been associated with pheromone responsive plasmids in *E. faecalis* [88,89], which accelerates their transfer among *E. faecalis*.

#### Abundant IS families and their possible association with resistance genes and transposons

The IS*256* family members (3 IS*256* variants as well as IS*1542*, IS*16*, IS*Ef1* and IS*1310*) are important components of many composite transposons conferring antimicrobial resistance such as Tn*5281* and Tn*4001* encoding HLGR [90], Tn*1547* (encoding vancomycin resistance [91,92]) and Tn*5384* (encoding erythromycin and gentamicin resistance [93]. Integrated IS*256*-like elements have also been observed in the Tn*3* family (Tn*1546*) [23,49,94]) and conjugative transposons (Tn*5382*) [39]. With the exception of IS*1542* and IS*1310*, the IS*256* family members in this study are found in the majority of the *E. faecium* strains (13/20 to 20/20) and in 7/20 to 20/20 *E. faecalis* strains. The presence of IS*Ef1*, IS*16* and IS*1542* (target 134, 98 and 102) [49], which is often observed among Tn*1546* variants does not correlate with the presence of this transposons (see Additional file 3: Figure S2B and Additional file 5: Figure S3B for details).

A study of *E. faecium* strains from different niches using comparative genomic hybridization (CGH) indicated an IS-driven diversification of hospital-adapted *E. faecium* strains [14]. Notably, IS*16* was suggested as the most hospital clade-specific marker in *E. faecium* (with 98% sensitivity and 100% specificity). This observation was further supported by detecting IS*16* in 155 of 160 invasive *E. faecium* strains, in contrast to only three positive for this elements among 100 *E. faecium* strains of human commensal, animal or food-associated origin [45]. All our clinical *E. faecium* strains were positive for IS*16*, while only three *E. faecalis* strains contained this sequence.

The IS*3* family was represented in this array by IS*Enfa3*, IS*1485,* IS*3-like*, and IS*981* transposases. IS*981,* which is mostly reported in lactococci, was found in 17/20 *E. faecium* strains and 12/20 *E. faecalis* strains. IS*1485,* which is widely spread in many enterococcal species [95], were found in all tested strains. The putative transposase of IS*Enfa3* was found in all *E. faecium*, while it was absent in *E. faecalis* (Additional file 3: Figure S2B and Additional file 5: Figure S3B). IS*Enfa3* was described as an epidemiological marker for Tn*5382* in South Korean *E. faecium* [96]. However, only three ST17 *E. faecium* strains were positive for Tn*5382*, thus IS*Enfa3*-like elements do not seem to be associated with Tn*5382* in our strains.

The IS*L3* family represented by transposases from IS*L3*, IS*L3*-like, IS*1251*, IS*Efa11*, IS*1476* and IS*1167* dominated in the *E. faecium* strains with the exception of two IS*L3*-like transposons (see Additional file 3: Figure S2B and Additional file 5: Figure S3B for details). The presence of IS*1251,* IS*Efa11* and IS*1476* transposase sequences which were reported integrated in Tn*1546-*like elements [97-101] did not correlate with presence of Tn*1546* (see Additional file 3: Figure S2B and Additional file 5: Figure S3B for details).

The abundance of the IS*200*/IS*605* family in *E. faecium* was due to IS*Efa4* and an *E. faecium* DO IS*605* transposase, while an IS*200* transposase from *E. faecalis* V583 was only found in *E. faecalis* (Additional file 3: Figure S2B and Additional file 5: Figure S3B). A putative IS*Enfa200* transposase described previously as being integrated in the *vanB* cluster of Tn*5382* [93] was not found in any of these strains. IS*Efa4* was found in a *vanS* gene of a *vanD* genotype VRE strain [102] as well as within a Tn*1546* element [103-105]. However, in our strains IS*Efa4* presence did not correlate with the presence of Tn*1546* (Additional file 5: Figure S3B).

The IS*110* family represented by three transposases was also significantly more abundant in *E. faecalis* ST6 than ST40 strains (p = 0.0027). This was due to the presence of IS*110* target 165 transposase in ST6 while IS*110* target 167 transposase dominated in *E. faecium* (Additional file 3: Figure S2B and Additional file 5: Figure S3B). The third transposase representing the putative IS*Enfa110* described previously integrated in Tn*5382* [92] was not found in any of these strains.

IS*Efm1* representing the IS*982* family has only been reported in a *vanD* operon of a vancomycin resistant *E. faecium* strain [106]. This IS element was present in all the *E. faecium* isolates and was also significantly more frequent in *E. faecalis* ST6 than ST40 strains (p = 0.025) (Additional file 3: Figure S2B and Additional file 5: Figure S3B).

The IS*4* transposase can be found in many enterococcal genomes, mostly in *E. faecium*, This is in accordance with our results, where it was found in 6/20 *E. faecium* strains only.

The IS*1182* family represented by IS*1182* transposase is more numerous in *E. faecalis* ST6 than ST40 strains (p = 0.0034) and is also present in 7/20 *E. faecium* strains (Additional file 3: Figure S2B and Additional file 5: Figure S3B). This IS element delimits the streptococcal composite transposon Tn*5405* carrying resistance to aminoglycoside-streptothricin (*aadE-sat4-aphA-3*) [107]. In multiple-resistant *E. faecium* Tn*5405*-variants were also found genetically linked to the MLS_B_ determinant *ermB* and in one case also to the glycopeptide resistance cluster *vanA* [108]. In concordance with this, IS*1182* was in this study observed to be present only in strains containing *sat4, aphA-3/aph(3)-IIIa* and *ermB/erm2* (targets 247, 381 and 298, respectively). However, these resistance genes were observed also in strains lacking IS*1182 (*Additional file 3: Figure S2B).

Due to IS*1678,* the IS*1380* family also represented by an IS*Ecp1*-like transposase, was statistically more prevalent in ST78 than ST17 *E. faecium* (p < 0.0001) (Additional file 3: Figure S2B and Additional file 5: Figure S3B). IS*1678* has previously only been reported twice integrated in Tn*1546* [109] and close to Tn*1546* on a *rep*_*17/pRUM*_ replicon [110] in *E. faecium*. These IS*1678* elements were, except for two strains, not associated with Tn*1546* containing strains and detected in only 7/18 *E. faecium* strains containing the *rep*_*17/pRUM*_ replicon in our study.

#### Predominant transposable elements encoding clinically relevant antimicrobial resistance

To visualize the impact of resistance markers on the overall dataset, a second PCA excluding data from the targets encoding resistance markers was built (data not shown). This did not alter the overall plot substantially suggesting the resistance markers to be generally genetically linked to the other included MGEs.

The Tn*916*/Tn*1545* family of conjugative transposon (CTn) is widespread, but primarily observed in the Firmicutes such as enterococci. This CTn-family seems to have a particular ability to acquire accessory genes including resistance determinants, to cause genome rearrangements and to mobilize other replicons. The originally described Tn*916* carries the tetracycline resistance gene *tetM*. Tn*1545* is homologous to Tn*916*, but contains additionally *ermB* (encoding resistance to MLS_B_ antibiotics) and *aphA-3* (encoding resistance to kanamycin) cassettes (reviewed in [111,112]). In our array the Tn*916*/Tn*1545* family was represented by an integrase (target 199), an excisionase (target 213) and a conjugative transfer protein gene (target 75) as well as the tetracyclin resistance determinant *tetM* (target 243). The integrase, excisionase and conjugative transfer protein genes were significantly more abundant in *E. faecalis* than *E. faecium* (p < 0.0001) as well as in ST17 than ST78 (p = 0.02) of *E. faecium* (see Additional file 3: Figure S2B and Additional file 5: Figure S3B for details). All *E. faecalis* strains hybridized to the Tn*916* targets while the Tn*916* specific integrase and excisionase were detected in only 2/10 ST17 and were absent in ST78 *E. faecium.* The *esp*-containing ICE*Efm1* has a conjugation module similar to Tn*916* [113]. However, the conjugative transfer protein from this conjugation module shows only about 80% identity at protein level to the Tn*916* prototype protein (ORF16 target 75) and thus the DNA identity level was far too low to detect ICE*Efm1* through the Tn*916* conjugation protein in our array.

The Tn*3* transposon family transposon Tn*1546* is genetically linked to the *vanA* resistance cluster encoding high-level glycopeptide resistance [114]. In our array target 146 represents the transposase [83,115], target 216 the associated resolvase [83,114] and target 237 the prototypical D-ala:D-lac ligase (*vanA*) of Tn*1546* [115], which were present in the same strains except for *vanA* which was found in an additional ST17 strain (Additional file 3: Figure S2B and Additional file 5: Figure S3B). PCR (*vanA*) and phenotypical analysis (vancomycin) confirmed the hybridization patterns (Additional file 5: Figure S3C and data not shown).

The conjugative transposon Tn*5382*/Tn*1549* is closely linked to the *vanB2* gene cluster [92,116,117]. This transposon was represented in our array by probes targeting the genes of the excisionase of Tn*1549* (target 454), the integrase (target 452), the relaxase (target 39), the TrsE-like protein (target 82), and the D-alanine:D-lactate ligase (*vanB*; target 238) (Additional file 3: Figure S2B and C, Additional file 5: Figure S3B and C). A moderate number of the *E. faecium* and *E. faecalis* strains hybridized to these targets. PCR analyses and antimicrobial susceptibility testing confirmed that *vanB* was present only in isolates containing all Tn*5382*/Tn*1549*-related targets.

#### Broad content of genes encoding antimicrobial resistance

The enterococcal STs in this study were selected due to their relative dominance in clinically relevant strains. Although the presence of resistance traits was not a selection criterion, the collection is biased towards antimicrobial resistance and an overrepresentation of these markers was expected. The phenotypic expression of resistance, examined by standardized AST, was used to validate the hybridization results for defined antimicrobial resistance determinants. Moreover, the presence of *vanA*, *vanB* and *aac(6’)-aph(2”)-Ia* (determining high-level gentamicin resistance) was also examined by PCR.

The hybridization results of screening for the antimicrobial resistance determinants are shown in Additional file 3: Figure S2C and summarized in Additional file 5: Figure S3C. The results are categorized according to genes encoding resistance towards glycopeptides, aminoglycosides, β-lactams, tetracyclines, MLS_B_, oxazolidinone, chloramphenicol, trimethoprim, biocides and heavy metals. As shown by PCA and cluster dendrogram (see Additional file 2: Figure S1A and B) a separation in gene content between the two species and the two STs of *E. faecalis* can be seen. Defined resistance markers were selected for phenotypic verifications, and we observed an overall high correlation as outlined below.

Vancomycin resistance determinants were represented by six targets (237 for *vanA*; 238 for *vanB*; 280 for *vanE*; 281 for *vanG*; 284 for *vanG*_*2*_; 313 for *vanL*). A total of 3/10 ST17 and ST78 *E. faecium* and ST6 *E. faecalis* strains were positive for the *vanA* target (Additional file 5: Figure S3C). The *vanB* target scored positive in 2/10 ST17 and ST78 *E. faecium* strains. The *vanG* and *vanL* targets scored positive only in one and two *E. faecalis* ST40 strains, respectively (Additional file 5: Figure S3C). Phenotypic testing was in agreement with the hybridization results except for the *vanG* and *vanL* target positive ST40 strains (data not shown). A total of 16/40 strains expressed vancomycin resistance of which ten and six strains were confirmed as positive for *vanA* or *vanB* by PCR, respectively. As compared to *vanA*-, *vanB*- and *vanG*-specific PCRs, the microarray hybridization results yielded two false negative results (one *vanA* and one *vanB E. faecium*) and two potential false positive results (one *vanA E. faecium* and one *vanG E. faecalis*). Phenotypically silent *vanA*-determinants have previously been reported [118-120].

The presence of aminoglycoside resistance determinants showed species and ST-specific patterns. The *E. faecium* species-specific target *aac(6”)-Ii* (target 254) encoding an aminoglycoside 6′-N-acetyltransferase that mediates resistance towards many aminoglycosides except gentamicin was present in all *E. faecium* strains (Additional file 5: Figure S3C). Target 247, 383 and 381 representing *sat4, aadE* and *aph(3)-IIIa*, respectively, were present in at least 10/20 strains of both *E. faecium* and *E. faecalis* (Additional file 5: Figure S3C). Other targets representing aminoglycoside resistance determinants were only sporadically present. The gene cluster *aadE-sat4-aphA-3* has been linked to composite transposon Tn*5405* delimited by IS*1182* [107]. All three resistance genes were found in 21/40 of the isolates (Additional file 5: Figure S3C) although *aadE* was additionally present in four, *sat4* in one and *aphA-3* in three isolates. IS*1182* was present in 13/21 of the isolates that contained all three resistance determinants.

Due to sequence quality (lengths of nucleotides with adequate Tm) and target homology problems, the target encoding the bifunctional enzyme AAC(6’)-Ie-APH(2”)-Ia conferring HLGR was not represented on the array. The presence of the *aac(6’)-Ie-aph(2”)-Ia* gene was therefore analyzed by PCR and yielded 11/20 and 9/20 of *E. faecium* and *E. faecalis* positive for this gene*,* respectively. Notably, 7/10 *E. faecalis* ST6 while only two *E. faecalis* ST40 were positive for *aac(6’)-Ie-aph(2”)-Ia*. A strong correlation between the presence of *aac(6’)-Ie-aph(2”)-Ia* gene and HLGR was observed (data not shown). This observation is consistent with a recent study of European *E. faecalis* strains that showed a high prevalence of HLGR in CC2 (96%) in contrast to CC40 (5%) strains [13].

Determinants of tetracycline resistance were represented by five targets (243 for *tetM*; 242 for *tetL*; 262 for *tetK; 385* for *tetO; 386* for *tetS*). Using the CLSI clinical breakpoints, a total of 29/40 strains expressed tetracycline resistance (data not shown). Hybridization analyses revealed that all tetracycline resistant strains harbored one; two or three *tet*-resistance determinants (see Additional file 3: Figure S2C and Additional file 5: Figure S3C for details). Only 2/11 tetracycline susceptible strains carried *tet*-resistance determinants (*tetM* and *tetL*), of which both were *E. faecium* ST78 strains. Interestingly, all *E. faecalis* strains expressed tetracycline resistance and were shown to carry *tetM* (n = 20), *tetL* (n = 4) or *tetK* (n = 4). Thus, there is a strong correlation between the presence of Tn*916*/Tn*1545* targets and *tetM* resistance determinants in *E. faecalis*. This observation is in contrast with the findings obtained for *E. faecium* where eight *tetM* positive strains were negative for Tn*916 xis* and *int* targets. This is even more pronounced than the data from Polish VRE *E. faecium* isolates where 59 of 72 isolates containing *tetM* also were positive for the Tn*916* integrase gene [42]. These observations could be explained by the location of *tetM* in other conjugative transposons such as Tn*5801* or Tn*6000*, frequently found in *E. faecium* [68]; (Leon-Sampedro, personal communication).

Erythromycin resistance determinants were represented by eight targets (260 for *ermA*; 298 for *ermB*; 398 for *ermF*; 399 for *ermG*; 401 for *ermQ*; 396 for *ermT*; 402 for *ermTR*; 309 for *mef*). A total of 31/40 and 8/40 strains showed resistance or intermediate susceptibility to erythromycin using the CLSI clinical breakpoints for interpretation (data not shown). Hybridization analyses revealed that all erythromycin resistant strains harbored one of these erythromycin resistance determinants (*ermB* n = 29; *ermT* n = 1; *mef* n = 1). One susceptible strain was positive for *ermB*. Determinants *ermA*, *ermF*, *ermG*, and *ermQ* were not detected. Four out of 8 intermediate strains did not contain any erythromycin resistance determinants, while the other four were positive for *ermB* (n = 1), *mef* (n = 2) or *mef* + *ermT* (n = 1). One strain was susceptible and also negative for all erythromycin resistance determinants. Acquired resistance towards MLS_B_ in enterococci is most often due to the presence of the *ermB* gene [121-126]. *ermB* was identified in 17/20 *E. faecium* strains as well as 10/10 and 3/10 *E. faecalis* ST6 and ST40 strains, respectively (Additional file 5: Figure S3C). Although *ermB* was originally described as part of Tn*917* in *E. faecalis* [127] we do not find a correlation between presence of *ermB* and Tn*917* targets that were found only in 1 (resolvase target 229) and 2 (transposase target 145) of the 10 ST6 *E. faecalis* strains. However, *ermB* presence have been reported on different plasmids [114,128-131] suggesting that *ermB* is transferred by plasmids among enterococci.

Several chloramphenicol acetyltransferases of different origins were represented on the array (targets 387–393; Additional file 4: Table S2). Target 390 scored positive in 5/20 *E. faecalis* and 3/20 *E. faecium* strains, while target 387 scored positive in 9/20 *E. faecalis* strains (Additional file 5: Figure S3C). Antimicrobial susceptibility testing towards chloramphenicol was not performed. Finally, an oxazolidinone target (target 394) representing a gene encoding rRNA methylase Cfr from a *Staphylococcus warneri* plasmid was present on the array and one *E. faecalis* strain ST40 scored positive for this target. However, the strain was susceptible to linezolid, also PCR analysis did not detect the *cfr* gene (data not shown) indicating unspecific hybridization.

When summarizing the relative presence of the different antimicrobial resistance determinants (n = 67) *E. faecalis* ST40 strains had the highest positive score (average of 10.5% positive targets per strain) as compared to *E. faecium* ST17, *E. faecium* ST78 and *E. faecalis* ST6 with an average of 6.6%, 6.9%, and 7.4%, respectively (Additional file 3: Figure S2C). The *tetM, tetS* and *msrC* genes, responsible for tetracycline and erythromycin resistance, respectively, contributed largely to this difference (Additional file 5: Figure S3C).

#### High prevalence of genetic determinants for cadmium and QAC resistance in *E. faecalis* ST40

While the selected targets representing resistance traits towards copper (406 for *tcrB*), mercury (407 for *merA* and 408 for *merB*), cadmium (409 for *cadA*), and quaternary ammonium compounds (QAC) (410 for *qacA*, 412 for *smr*/*qacD*, 413 for *qacG*, 414 for *qacH* and 415 for *qacJ*) seem uncommon in the *E. faecium* strain collection, a number of *E. faecalis* and in particular ST40 strains hybridized to them (see Additional file 3: Figure S2C and Additional file 5: Figure S3C for details). Hybridization occurred mainly in the strains most recently recovered (2006–2009, Additional file 3: Figure S2C). The cadmium and QAC resistance determinants were found in the same 6/10 *E. faecalis* ST40 strains as well as in 1/10 ST6 strain but the possible genetic linkage of these resistance determinants will have to be investigated by other methods.

Biocide (e.g., alcohol, aldehyde, biguanides (chlorhexidine), quartenary ammonium compounds, zinc, phenolic compounds) resistance has been rarely detected among enterococcal strains, despite its frequent detection in clinical isolates of other Firmicutes, e.g. the emergence of QAC resistance in Methicillin Resistant *Staphylococcus aureus* due to acquisition of plasmid-borne *qac* genes [132,133]. Further, evidence suggests that biocide and antibiotic resistance determinants are linked within the same genetic unit raising the question if the use of biocides can contribute to the selection of antimicrobial resistance [134-139]. However, the previous observed co-localization of the copper resistance determinant with *ermB* [136,137] or *vanA* [136] in enterococci from animals was not observed in this strain collection representing four highly prevalent, clinical relevant ST types.

#### Delimited presence of prophage determinants

Although transduction of virulence and resistance genes were recently demonstrated in enterococci [139,140], the extent and importance of HGT due to bacteriophages in enterococci is not fully understood. Prophage sequences are commonly found in CC2 strains [19] including the fully sequenced *E. faecalis* V583 which harbors seven prophage-like elements [36,141], six of which are proven biologically active [142]. Based on the current knowledge on these sequences at the time the array was printed, five of them (pp1, pp3, pp4, pp6, and pp7) were represented in our assay, Additional file 3: Figure S2D and Additional file 5: Figure S3D. With the exception of one strain from each species, the *E. faecalis* V583 prophage sequences were present in all *E. faecalis* test strains, and in none of the *E. faecium* strains. Prophage7 (pp7) is able to excise from the chromosome by the aid of pp1 in a manner that resemble chromosomal islands, and have therefore been renamed *E. faecalis* chromosomal island of V583 (EfcIV583) [132]. This target was only found in two *E. faecalis* ST6 strains. Eight different *E. faecium* prophage sequences (24 target sequences of various origins both non-hospital and clinical strains) were represented on the array [15]. With one exception (prophage sequences from *E. faecium* E980, targets 315–317), these sequences showed species specificity toward *E. faecium*. Prophage sequences from the clinical *E. faecium* ST78 strain U0317 (targets 336–341) dominated the *E. faecium* strain collection and was especially well distributed in the ST78 strains.

Recent studies have identified additional phage sequences to those included in this array (reviewed in [143]) and one should account a possible different outcome if these were included. However, prophage sequences are commonly found in our collection of *E. faecium* and *E. faecalis* strains and are in general species-specific, suggesting HGT events as well as vertical transmission of these prophage sequences.

#### *Presence of CRISPR-Cas system only in* E. faecalis *ST40 strains*

The Clustered Regularly Interspaced Short Palindromic Repeat (CRISPR)/CRISPR associated (Cas) system provides immunity against bacteriophage infections and mobile genetic elements in prokaryotes by genetic memory and (RNA guided) DNA interference (reviewed in [144,145]). The CRISPR-Cas system was represented in our array by four *E. faecalis* OG1RF CRISPR associated proteins (encoded by *cas*- and *csn* genes; targets 474–477) and two conserved *CRISPR* repeat sequences; one from *E. faecium* (target 478) and *E. faecalis* OG1RF (targets 479). None of the 20 *E. faecium* strains was positive for any of the CRISPR-*cas* targets (Additional file 3: Figures S2E and Additional file 5: Figure S3E). This is in agreement with previous reports on hospital adapted *E. faecium* lineages with a broad content of antibiotic resistance traits [146] and prophage sequences [15]. This inverse correlation was also expected for the *E. faecalis* strains [146,147]; however 90% of the ST40 strains contained both *CRISPR* and *cas* sequences, and thus likely a functional system. All these strains also contained multiple acquired antibiotic resistance genes challenging the hypothesis of Palmer and Gilmore [146] that multidrug resistant enterococci generally lack CRISPR-*cas* genes. A similar observation was found in 15 multidrug resistant whole genome sequenced *E. faecalis* ST40 isolates showing supposedly complete CRISPR-Cas systems [148]. It is, however, difficult to elucidate any possible targets for this CRISPR-Cas system without sequencing the CRISPR spacer region to search for specific target sequences.

## Conclusions

Our microarray-based study revealed an overall high prevalence and a species-specific distribution of mobile genetic elements in the selected clinically relevant *E. faecium* and *E. faecalis*. For the *E. faecalis* strains, the presence or absence of these targets also separated the two STs. The separation of *E. faecalis* ST6 and ST40 was especially evident for the biocide genes, *E. faecalis* prophage sequences and *cas* genes.

Although the presence of broad host range conjugative Inc18 family replicons substantiates the possibility of inter-species horizontal gene transfer, both species were dominated by narrow host range replicons. The high species-specific prevalence of the RCR family of small cryptic plasmids, the Rep_3 family and the RepA_N family including pLG1 megaplasmids and pheromone responsive plasmids imply an evolutionary significant role in the development of the hospital associated STs hosting them.

An association between the ω-ε-ζ plasmid addiction system and Inc18 plasmids was verified for *E. faecium*, but the TA locus was also detected in the absence of this plasmid family in both species. The contribution of TA-systems for stable maintenance of important virulence and resistance carrying plasmids in *Enterococcus* is also underlined by the high prevalence of the *axe-txe* locus and its association to the RepA_N plasmids in the *E. faecium* strains.

Specific IS elements are distributed in only one of the species implying that these have evolved vertically. However, the presence of most IS families in both specific strains *E. faecalis* and *E. faecium* suggest that these elements are also spread by HGT. Within *E. faecium*, Tn*916*/Tn*1545* and IS*1380* were specific for ST17 and ST78, respectively. All 20 *E. faecalis* strains contained sequences of Tn*916*/Tn*1545*, while IS families of IS*110*, IS*982* and IS*1182* showed a preference towards ST6. The relative lack of Tn*916* family conjugative transposons in *E. faecium* compared to *E. faecalis* in our strains fits with most reports of this transposon family in *E. faecalis*. There was also a strong correlation between the presence of Tn*916* targets and *tetM* in *E. faecalis* that was not found in the *E. faecium* strains. The presence of Tn*917* resolvase and transposase in only one and two of the 30 strains that harbored *ermB* correlates well with few descriptions of Tn*917* containing enterococci in the literature although *ermB* was originally described as part of this transposon in *E. faecalis*.

The overall findings confirm and unfold our previous knowledge on the extensive reservoir of mobile genetic elements and underline their importance in evolutionary development of clinical relevant lineages of enterococci. Moreover, the detected MGE pattern support a significant species- and for *E. faecalis*, even a ST-based evolution rather than a high degree of HGT between these important hospital-associated clades.

### Availability of supporting data

The microarray data were deposited in the NCBI Gene Expression Omnibus (GEO) database (http://www.ncbi.nlm.nih.gov/geo/) under accession number GSE59190.

### Consent

The strains are not original samples, but pre-selected, diagnosed and isolated bacterial cultures and as such informed consent from patients is not required. Further, the strains were either given to the co-authors without Material Transfer Agreement (MTA) from originating sources, or given to the co-authours under MTA with the permission to be used in characterization of MGEs. Thus further consent is not necessary.

## Additional files

Additional file 1: Table S1. Correlation matrix for quantile normalized technical replicate (*E. faecalis* V583). Each fourplex microarray slide included the control strain, *E. faecalis* V583 to monitor the correlation between the hybridizations and to produce an appropriate cut off value. The overall correlation between the hybridization was 0.95, producing less than 1/20 false positive hybridizations. The grey rows and columns indicate (number of slide)_(sector on slide)_(number of hybridizations of slide) representing each sector hybridized with genomic DNA of *E. faecalis* V583. Additional file 2: Figure S1. A: PCA of the mobilome in 41 enterococcal strains. Each dot represents one of the 41 strains projected by their overall hybridization profile. *E. faecium* ST17 and ST78 strains are represented by blue and orange, respectively, *E. faecalis* ST6 and ST40 strains by red and green, respectively, while *E. faecium* ST92 is marked in purple. Strains are numbered as in Table 1. Each repeated hybridization of control strain *E. faecalis* V583 is represented by a black spot, B: Cluster dendrogram of the mobilome in 41 enterococcal strains. The dendrogram is visualizing the result of a hierarchical clustering calculation of the mobilome. Strains are numbered as in Table 1, and every application of the control strains *E. faecalis* V583 is indicated by a ‘C’. Additional file 3: Figure S2. Gene profile of the 40 clinical enterococcal strains. Hybridization results of 278 targets grouped into A: plasmid backbone determinants; B: transposable elements; C: resistance determinants; D: phage sequences; and E: CRISPR-Cas sequences in *E. faecium* ST 17 (n = 10), ST78 (n = 10) and ST92 (n = 1) and *E. faecalis* ST6 (n = 10) and ST40 (n = 10). Positive hybridizations are indicated by black boxes, no hybridization by white boxes. Additional file 4: Table S2. List of microarray targets and their corresponding probe sequence(s). The acc.no refers to the National Center for Biotechnology Information (NCBI) database (http://www.ncbi.nlm.nih.gov/), and the target names correspond to the locus names annotated in GenBank. Each target is represented by 1–5 probes in the DNA array as indicated. Additional file 5: Figure S3. Summarized gene profile of four enterococcal STs. Number of positive *E. faecium* ST17 (n = 10) and ST78 (n = 10) and *E. faecalis* ST6 (n = 10) and ST40 (n = 10) for selected targets within five groups; A: plasmid replicons and plasmid addiction systems; B: transposable elements; C: resistance determinants; D: phage; and E: CRISPR-Cas, determined by DNA microarray. Detailed information of each microarray target is found in Additional file 4: Table S2. Note that the presence of pIP501 *repR*, pCF10 *prgW*, pLG1 *rep*, and *aac(6’)-aph(2”)-Ia* are determined by PCR. The presence of ω-ε-ζ, *vanA*, *vanB*, and *vanG* genes are also verified by PCR. Chi square (χ^2^) calculations are done without corrections, and with 1 degree of freedom using the following online calculator: http://www.graphpad.com/quickcalcs/contingency1.cfm (last accessed December 10th, 2014). Where statistically significant differences (p < 0.05) between number of positive strains in each species (*E. faecium/E. faecalis*) are indicated by light grey (of the species with most positive strains) and between STs (ST17/78 or ST6/40) by dark grey (of the ST with most positive strains). White boxes indicate no significant differences. Verifying PCRs are not included in the calculations.
