# Erratum to: Investigating the mobilome in clinically important lineages of *Enterococcus faecium* and *Enterococcus faecalis*

**DOI:** 10.1186/s12864-015-1722-y

**Published:** 2015-09-15

**Authors:** Theresa Mikalsen, Torunn Pedersen, Rob Willems, Teresa M. Coque, Guido Werner, Ewa Sadowy, Willem van Schaik, Lars Bogø Jensen, María Victoria Francia, Arnfinn Sundsfjord, Kristin Hegstad

**Affiliations:** Research group for Host-microbe Interactions, Department of Medical Biology, Faculty of Health Science, University of Tromsø – The Arctic University of Norway, Tromsø, Norway; Department of Microbiology and Infection Control, University Hospital of North Norway, Tromsø, Norway; Department of Medical Microbiology, University Medical Center Utrecht, Utrecht, The Netherlands; Servicio de Microbiologia, Hospital Ramón y Cajal, Instituto Ramón y Cajal de Investigación Sanitaria (IRYCIS), Madrid, Spain; Centro de Investigación Biomédica en Red de Epidemiología y Salud Pública (CIBER-ESP), Madrid, Spain; Division of Nosocomial Pathogens and Antibiotic Resistance, Robert Koch Institute, Wernigerode Branch, Wernigerode, Germany; Department of Molecular Microbiology, National Medicines Institute, ul. Chełmska 30/34, 00-725 Warsaw, Poland; Division of Food Microbiologyt, National Food Institute, Danish Technical University, Copenhagen V, Denmark; Servicio de Microbiología, Pabellón 20, Hospital Universitario Marqués de Valdecilla e Instituto de Investigación Marqués de Valdecilla, Av. Valdecilla s/n. 39008, Santander, Spain; Theresa Mikalsen and Kristin Hegstad, UiT The Arctic University of Norway, Postbox 6050 Langnes, 9037 Tromsø, Norway

Following publication of this article it was brought to our attention that María Victoria Francia who contributed in the design of the array and provided target sequences was not included as an author.

