# Supplementary material for: Investigating the mobilome in clinically important lineages of Enterococcus faecium and Enterococcus faecalis
Source: BMC Genomics. 2015 Apr 10;16:282. doi: 10.1186/s12864-015-1407-6 (PMC4438569; doi:10.1186/s12864-015-1407-6)
Supplement: Additional file 1: Table S1. — Correlation matrix for quantile normalized technical replicate (E. faecalis V583). Each fourplex microarray slide included the control strain, E. faecalis V583 to monitor the correlation between the hybridizations and to produce an appropriate cut off value. The overall correlation between the hybridization was 0.95, producing less than 1/20 false positive hybridizations. The grey rows and columns indicate (number of slide)_(sector on slide)_(number of hybridizations of slide) representing each sector hybridized with genomic DNA of E. faecalis V583. [file 12864_2015_1407_MOESM1_ESM.pdf]

Additional file 1

	s2_1_2	s2_1_4	s2_2_3	s2_3_4	s2_4_1	s2_5_2	s3_1_3	s3_2_4	s3_4_4	s4_1_2	s4_3_3	s4_4_4	s4_5_1	s4_6_2	s5_1_1	s5_2_2	s5_3_3	s5_4_4
s2_1_2	1	0.98	0.98	0.92	0.99	0.99	0.97	0.96	0.97	0.97	0.94	0.96	0.97	0.97	0.93	0.91	0.9	0.96
s2_1_4	0.98	1	0.98	0.94	0.98	0.98	0.98	0.98	0.98	0.98	0.96	0.97	0.97	0.97	0.96	0.93	0.92	0.98
s2_2_3	0.98	0.98	1	0.92	0.97	0.98	0.97	0.96	0.97	0.96	0.95	0.96	0.96	0.96	0.95	0.92	0.89	0.96
s2_3_4	0.92	0.94	0.92	1	0.92	0.92	0.92	0.94	0.92	0.93	0.92	0.93	0.91	0.92	0.93	0.91	0.9	0.94
s2_4_1	0.99	0.98	0.97	0.92	1	0.99	0.97	0.97	0.98	0.97	0.95	0.97	0.97	0.97	0.94	0.92	0.9	0.96
s2_5_2	0.99	0.98	0.98	0.92	0.99	1	0.98	0.97	0.98	0.97	0.94	0.97	0.97	0.96	0.93	0.92	0.89	0.96
s3_1_3	0.97	0.98	0.97	0.92	0.97	0.98	1	0.97	0.97	0.97	0.95	0.96	0.96	0.96	0.94	0.92	0.89	0.96
s3_2_4	0.96	0.98	0.96	0.94	0.97	0.97	0.97	1	0.99	0.98	0.95	0.96	0.96	0.96	0.94	0.92	0.91	0.96
s3_4_4	0.97	0.98	0.97	0.92	0.98	0.98	0.97	0.99	1	0.97	0.95	0.96	0.96	0.96	0.93	0.91	0.9	0.96
s4_1_2	0.97	0.98	0.96	0.93	0.97	0.97	0.97	0.98	0.97	1	0.96	0.97	0.97	0.98	0.94	0.93	0.91	0.97
s4_3_3	0.94	0.96	0.95	0.92	0.95	0.94	0.95	0.95	0.95	0.96	1	0.98	0.97	0.98	0.97	0.94	0.92	0.98
s4_4_4	0.96	0.97	0.96	0.93	0.97	0.97	0.96	0.96	0.96	0.97	0.98	1	0.98	0.99	0.97	0.94	0.92	0.99
s4_5_1	0.97	0.97	0.96	0.91	0.97	0.97	0.96	0.96	0.96	0.97	0.97	0.98	1	0.99	0.95	0.93	0.91	0.98
s4_6_2	0.97	0.97	0.96	0.92	0.97	0.96	0.96	0.96	0.96	0.98	0.98	0.99	0.99	1	0.95	0.93	0.91	0.98
s5_1_1	0.93	0.96	0.95	0.93	0.94	0.93	0.94	0.94	0.93	0.94	0.97	0.97	0.95	0.95	1	0.95	0.92	0.97
s5_2_2	0.91	0.93	0.92	0.91	0.92	0.92	0.92	0.92	0.91	0.93	0.94	0.94	0.93	0.93	0.95	1	0.95	0.95
s5_3_3	0.9	0.92	0.89	0.9	0.9	0.89	0.89	0.91	0.9	0.91	0.92	0.92	0.91	0.91	0.92	0.95	1	0.92
s5_4_4	0.96	0.98	0.96	0.94	0.96	0.96	0.96	0.96	0.96	0.97	0.98	0.99	0.98	0.98	0.97	0.95	0.92	1
