# Supplementary figures and images for: Investigating the mobilome in clinically important lineages of Enterococcus faecium and Enterococcus faecalis
Source: BMC Genomics. 2015 Apr 10;16:282. doi: 10.1186/s12864-015-1407-6 (PMC4438569; doi:10.1186/s12864-015-1407-6)

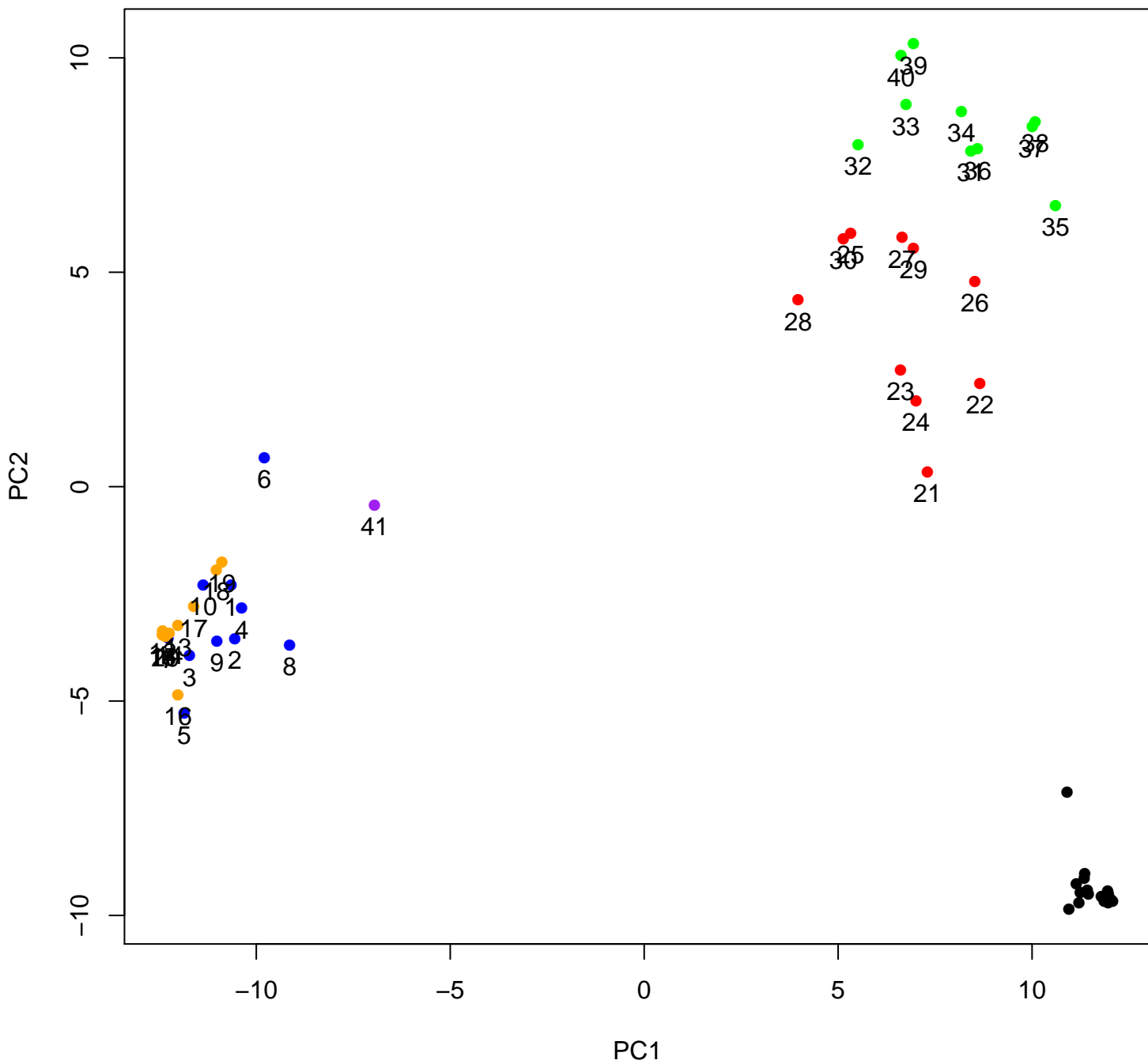

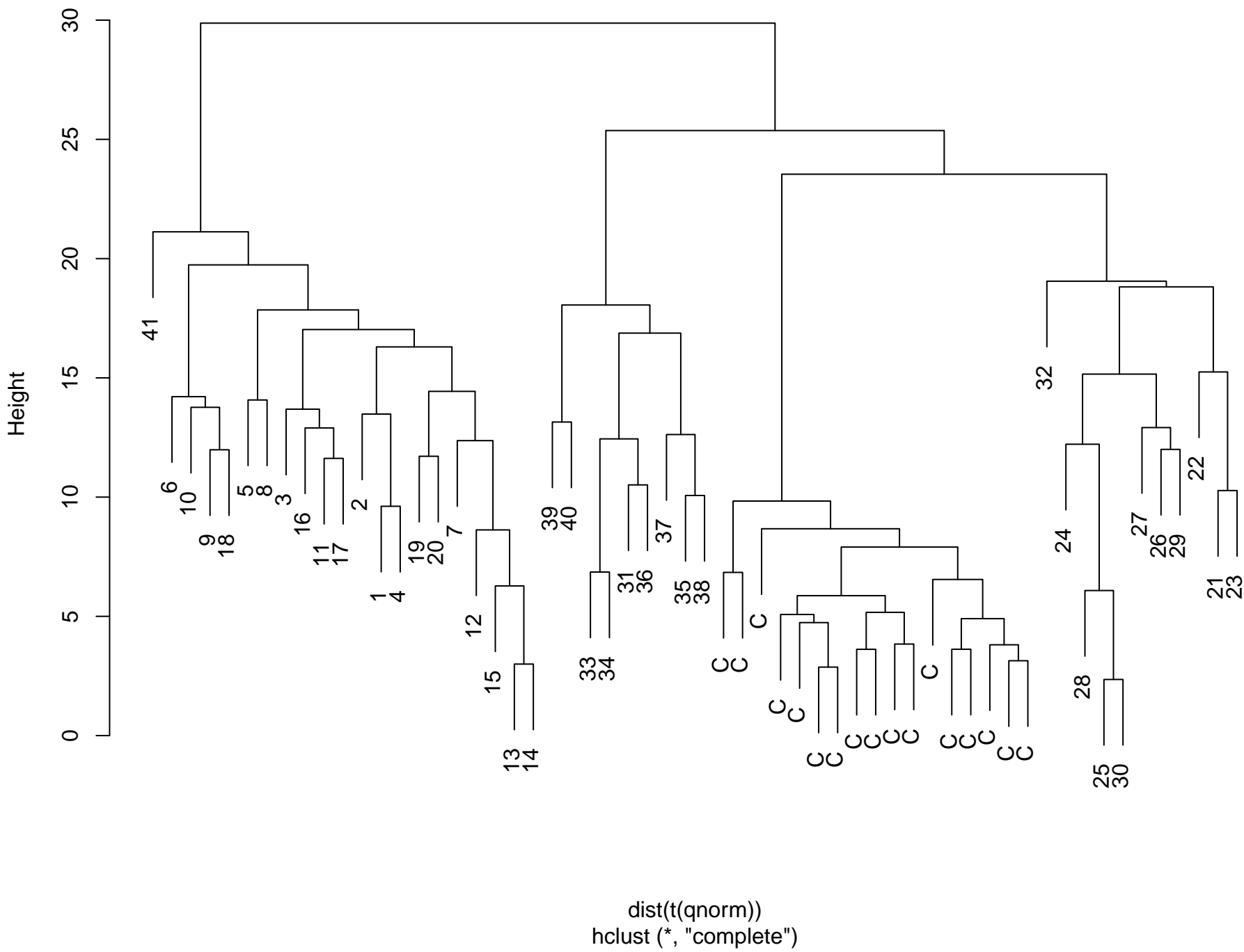

Supplement: Additional file 2: Figure S1. — A: PCA of the mobilome in 41 enterococcal strains. Each dot represents one of the 41 strains projected by their overall hybridization profile. E. faecium ST17 and ST78 strains are represented by blue and orange, respectively, E. faecalis ST6 and ST40 strains by red and green, respectively, while E. faecium ST92 is marked in purple. Strains are numbered as in Table 1. Each repeated hybridization of control strain E. faecalis V583 is represented by a black spot, B: Cluster dendrogram of the mobilome in 41 enterococcal strains. The dendrogram is visualizing the result of a hierarchical clustering calculation of the mobilome. Strains are numbered as in Table 1, and every application of the control strains E. faecalis V583 is indicated by a ‘C’. [file 12864_2015_1407_MOESM2_ESM.pdf]

**A**

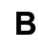

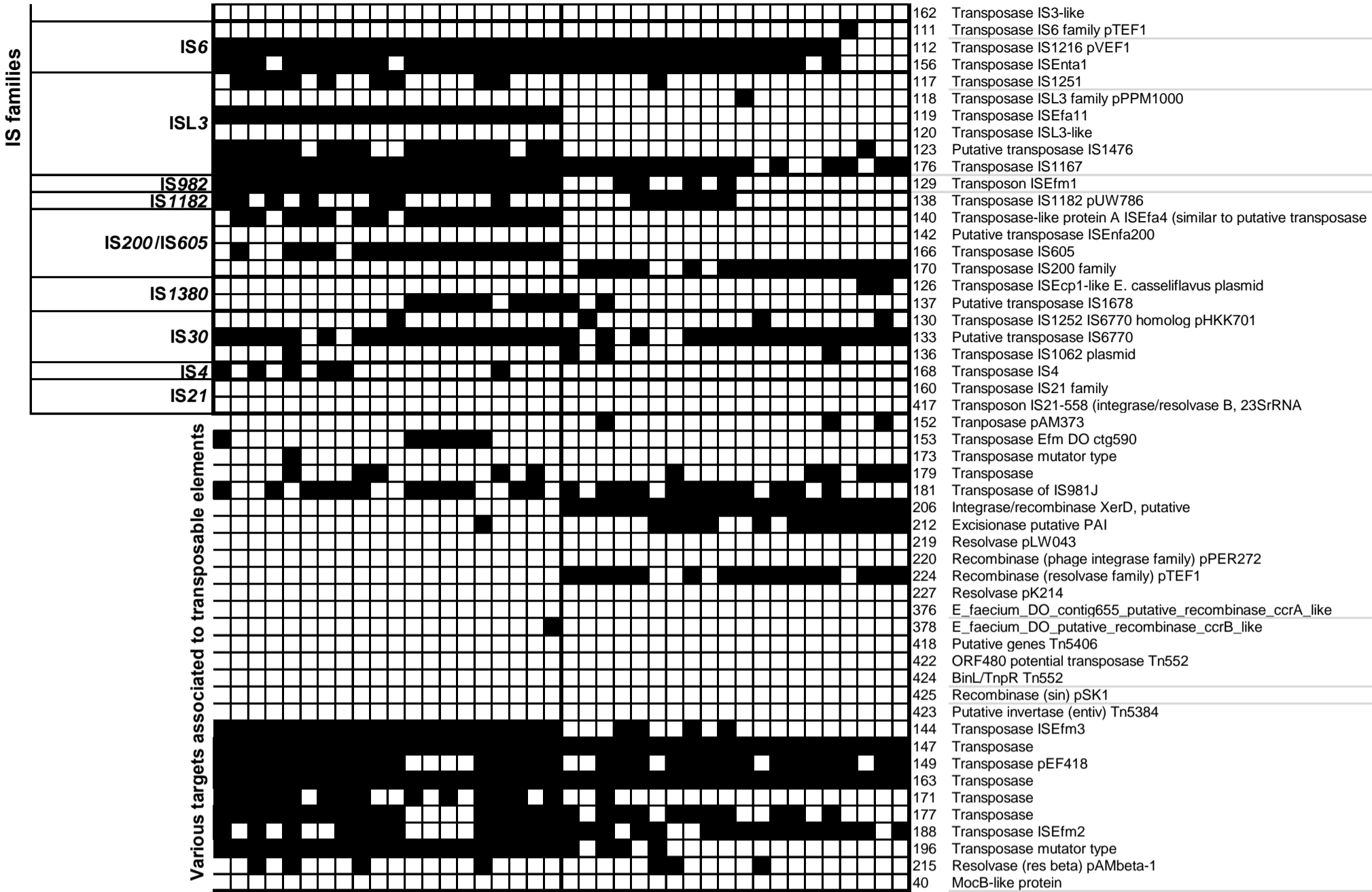

C

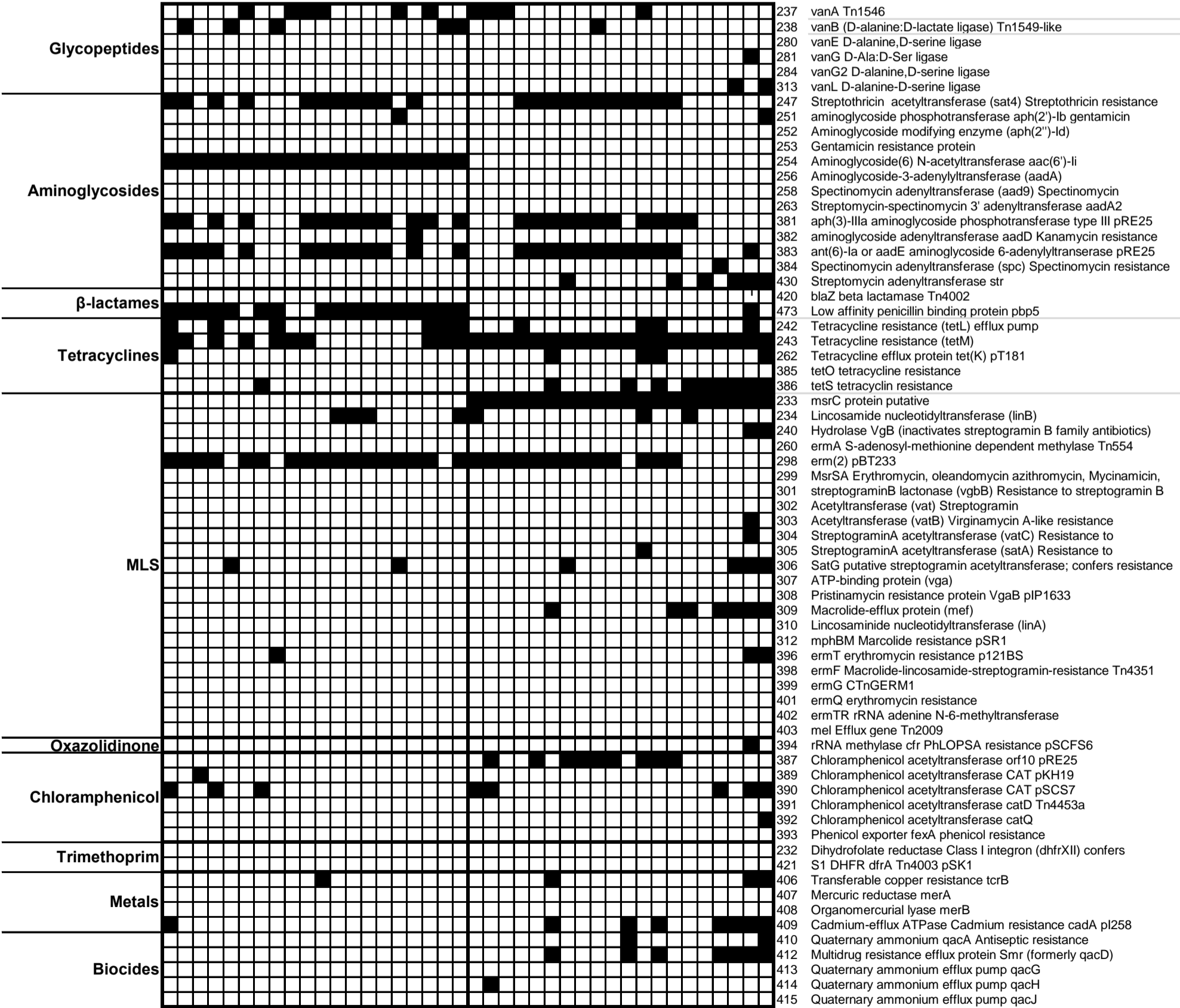

D

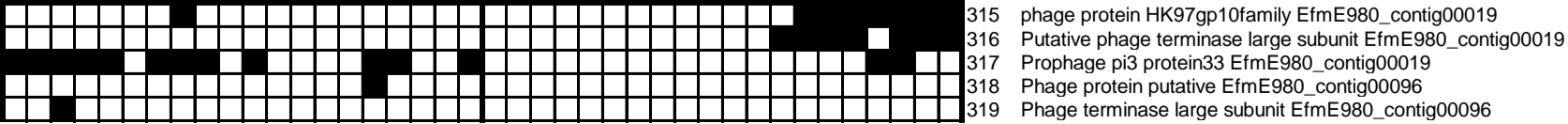

Supplement: Additional file 3: Figure S2. — Gene profile of the 40 clinical enterococcal strains. Hybridization results of 278 targets grouped into A: plasmid backbone determinants; B: transposable elements; C: resistance determinants; D: phage sequences; and E: CRISPR-Cas sequences in E. faecium ST 17 (n = 10), ST78 (n = 10) and ST92 (n = 1) and E. faecalis ST6 (n = 10) and ST40 (n = 10). Positive hybridizations are indicated by black boxes, no hybridization by white boxes. [file 12864_2015_1407_MOESM3_ESM.pdf]
