# Supplementary material for: Investigating the mobilome in clinically important lineages of Enterococcus faecium and Enterococcus faecalis
Source: BMC Genomics. 2015 Apr 10;16:282. doi: 10.1186/s12864-015-1407-6 (PMC4438569; doi:10.1186/s12864-015-1407-6)
Supplement: Additional file 4: Table S2. — List of microarray targets and their corresponding probe sequence(s). The acc.no refers to the National Center for Biotechnology Information (NCBI) database (http://www.ncbi.nlm.nih.gov/), and the target names correspond to the locus names annotated in GenBank. Each target is represented by 1–5 probes in the DNA array as indicated. [file 12864_2015_1407_MOESM4_ESM.pdf]

Additional file 4

Plasmid backbone	Index	Annotated gene name/Information	Species	Acc.no.	Locus	Probes	Replicates	Probe sequence(s)				
Replication genes	3	CDS1 putative rep pRE25	E. faecalis	X92945	33-1526	5	2, 1, 2, 2, 1	aacaaggatcacagaggtatttggactcaacgagagc	ggcggagaaggacgtcttggtcgaaataatgtgat	tcagtcaaaacgttgttcgcgagggttattcaact	tggatctaggatgtaaccacttcggaattgctcga	ttggcttcacaactaacgatgcccaagcgaacact
	4	Putative repS pTEF3	E. faecalis	AE016832	16433-17938	5	1, 1, 1, 1, 1	acaagaccgctgttttgatgatatggatgtattcaacagt	agcactgacacatgatatgcaacaactaatccagtgg	gaaaagcatttgctgaagtattgccttcggtcgatt	gcaaaacctatgtgcaagaactagtgtcaaattgggga	gcggctgttactttcaatgggcaatcgattcctaa
	5	Putative repA pRUM	E. faecium	AF507977	20542-21582	5	2,1,2,1,1	cgattttcgcaattacggttgagtccttgatatgactgg	cgcaattacggtggtatcagtattgtcttcctcttcc	gacgtcttgattcgttacttctggcttgcctaagt	tcctttgtcagcatcaattccataagattccagttcttca	tcgcaattacggtggtatcattgatttctcgcggt
	6	Replication associated protein pAM373	E. faecalis	NC_002630 (AE002565)	1725-2741	5	1,1,1,2,2	agggattcagctgatatggtacggtgtccgatttt	caagggattcagctgatatggtacggtgtccgatt	cgattttcggattcccgcttatatcaagggattcagc	tccagttgttctttaaggagtagtccaatcgatctcgt	tttcggattcccgcttatatcaagggattcagttgttt
	7	Rep protein pS86	E. faecalis	AJ223161	500-1222	4	1,1,1,1	aatggtttaacaacagagtttagtaggtttgagctgtccg	accaagcgtaaccatgcacgattgggtaaacggag	actggtctgaaaaccaacaaaccaagcgtaaccatgc	gctgtccgcttttacttcaatacgctcaacatatgct	
	9	repA pEF418	E. faecalis	AF408195	198-761	5	1,1,1,1,1	acaattccgttgcgaaaatttacaccagtcgaaatgaa	atgggtgcgctattctctccaacagttcaatgaat	ccttttgctgaaatacaagttcatgaaaaagccttaccct	gccttacccttacttaataatcttgaagaatgggtgcgc	tacgttttggtagaagaagtgctgacggtttgcaa
	10	repA pEJ97-1	E. faecalis	AJ490170	6874-7857	4	1,1,1,1	acaatcaattgctgaaaacgttgataacagcggaaatgt	acagcagcgtgaattattacaatctttctctgaaaccca	gaagcacacaatgctgtcggtattattattcgggca	tcacacttccgaaaatcgttgataacagcggaaatgt	
	13	repA pEF1071	E. faecalis	AF164559	7837-8820	5	2,1,2,2,1	aaacatgcccctactaaaatcgaacagagtcaggc	atcgaacagagtcaggcgttatctcaggtaaacgg	gcacttcctctgttaaatgacttagattcttgggttcgc	tcgcggtaaacccgttattggttatcaattcacgt	ttgcgattcggcaggagaagtcaaagtggcttaac
	14	repA-1 pTEF1	E. faecalis V583	AE016833	101-1111	5	1,1,1,1,1	aatcataccctgccaaaacgttgataaatcagagggtatg	acgctgaatcccttggtaatcaagagggtatgaaaatca	agctcatgagcaagtaggaacaattattcgtgctaaaact	cacaccaggagtaatcactcataactgggttgaaaatca	gctgccttctctaatactattcaagaagctcatgagcaag
	16	RepA replication-associated protein pTEF2	E. faecalis V583	AE016831	100-1110	5	1,1,1,2,2	accaaccttcggataaacatgataaaatcggcgtttct	agcaacagatgtttacgccatcagtaatgctagct	agtaaaatagcttacgcaatgttgcaagaccgatgtga	tcggcgtttctttcgaaagtgggaaaaaccaacgtcca	ttcaagaagcccatgaaatggttggaacaattattcgtgc
	17	RepE pAMbeta1	E. faecalis	AF007787	3114-4604	5	1,1,1,1,1	agagaagtgctaggcattcctgaacggacattaga	caagctagcagaacgccctaaaacggacacacaact	cctactttagttcaggctattcaatcgaaacgtgcga	gaagcgaacgtcaacgtgttcatttgtcagaatgg	gggtttaatagggcgtaataacgtcatgtttaccctctc
	19	replication protein pEFNP1	E. faecium	AB038522	439-1206	5	2,1,2,2,1	aacatggtggcgtatagaaatgcagctaagacgtg	gtcctcattacttgcctggagatgtaaaacctgttgat	gtcggttggtacaaatggttggcaattatttgaccaa	tgcagatattgcttgcgatattctcggtgttcctg	ttggcatgcgatggttcgggaaagtttagatagctt
	20	Putative plasmid replication protein pCIZ2	E. faecium	NC_008259 (DQ832184)	5690-6427	2	1,2	gattacaatataaatgcacgtggcgaccgttttgct	tcgcatgagagatattgataaacaaatttttgcccaagcg			
	21	ORF pKQ10	E. faecium	EFU01917 (U01917)	392-727	5	2,1,1,1,1	atgcgatggttcaggaaagcttagatagttttgcaagc	gaattggatacgtggcttttggggttggatgtgac	gctgaagcaagaaagtcaaaatgatgagttaacgaaccat	ggtttgatagctgagccttctaattggtcgataattgct	tggcggttagaaatgcagctaagacgtggcaaagc
	27	replication protein pHT beta	E. faecium	AB183714	49773-51374	5	2,1,1,1,1	aaagggaatcgtgtcgatccgtctaattgggtgaa	aagtggcttatctattggcgagtgtatggatcaattgg	acggcgaatacagtgatattacaaacgtctcgtggt	agtgcttatcgtggcgattacaaaggtgctaaaagt	gcaaacatgcgccaattagaggagctaatacaggt
	28	rep protein pEFR	E. faecium	AF511037	2304-3176	5	2,1,1,2,1	aacatttcgtaggaaagtccagattcacttctacgaccaa	acaagcggaacatcgttttagagtagctgctttgc	gctttgcagctcgttgaattgttggagagaataacg	tcagttggtcgaacgtaaatcgaacggttttatcgc	tgcggttagttcttgacggataggcttaataactctcttg
	30	repA pB82	E. faecium	AB178871	4157-5101	5	1,2,1,1,1	aaggggataagacagttcgttttacgtttgaccaact	acggggaaaacctgttattggctatcaatttacgttca	agtggctatgcaaagactatgtttcgtctgttaaagca	gcactgcctttgttaaataatttagacgagtgggtacgc	tggtcgtagaagtgagtctgggctttcttacgatatgtt
	31	repA p200B	E. faecium	AB158402	7568-8500	5	2,1,1,1,2	aaagatttaagtgcgtataagcagactgcaggagaagtc	aggtcgaggtaaacctgttattggttatcgttttacttgg	caataccgaactcaaggatgggctgaattttctaagga	ggcaagaacatacgatcatttgatggatttacgtttcgg	tggcgtgctaaagatcgaatactaaatcttccgttaggt
	360	CDS 11 putative Rep pRE25	E. faecalis	X92945	9712-10650	5	1,2,1,1,1	acttagctttccccacatactttcttcatgcaacaaagt	tcccaaagtcgtatttgtggctctacactcataaagtca	cgattgtcatggcatcaaaacttaatgcgggttga	aatgcgggttgagattttcccaaagtttgaccact	cccaaagtttgaccacttaaccggctattacttaaccg
	361	putative replication protein repB pMBB1	E. faecium	U26268	1045-1869	5	2,1,1,2,1	agcatttctactcatgattggctgaatggaggcaa	ctcctgaagagtttaaagagtggttaggcgctcca	gaaagccttgaatctatgagagctaaaagcgttcgtgt	ttggcggaccgaccgtaattttgcattagatgaat	ttttacaaggtcgatacggtcgcaaagtggttcaa
	362	rep pN315	S. aureus	AP003139	232-1215	4	1,1,1,1	acttgaagaaacacatgccctttatatgcgccgta	agaaaacgatggtctactcattcaaaaacgacgtggtt	aggccaaaaggaaacaatcaagtcaatgcaagcttatt	gatgttgaaccttacggcgagaaggacgttcgatt	
	363	replication protein rep pN315	S. aureus	AP003139	21570-22430	4	2,1,1,1	tggatagagttcacatttgacgctgagaaacgcat	gacgctgagaaacgcattcataacaaacgacaacc	cgtgaaaagacgcctaaatggttagaagaacggtcata	acggtcatatgaaaagcaacctcaaaaagattacgaccc	
	365	prgW pCF10	E. faecalis	NC_006827	3035-4036	2	1,1	acctgctatatctcggaaaacctaacgttacttcagaaga	agccctgcaacaacagttattgctcgatcaattttc			
	366	replication protein pIM13	B. subtilis	M13761	389-829	5	1,2,1,1,1	acctcttactcgaatttgggaactttgagcaagagg	agaaagatatggaacagtctataaaggctctcagaggctc	agttataccgtaaacaaacgtctggtaacttcgtaaaggc	ggctctcagaggctcatagacgaagaaagtggaga	tgttaaaccctgaactactaatgagaggcgacgacc
	367	replication protein pSK6	S. aureus	U96610	770-1234	3	1,2,1	aagctatatagagcacaaacaggtggaaacttcgtgaag	caggtggaaacttcgtgaagatgtatatggaactgtttgg	tgataaacccagccttattaatgcgtggagatgatagc		
	368	replication associated protein pBMB165	B. thuringiensis	DQ242517	18202-19068	5	2,1,1,2,1	aaagttctaacagttgatatggacccgcaggccaa	ctgtagcaaacagtaaaggtggcgttggtaaatctact	acatcaagattcggtcatgctgatccttctgatcct	tgcaatggcaagtgactatgtactcgtagcattcc	tcaaagcattccacgtgttggcttaaatacggatca
	369	replication associated protein pBMB165	B. thuringiensis	DQ242517	19061-19441	5	1,1,2,2,1	atcactccctctacaccatatgatcctagtttacctgc	cctgcaaaagcaacactagttaatagtgataagcgtggt	gcgtggtactattcgaatgcctttagaaactaaacacga	tgtcacctgaacaactaagattattccatgcacaacttga	cctgggactaaaaatacaggagggaaaattgatattggct
	370	putative replication initiation protein pSSP1	St. saprophyticus	AP008935	22985-24250	2	2,1	actgcgtgatgccaaatcaagaaattgcaaaatttttgaa	tgcgctctattttgtccgaattagaagaaatgcgct			
	371	replication initiation protein pRI1	E. faecium	EU327398	3161-4114	5	1,1,2,2,1	actgcaattctagtcgccaccatgtcgttatttcc	cgattccgcaaatgactctcgcaaatgattcgtca	tcagcacctacttccgttacatcaagtccttggag	tcgggaatatctacaatatcgcaagcaatatccgcac	tggtctaataattgccacccattattcccaacagct
	372	replication protein ori43 pBMB67	B. thuringiensis	DQ363750	21225-22757	5	1,1,1,2,2	acacttgaagtacaatttcgaggccaaacaagcga	atagagatggtagagcagcaatgcacgtctgatgc	cagcaaaggaacgcttagaccgtcagcaaagccat	tggacaagcattgaactcagaatgggctttagcat	tggatgctagtgaagtagaagaaggaactacaaagcct
	374	replication intiation protein repM pRUM	E. faecium	NC_005000 (AF507977)	15306-15854	4	2,2,1,1	aaatgcagatgttgatgtaagtgctgaacatctatggcg	tgctgaacatctatggcgtgttgaaattgaacttaaacg	cacattttgaaacctgcatgggctactttggaaagt	agcgaactgtatttttcggaacaactggtaaggca	
	375	replication initiation repA pUB101	S. aureus	AY373761	3335-4519	1	1	accgaagacctgactattcccgtagttaggtctct				
	431	RepD E. casseliflavus plasmid	E. casseliflavus	AY939911	7143-7982	5	2,1,2,1,1	aaagcagatacttgggacagacgtaatatgcgtgttgaat	aacagaacatcatcgactacatggaagatgacggttttac	agttttgacgctatgacaatagttggaaatctcagtcgtg	caatgcagatgttgaagttatgtctgaacacttatggcgt	ctcagtcgtgacaacgctcaaaagctatcgtcatt
Genes associated with plasmid conjugation	41	Rlx protein pRUM	E. faecium	AF507977	18806-19192	5	1,1,1,1,1	cagatggcaacgaaggacatgtggttattcagtct	acctaacgaagtaacgccagaacaatgtaatcaattaggc	agccgaaaagatagcacctaatcatcaagttgccg	catcaagttgccgtatatacgcataacgacacagacc	acatcaagggctattaattacgcagagaaacgggc
	42	MobA pEF1071	E. faecalis	AF164559	5841-6881	5	1,1,1,2,1	cagaccatatccataaccatctggtgatcaatgcgg	gccaccataacgactttcagcgcgtatctgccctc	gctctataagctcatataaggcgtttagagagggtttgga	ttagtcgaggaagtgacgaaattacgcacgatcca	accagatcttacaccacgacaatcaggtccttctc
	45	mobA pB82	E. faecium	AB178871	1465-2379	5	2,1,2,2,1	ttcgcatgctgtatggaaaagacgatcgtgtacaag	gtaagccgaaccacttcttgtagccgagcgatcaat	aaccacattgtcattaactccgtcaatctggaaactgg	tgtcttgaacatgacttaacaatcactgaacgagggaag	cgagaacgagcctacaatcaacaacgaaccaacga
	46	hypothetical protein pEF1 (relaxase/nickase)	E. faecium	DQ198088	20444-21283	5	1,1,1,1,2	gacgtacgcccgaggaagaacaggaactgcgtctaa	accagcagtcattctcacttgtcaatttaccttgcc	acgtcgaatgaagcgcaatcgttgatgaaacatgg	acaggctactggatggcaacgattcaccgaaatac	tggtgtgagttatatcgtaccgaaagggaagtgga
	48	putative relaxase/nickase pHTbeta	E. faecium	AB183714	31532-33052	5	2,1,1,1,1	tgagctatatcaaacaacctacggtaaacgctacaatcca	gctcgaatgggtaatgctgtagtaggacaaatcaaact	atcgtgcgaaaaatgattatgaagtagcgcaacgaatg	acaagctaggggtaagagaaaacagtctacgttagacg	gaaatacctagtacaacacgtggctacaatgagcttcc
	49	nickase pRE25	E. faecalis	X92945	19637-21601	3	1,1,2	tcagtcatagaaggtgaaatgttacttgcaaaagctgact	aagtcgaaggagttgctagattcagcagtgaggaaattaa	tttgcgaagtcaattaatcgtttgttgagtggtaaccaac		
	433	relaxase pTEF2	E. faecalis V583	AE016831	30365-32050	5	1,1,2,1,2	ggctatggcaattctggaaagctctttgtaggagggtat	ggagggtataaggttgatccattagccaacggtaattaca	aacctgtgatgcaaaccattgatgaattagttagtgcca	actcttgacgagaagatgttagagctgcaccaattaagt	tttctaataagtggacgacctatcgattgcttgacgaacc
	435	MobE mobilization protein pAMalpha1	E. faecalis	NC_005013 (AF503772)	6710-8389	5	1,2,1,2,2	acgggttaactacttaccttcacgaaagactaggtcatgc	tttacgattcagcaaggagtgaagtaccttcagaacgcg	acggggtcaaaaggggtaacaatgtccttaaacccaca	aacagggtgttatgagtttgtcggcgaatatatggct	tggctaatggacgttctcgaacgaatactttacaactgtc
	436	MobB mobilization protein pAMalpha1	E. faecalis	NC_005013 (AF503772)	2001-3263	5	1,1,2,1,1	aggaattccttaaggaacgtacagacggcttaaaagc	ctcacatgcatttaggtgttgtgcctatgcgtgat	ttgaagcgtggtgaacgtggctctgaccgtaaaca	tcagatttagaagtaccagcgaaacgacacatgaaaagt	gtgactgctgcgagagataacgataggttaaaacagc
	438	mobilization peptide pMV158	S.agalactiae	MV1MOBPA	628-2112	5	1,1,1,1,1	aagcgttgaacgagagtgagttgcgagaactaaagc	agtcctgaacaatttgaagggttgaaggcagaagt	tagatacatgagcgaccacggttttgagttggaac	ggaacgtggcaagctgaatagtgaagctaagcataa	tcaagagggcaatggctgatatggaactcaaagag
	439	orf	S. ferus	STRMOPR	1054-2348	5	1,1,2,1,1	agaatttaaagggctgatataccgaagcagaaaccttgt	gatagtgcctctgagacgctctaaacggtttgaag	tggtgagtccaatattgcttatgcgagtgtccact	aggacttgcctagatacatgagcgaccatggtttt	tcaagagggcaatggctgatatggaactcaaagagg
	440	Orf57 pAD1	E. faecalis	AH011360 (AF343837)	8542-9317	5	1,1,1,1,1	agaagggcataattacattggtgggtactacaccttgc	gtgggtactacaccttgccaaaagttcctgaatacaattt	caccttaaagcggtagtatcagagcgtcgcaaagtatatg	taaagacaaaacgacgttatagtcagggtgtgtttccaaa	acaaagaaggggacgagtacaaagaagtgtttgatcga
	76	ATP/GTP-binding protein putative	E. faecalis V583	NC_004668 (AE016830)	1823909-18263	5	2,1,1,2,1	tggaatttgtctcattatcggattggtaggcatggtt	taaatacgtcagatcactcggtgtgacccgtctgact	tactaatgcatctgtttgaaggtcaattcgttgtttcgcg	tcttgccttcacgattctgagcaatcaattgacgaaa	cgtgcacttgatacttctgttcaggcgataagaagg
	78	ATP or GTP-binding protein putativ	E. faecium DO	NZ_AAAK03000055	14038-16467	5	1,2,1,2,1	gaaattgcggatgaaatcagtatcctcaacttgacgagta	tgttacgtaaagccattcgaacggtgactcaaagt	gttattaacgaattgcgaaaagacgggtcaccaatcg	tggaattgcttagtgtttcgatgctaatcgtcatttccac	gttcgtgtagactttccttggtgtatccaacggcg
	79	conjugal transfer protein putative	E. faecalis V583	NC_004668 (AE016830)	155767-15827	5	1,1,1,1,2	acggcgattaatgaaacagtagcagaagtggtggc	ggctgaacggaaagcaggcaaacaagttggatttt	agatcggcacgatgaattagacgagaatcaacggtt	acgcaatcggtcaacgatgtgttagataatggcga	tgaaacagttgatgatacagaagcgtccgttatggaaa
	80	conjugal transfer protein putative	E. faecalis V583	NC_004668 (AE016830)	2441451-24439	5	1,2,1,2,1	cttcctgcatgatattcttagtgcgggttcttgca	tctcggtaatcctttcccaatattctgctagttccaca	ggcacaccaataatccacgtatattcgtaaaccataccc	tttccctcgaatgtcggcattaactggcaaaaggt	acatcagtcaacattaccgtttcagaacgaacacga
	81	traE protein putative	E. faecalis V583	NC_004668 (AE016830)	2244004-22464	5	1,1,2,1,1	cgcttgatgttcttgattgccgcgctctggtcgat	cttgaaattcgccagcacatccgcttcaatccgtt	aaaggtgatgtacttggtctttgtcaggccgttgtt	gtcctcgttctgtgcaagctggtagttcacatcat	gtgaaatagcggtcgcccacgatacagataccgtc
	83	TrsE-like protein	E. faecalis	AY271782	11122-13455	5	1,1,1,1,1	atatgaactccgattacggtgacggtgcaaatcct	atccattattgaccgatgtacggcaagcgtgtacc	tacagcctatcggtatgcttgtggtacttgacagc	taatcaagcgtctacggacagacttgagcttgcca	gctttacaagttgatgacgaccaagttcggtgagg
	85	vancomycin resistant virulence factor gene	E. faecium	AY273800	1-924	5	1,2,1,1,1	agtgtgcggttgtctaccaataccttgcttctagg	ttaaaatgatcgcggaagcccatttagctgcagga	tatggtgcgtttagacggatctgacgggatgatca	caaagtggtctatcaggtacagttagtgagtcctcagc	atgaagagtatggaacagtaccacgcggtttcact
	87	hypothetical protein pHTbeta	E. faecium	NC_007594 (AB183714)	25385-27340	5	1,1,1,2,2	cgtgttcgttctattgaactaattaagctgatcgtgacgg	caattatttacagcgttaacgaccatttggaacagtgcc	gcacaagattttatggttttgatcgatgagtgtcacaacg	tccctaaagattctggatcagaagtagcagatgtgatcag	tccagagcaaattgaacgattcgatggtggtcaat
	88	hypothetical protein pRE25	E. faecalis	NC_008445 (X92945)	23016-24977	5	1,1,1,2,1	cagaccgaagaagggatcagagatttagcatacctcaac	caacaatccgcaaattgtaacgtctgtttatgcagatga	aatgctgattgtaccgtatcaacccaacagattgatgat	tcttcggacttgatgaaacggaagcacaaggtattt	agaaatgcgtttgcttaacccaggcgaatatgaaga
	91	hypotetichal protein pTEF1	E. faecalis	NC_004669 (AE016833)	24672-27164	5	2,2,1,1,2	tccggaagtaacgttcgttcaggtaaacgggtattt	aaacaatcgacctatggttcttggatccatgcgttc	cggaaataggtttctcctaaatccgcaaagcttccc	taaaatggtagtcgaacgtgtcttgctcgaatacgc	tggtgtgcaagtttataacctacccacacgtcgtta
	446	prgJ VirB4-like pCF10	E. faecalis	NC_006827 (AY855841)	21359-23596	5	1,1,2,1,1	accgattgggaaagaattggatgcagaaatactcgatatc	atggtagacaagcagttattggatgaagaggaccaggaa	tgtgggttcacaagatatctttgcgcaccaaacga	cgtattcgtaagtatggcgccgtgacaacaggtat	caggtatcacgcaaaaccctacgacgttattagacagt
	448	hypotetical protein VirB4-like pAM373	E. faecalis	NC_002630 (AE002565)	19565-22057	3	2,2,1	tgacaagaagacgctaattttagattctgtcaatgcggtg	aagacacggcccaaggacgttttctaattgaaggaa	ccaagaccatcgaggacgtagccagcctattgcaat		
Plasmid maintenance	55	ParA family protein pTEF2	E. faecalis	NC_004671 (AE016831)	56369-57151	5	1,1,1,1,2	gccactgataattttggattcgacattgatggcacg	tcagcagaacgtgaatttacacaagttggaagtgaaca	acaacgactagcattaaccttggagcggcattgca	acattagacgaactatcactgttgatgaagcccaagc	tccgacttgattagctttaataagacttcgaccgctga
	56	putative Soj partitioning protein pRUM	E. faecium	NC_005000 (AF507977)	22244-23047	5	1,1,1,2,1	gcttgcgcttcgtcaacggttattgtcctacgaat	accaatgactctagaaatttcggttgctgcagacg	agtccttttaatgagaaatacgcagcttcaactggaatga	tgcgttgactgtaagcattcctaaacttggtggac	taagcctgtgttcacttcctacttgagtaaattccctct
	57	ParA family ATPase Efs V583	E. faecalis V583	NC_004668 (AE016830)	3181033-31817	5	1,1,2,1,1	ggcacctaaattggtccgagcatcgtacatggtta	accaagcgaatcgtatttagcaattgactcaagcct	tgcttgatacacttcggcaccttttgagcgaatat	aatttcaagcgcgattctcgagccatcattgaagt	catcgtgtgcgacatctggtttaggaacacccatg
	59	PrgP pCF10	E. faecalis	NC_006827 (AY855841)	1859-2788	5	1,1,1,1,1	cccaccaacggcgatcaacataatcattaagttgga	gtttctccataagtttccagcctatcagagcctttaacaa	gttgtgactctcgacgtgccgaaaacattacagga	accgtttaagctcttgcatcggtataacataatcagca	accacccttacgccctgttagcattattactttacct
	60	putative PrgP protein pRE25 (Orf 58)	E. faecalis	NC_008445 (X92945)	48704-49657	5	1,2,2,1,1	ccatacatttctaaccgatccgagcctttaacaatgcttt	tccaagaggtaggatatgattggccattgagtcagc	aagaatgcacgaaccacattatctgttgatggtggg	ggacaagtattcattaagttcttcagatccaggagagcca	taccaatgccgccttttctacctgtaagcattacaa
	61	repB pAD1	E. faecalis	L01794	1606-2451	5	1,1,2,2,1	cggcgttgggaagactactaattctgtaatggtagc	attgttttgcaaacgcagcaacgctcccttgatgg	tcccttgatggtgcagaagcattttgggaatactt	ttgactttgatatcgctggggtacttccagtgctg	taaagatgccaaagacgcttttggtgatgagactttgtt
	63	Cobyrinic acid a,c-diamide synthase	E. faecium	NZ_AAAK03000001	30170-31027	5	1,2,1,1,2	gtaatacctgatccgtcgtatcgttttaagcgcgc	ttacttgggtctgtaagacgacgattgcccaatcc	acacgtcagtcacagtattttcgtaaacaatcggttca	gcctccttcttcgttagtgaagttcatatgcgttaagt	aactgctgattcaaaccctaacatttgcgcgcacg
	64	replication-associated protein RepB pTEF3	E. faecalis V583	NC_004670 (AE016832)	14875-15696	5	1,2,1,1,2	gcagtgttagctggaatgagatcaaggttttctgaga	tctgagatattggtgattgctggttcaagactaccttct	tcagtaacagtataagtgatttcggctaaatctcccgtct	accttatgcgctttaacgtcccaaccgtctgcttt	tctcccgtcttagctaatactctagtagcgtttccttga
	65	Cobyrinic acid a,c-diamide synthase	E. faecium	NZ_AAAK03000024	24463-25275	5	1,1,1,2,1	cactggtaatagatattgatccgcaagccaacgct	agtgcatattcagataatgctatggcggcctctga	aagtgatatctataattgcatgcatgcttgaaccagacga	ttgagcgaggctatggatttatatggatccgctgt	cgaaacggaaattatgatcaatgggactatagggcaca
	66	ATPase pVEF1	E. faecium	NC_008768 (AM296544)	38165-39076	5	1,1,1,1,1	accaaacttttcgatagcatcattgacaacttggcgatc	caacgccatctaagctatcagattgggtttgaaaagcca	gcgtaagcagcattcctagtgaaatcagacatagtaggt	tcagcgtctattccttctatagcgtccccataaagct	acatcgtctttgtagtatttgattgcggatcggca
	70	Cobyrinic acid a,c-diamide synthase	E. faecium	NZ_AAAK03000051	4362-5129	5	1,2,1,2,1	ggaaatgctaccagtggtgtcgggatcagaaaacc	tttgagtattgtgcctgcgactctccaattagcag	acaacgacgacggtgaatctaggtgcttgtcttgc	tcgagggagtcttactgacaatgtatgatgcacga	accgatcatcgactacgatccacgttctcgaggag
	71	RepB protein (copy control and partitioning) pEJ97	E. faecalis	AJ490170	5492-6238	5	1,1,1,1,2	cctatctggctatagttcctgccgactttagcttagt	gaccagtgttatgactgctttaaacctctctctaaacgg	gcaaactcagctttggtctctttatgctgtagaaacct	acgggtaaaacgttatagtgcagaaggcattcctt	tttgaccctcaaggaaatgctagtgatgttcttgct
	72	RepB pheromone related transfer genes pPD1	E. faecalis	D78016	42-593	3	1,1,1	acagctctaacataatcagaaaccgtagcggcgta	cgggttacgaacatacttttagttgtattcgcttgaggg	cggaagtaaacgaacaagggaagatttacctacaccacc		
	73	hypothetical protein pHT beta	E. faecium	NC_007594 (AB183714)	3801-4655	5	2,2,2,2,1	tggagaagtactggttttactgatgaaggtgaagaagtct	tcctacagtttcagaatttacagataacgtttacgtggcg	ttcccacgcaaactgagcaagattcgattgacaac	tgacaacttgcacagtacggttactaattatcaaactgct	gaacgaggcgtaattaaacgttggggtagagaagg
	441	replication associated protein parA pAM373	E. faecalis	NC_002630 (AE002565)	3442-4275	4	2,1,1,2	aacatgcctattagactttgatatgcaagctgacgccacc	gctgacgccaccgatcttgtttttacaacaatggaat	agatgtaccacccacaataaatatctacaccgattctgca	tgttgccaacgagttaattactagaattggaaaggacgaa	
	444	putative ATPase parA Orf16 pRE25	E. faecalis	NC_008445 (X92945)	14258-15154	5	1,2,1,1,2	ggtggtgttggaaagtccaaattatcgactatgtttgct	tccctggcacgtttgattgatgttactgccaaaattaa	atcgtggcgagtgattacgttatgatccctttacaagc	acaatttacccctggactagatatgatcggttttgtccc	ttttgtcccttatttagttgatacggacagcgcaacga
	457	Antitoxin of Axe-Txe pRUM	E. faecium	NC_005000 (AF507977)	5213-5482	2	1,1	acctcgattaagtcatgtgttttaaatgcacctttggaga	acgtaaattttggcggaaatttgaataagctactgcttcc			
	458	Toxin of Axe-Txe pRUM	E. faecium	NC_005000 (AF507977)	4963-5220	4	2,2,2,2	aagataatcatcccaagcatcatcagaccaagccttaatc	tcatgctttaatggctcaggttttcctaatccagca	tccagcaaaggggggaacgatcgatatcttttattaacttg	tccttgctcatgccaataaagataatcatcccaagca	
	460	Putative zeta toxin pVEF2	E. faecium	AM410096	28627-29490	5	1,2,1,1,1	aacagacgttcctattcaaaccgcaacaatgcttca	agcttagaaacgccttccattagtccgaaagagacc	cggtatgaaactatgtatgcagatgatccaatgacagcca	cttaccgaccaatttagaaacccttcataaaacgggctta	gcatgacagaagcgatcataagccgtttgagagata
	461	Epsilon antitoxin pVEF3	E. faecium	NC_010980 (AM931300)	34542-34814	1	1	cgttcatgagtattggcggtcaatgaatcgttactcaaa				
	462	Zeta toxin pVEF3	E. faecium	NC_010980 (AM931300)	34816-35679	5	1,1,1,1,1	acagaagcacttattagccgtttaagcgatcaaggc	aggcacaggacgaacaacagacgttcctattcaaaca	atgtatgcagatgatccaatgacagctagggcaacac	cgtacgccatggcagtacccaaaattgaatcatact	tattcaagcttagaaacgccttccattagtccgaaagaga
	463	Putative transcriptional repressor Omega pVEF3	E. faecium	NC_010980 (AM931300)	12918-13133	3	1,1,2	atggcggaaacgtaaaagaagttatggatcaagcctta	atgggagataagacggttcgtgttcgtgctgactt	tgctgacttgcaccatatcataaaaatcgaaacagcaaag		
	464	Endoribonuclease MazF	E. faecalis	NZ_ACIX01000197	6108-6473	5	1,1,1,1,1	accaactcaaagagatattgttattatcgactttgcgcct	acctccgataatgcagtaagtaaagtgaatacgctcca	aggttatgaaatcagaaaacgacgacctgctctcg	cctcgttggataaggaacgtccttttcttgtccct	gctccaagtctattcactcgactatacagaacaggca
	465	Antitoxin MazE	E. faecalis	NZ_ACIX01000197	5873-6118	4	1,1,2,1	actgctgaaggagagttttatcttcctgaattagcgattg	attcacctagcggaggagaagttgatgggctataa	cttcgcaaaaataatggctccatcgcattgattcca	tcctgaattagcgattgattattcacctagcggagga	
	466	Toxin RelE	E. coli	NC_000913 (U00096)	1643370-16436	5	1,2,1,1,1	actttctcgtctataacctggtatacaaggcgatagcc	agaatgcgtttgaccgcctcgctatatacttccga	atcttgtaacaatcaggcataccacggagcttgtt	gctttcgccattcctttagtgcccgctcgtcaaaa	ttgcttcaatccggggtgactcaagtacttcaacc
	467	Bifunctional antitoxin RelB	E. coli	NC_000913 (U00096)	1643657-16438	5	1,1,1,1,1	agaacgcgctttaagttcatcgtcaatacgcaggt	catcactcaggagtgtctgtttgaacggcaagcgt	catccagcgtcacacgtactggcttaggattacga	cgaagccgttctttcactatctccacaagttcagc	cgttcattgtcagcgatatactcgagcatgagacg
	468	par toxin RNA I pAD1	E. faecalis	AD1REPABC	4103-4347	4	1,1,1,1	acacctccaatcgaggcgagctgccgcattaatta	actagttgcgacttctagcgtgtgtgttgatagca	cgtccaacacacgagaaatcatttccagaaccaatcc	gcagcttactttcggctatcgtcttcctcgtccaa	

Transposable elements	Conjugative transposons	199	ORF1+2 Tn916	E. faecalis	M37184	61-264, 346-15	5	1,1,1,1,1	aagtggcgaacgtcaagttcctatggttgaagaag	agcgagtgttagcgaatcgaaagaatgataagcgtg	gtggcaagtgattacaacggcatgatgaaaggtcttgt	ccacatagtttgcgacatacattctgtaccaactatgca	atgctaatatagccatgacgctgaactattacgcacatg
		213	Excisionase (xis-Tn) Tn916	E. faecalis DS16	U09422	16356-16559	1	1	ctatttgggaacgttataccctaaccattgaagaagcgt				
		75	Orf16 Transposon Tn916	E. faecalis	U09422	5193-7640	5	1,1,1,1,1	gccattcgtgcagtaaccaatagtgaagtacgagg	agtgattgaggaattacgggttgagaatacgccac	agccgaccatatcgaaagttttacagactatgactttgc	tacaggttgcggacttggtacttcccgacaaggaa	ggagcttttatccgttgctatgctgattgtcattagtacc
		202	Tyrosine integrase transposon Tn EfcTn1	E. faecium	DQ370176	638-1870	5	1,1,2,1,1	aagttcgtttgggtcatacggatgttcgaactacc	cggcagatcgttttgctgattacatgaataagttgcac	ttcgtagaaatccaatggaaggcgttattcgaccaa	gctttacgatggaaagacattgatttcaaacgaggaacc	agcatatgactgttcatggttttcgtcatacgcattgt
		450	Transposon EfcTn1 partial sequence	E. faecium	DQ370177S1	1-315	4	1,1,1,2	ctgataaatcagcgcttttcttattctgcagcgtttgt	attctgcagcgtttgtgtctttgagaccatactca	ctttgttcgttcactaacacactctgatgcgtccc	tgtactacttaaaagagagggtcaaaggatggtcaagga	
		204	Integrase Tn5386	E. faecium	DQ321786	28008-29207	5	1,1,2,1,2	aggaatgacaccgaataccttacagtatatcatgggaca	acgctgaattattacgctcacggctcatttggttc	tcgcagaaatacggtcaaaggcagacaacagttta	gaccctttcggaagtatgagcattgatagcgtcaa	tttgagggcttcgttctacatggcgatacaagatg
		208	Excisionase Tn5386	E. faecium	DQ321786	27721-27927	5	1,1,2,1,1	atcggtgcgaaatgggttattatgaacggcaaccg	tatgaacggcaaccgtattcagataaagcgaaaacagt	tgatgtgcctgtatgggaaaaatacacgcttacca	accattgaggaagcgtctaaatactttcgcattggc	cgtctggcagaagaaaatatcggtgcgaaatgggt
		74	hypothetical protein Tn5386	E. faecium	DQ321786	10955-12460_1	5	2,2,1,1,1	tttggcggtcaggcggtaatcttagacccgaaaag	tgtagaagacgctgaaagtctggcaatcgacatct	catgtgatagacgagctacgccgtgaagatacacc	atcgcagaccatatcgacagcttcacggactacga	aatcgtagaccttgatgaagcgtgggcgttcttaa
		231	responsible for insertion and excision of Tn5397 (T	C. difficile	AF333235	19045-20646	5	1,1,2,1,2	ggaaatgaaccaagattccgttgcagaacatacgc	gctaagtgaaacgagcggaagtcagaagattcatca	tgtctatgaactatgaaactgaacaagcagaggtagtgca	agaggagaaagtacagggtacggatagattccttgaaac	tgagctgattgattcaatttacgtgtatcaagcagaggg
		452	Integrase Tn1549	E. faecalis	AF192329	32432-33625	5	1,1,1,1,1	gctatgcgtcatacattctgcacccggttagccaa	agccttgcagtacattatgggacattccaatatcacc	cttagtgacgttctggacgacgatacggaaccaaa	gcatttcggaactgtgcggactgacggacaaagat	acagaacggctatcccatgacggcggtagattatg
		454	Excisionase Tn1549	E. faecalis	AF192329	32148-32348	4	1,1,1,2	gacatgcctatttgggaaaaatacacgctgacgattg	ctctggctgggtcattatgaatggcaaccgcatcca	cgcaagcagtttgagaaaataattgacactttggacaca	aaacaaattgcgtaagctggccgaggaaaatccgg	
		39	Rlx-like protein Tn1549	E. faecalis	AF192329	20529-21857	5	1,1,1,1,1	gattgtggacgtggcccttatcaatatgggtagtc	gcttgccgtcagttttgtccgggttctcgatgtag	gccatatccgcaagccggtgttccatagctttgag	aatccgggtaggtgtctatcccatgttcagtcagg	ggaaatgtacttgcgctgatttaccatatccacggc
		82	TrsE-like protein Tn1549	E. faecalis	AF192329	6228-8627	5	2,2,1,2,2	tccatgagctgtaaatccgagatcgtcagtgtgtt	tgaattattcggaggacgataacccgctggccctg	acaaagcgttgttagatcagcatgtcccggaggct	tgtgtccggctccctgaacgtatttaaccaccgta	ttgcggaacgcttgaatttgtccaccgaacagcag
	SSTE*	416	Transposase (tnpA) Tn554	S. aureus	X03216	134-1219	5	2,1,1,2,2	aaagttgctgtacgagttcatggaacagagaggtg	agcagacttcgtaggttggttgagatatccttcagc	ggtggtttaagaatcggtgaagtgttatcgcttaggc	ttcgccacactcacgcaacgcagctaattagggaa	aaagagattaggtcacgcacatgttcagacaacgt
	Tn3 family	145	Transposase Tn917	E. faecalis	M11180	2364-5282	5	2,1,1,1,1	tgataggcatacgactcatcatatcgaggttgcttca	gggaagctactcataccсttgatggcctactttatca	acgcacgtaagaatagagtagctcttgcactgaga	cagatagtgagctacggcgaaggatcactcatgga	gcaataagtatatggaacgccgtctacttacaagcagc
		146	Transposase Tn1546	E. faecium	M97297	75-3041	5	2,1,1,2,2	ttgaaagtcacggaatgcatacggctcgtatcttga	gtgaaggatgacccggtggctcttttaaccaaccc	tcaagcgaggatggactaacaccaatctgtttcgatat	ttttgatatgagtgtatggccaaccgggataccgaaga	tgttccggtgtaagcaattctctacctctcgcaatt
		214	Putative resolvase (resIP) pVEF2	E. faecium	AM410096	3130-3747	5	1,2,1,1,1	agcggtcaatcggtcgaacgcccacaattacaagc	tggctaaaattggttatgcacgtgtcagtagcaaaga	aattaaggggggcaaccctggaagtcttaaatttaccct	gatcggcaattacaagcgttacagggcgtttctaa	gaatcaatcgccgaacgtttagaaggtatcgagca
		216	Resolvase Tn1546 pVEF2	E. faecium	AM410096	15737-16312	5	1,1,1,1,2	gccaattcaatcccttcacgttgtctcatccgaataaga	agatctcgctctaattggttaacaccagccattacagt	aatcgtctaacactttttgaagttgctcgcgatcсttt	atatccattccgatctcgttcaactgctgaaattgtctt	ttgtcttgaaggattctggttagtcgaactgacacgaat
		223	Resolvase (Tn3 family) pEF1	E. faecium	DQ198088	9180-9752	5	1,1,1,1,1	agatgaaatagttgttacgaagcttgatcgatttgctcgt	cgaatggtgacacggacgttgttgagtgtagcaga	ggcacgcatatgaactgcttacttcaggtaaatcgt	cgaaatatggatatgcacgtgtctcaaccgcttct	aagttgaagctataaccggtttcagtcgcagtacg
		229	Resolvase Tn917	E. faecalis	M11180	1806-2360	4	1,1,2,2	accgcaattagaagaaatgatcaacctactacgtgaagga	acttcaacgtctatgggaagattctttttccgagttatgg	tccgagttatggctagtttagcagaactggaacggga	ggggccgtccaagtaaaggtaagctatcaattgattt	ttggctatgctcgagtgagtacggatgatcaaaatct
	IS256	15	IS256 transposase pTEF1	E. faecalis V583	AE016833	50710-51882	5	2,1,1,2,1	ttgtacggtatattgaaaggcgtcttcgaatccatcatc	aggtctcttcgctttcgccactttgaatcatgaag	cggtcgccatctttggttattccaatcgctatatgac	tgttctgttaagctagaaacgaaggacttagagacgga	gtgcctacacgtgtcgtaaagctgcgctcataata
		96	Transposase IS256 family pVEF1	E. faecium	AM296544	34341-35531	5	2,2,1,1,2	aggaagtgttgagctatgcgattgcaccaattgaa	cacaaagtgcgtgtcgatgatcgtaaggaagtctg	tcccattagccatacgcaagactatttactctacgaact	tgaagagtcgatggaacgtttcttagtctcgtcttttga	tgaccgttatgccgctatttatatggacgcaacct
		98	Putative transposase (tnp) gene IS16	E. faecalis	U35366.1	109-1296	5	1,2,1,1,1	aactgctaacggatggtgcgactgtttctaaatca	acgaggtttgttcggtgtaaaactggtcatttctga	ggtatcggcattgattctgatggacgacgtacgat	gttccacgaaccagagacggtaagtttagtcctact	tcaagacggcgaatcggaagataattgggataccg
		102	Putative transposase IS1542	E. faecium	AF114715	101-1273	5	2,2,2,1,1	gaactcgctcgaacagctaagaatgccttagtcgg	gcctgcgaaatattggataatggcttcgaagatgc	tgatgcccataaaggcctagtgtctgcgattcgta	tgctcgcttcaatgcttgagatgtatgtttcgggc	tgtttctagcctgactgagcagttagacccgatgg
		134	Transposase ISEf1	E. faecalis V583	AE016830	2701329-27025	5	2,2,2,1,1	tccatttctcaatcatgccggataagaaggtattcgct	ttgatggatagtatctttcatgccttttaagccgtccg	tggagttaatatcctgtagcagctcattccaaacgtatgt	acgtatgttgattcagttggagcaatcgcataactcag	gtcatgttggacatggtttgtggagtatagtgatgaccg
		154	Transposase IS256	E. faecium	AY971367	2711-3462	5	2,1,2,2,2	tccttgtacggtatattgaaaggcgtcttcgaatcca	aggattttctaatggcagagactaatcctttgtgcgc	tctcttcgctttcgccactttgaatcatgaagccg	tcgccatctttggttattccaatcgctatatgacagc	ttctgccactcgttaaccataggttctagctgttctg
		157	Transposase IS1310	E. hirae	AF406971	3627-4802	5	1,2,1,1,1	acggccaacttaacttaaccattcctcgagatcgc	aacgtcgactggcttcacaatatgcggctatcttc	gaagcggttcatattgcgattggcattcgtccaga	cgaaagaagtgctgaactaccaagtggcgccgacg	ccagccattcggcaatcactctactccactaactt
	IS110	104	Putative transposase Tn5382 ISEnfa110	E. faecalis	AF173641	362-1570	5	2,1,1,2,1	aactccagacaacttcttccgcgttagctgctctca	ccaatccggtcaaatagatgtccgtagccgtagca	ccacgacgcgggcctttatgtctcggtagtcaatg	ctggatatttatgcctctgcttgcagacacttcgg	tgatgagcgtcatcctgcaatgtgctccaatagac
		165	transposase, IS116/IS110/IS902 family	E. faecalis V583	AE016830	1829566-18308	5	1,1,1,1,2	caactccgtaaggagatacgagcctgagtaccatg	acacaatggaccaacttgccgaactgtcattagaa	aggcaatttctaaagccatacgtagttcctaccggt	cgggtatactcgctgaaattggccctatcaatcgc	tggcgacagaaccaatcaggtaactctgagtatga
		167	Transposase IS111A/IS1328/IS1533; transposase	E. faecium DO	NZ_AAAK03000010	2042-3292	5	1,1,1,1,1	gctcaacacacttgtaacgattgaaaatcсttttcggg	aatacatggccttacagcgtttaacacgtactcgc	agtgcaaccctcatttcactcatgacggaagattt	gcaattaccatgagttaccgcctaggtgctcttgc	cgttggtaaagtctatgctgccggtttaatcgctg
	IS3	109	Putative transposase ISEnfa3	E. faecium	AF469486	755-1393	1	1	ctgagggttcactgattctacattcagatcaaggatggca				
		110	Transposase IS1485	E. faecium	AF029727	76-330_330-12	5	2,1,2,1,1	agaacttgaagaagtagtcgctgaagaatttatccgcag	agagacaacccattagaagcgatcgtaacagacttgact	gattggcgcagcgaatccttgataatgagctcgga	tgcgaataagcatctttaccctatatcagcgatgtgc	tgtcaattggtggaaccaccttcggttgcacggta
		143	Transposase IS981 pEF1	E. faecium	DQ198088	1-1101	5	2,1,2,2,1	tgcttcagataagccatattctttcgctaaccctcga	cgctaaccctcgaacagaacgtccagtttgattca	tgttccacttctcttgctgtgtattggctgcccaa	tgcccaagtcagtgtgtagaatcattccttctgga	aggaatgtacgtaatgtcagctgcccatttctcac
		162	Transposase IS3-like	E. faecium	AY916786	1250-2191	5	1,1,1,1,1	gtgggatttacggctaccgaagaatgaccatcttt	agcgaaagtgaatcataagtgtgtacaccgcctca	gggacaaaacgttacctgagcgctatttatgacct	ccagtcaccgcaatgacaacccgttagtactggat	gtcacgcgtctcgaaatgtattgacaacggcccta
	IS6	111	Transposase IS6 family pTEF1	E. faecalis V583	AE016833	12615-13304	5	1,1,1,1,2	actttgacataggtctcatcaatccgccaggattttgat	atctgttacaatggaacgtggttgaccgtagactctg	ccacatatctaacgtattgcccttcgaatcaatggct	cgttactgcagctgtataggtggactgaaagttgtt	tggtatgatccacgccaacactaaatttacgcatgat
		112	Transposase IS1216 pVEF1	E. faecium	AM296544	36849-37535	5	1,1,1,1,1	aatagaggggggctttatctgtgactacaaccttcggtt	cgatagctaaggttataacgaagatagtagcccacggc	ctttaatcgtggtagaggcagtgcgtaaacttcgatag	gatttcagtacagaccgaaaacccgaagagagtgcctt	tacaaccttcggttcatcaaactgcttcactaaccgct
		156	Transposase ISEnta1	E. faecalis	AY884205	509-746	4	1,2,2,2	gccgaaaagaagggtctctttttggcttttccgtc	tctttttggcttttccgtctgtacagaaatcaaaggact	tctgattgaacaagaccatcgtccaattaagcgaagaa	tttaccaaagtttgcgaacagcctcaaccacgatt	
	ISL3	117	Transposase IS1251	E. faecium	L34675	128-1417	5	1,2,1,1,1	cccgaaggctatcagacgaccttgagaacgttcca	ttcaaattgcgcatcataattcgtcatggcaacgc	gcggtcgaatggatcgtgattgatatgtacgctcc	accggtttcacatcgtccaacatattggtcgtacc	gaacgcaagcttgtatactcgagcctttggcgacc
		118	Transposase ISL3 family pPPM1000	E. faecium	AY351675	8074-9396	5	1,1,1,1,2	aggctcactaacgcttatacggagtcaattaacagca	gcaggtagagcgaatgggtagaggttactcgtttg	acctcggcttgtattgactaatattgaacgcaggac	aagccctacaaagacgcagtgaacactatccttcc	aaagacctcgtgagagccgtagacaactggcatgt
		119	Transposase ISEfa11	E. faecium	JN208887	3241-4536	5	2,2,1,1,2	tccttattggcagaacttcccgaaacgttagatgaca	tctgacctatgaagaaggcatcaccaacgcaatgatc	ctgccaaatgcgaaagtcgtgattgatcggtttcatatt	acacatgaatcaagcgttcaatgagttgcgtatccgt	ttccgagcaccaaaatacccatttcttactgaagcaa
		120	Transposase ISL3-like	E. faecium	AF403298	1836-3176	5	1,2,1,1,2	tcaacactcccattacaaacgcttatacggaatccc	tcccaaattggtcacaaactggttatgaatgggtgt	gacggtacgaaatatcttcaatgactatgttgccgaact	agtgctgttaatacagtgataccacatgccaaaatcgt	tgagagatcgttacgtgcttcttacaaggagtaaagact
		123	Putative transposase IS1476	E. faecium	U63997	140-1414	5	1,1,1,1,1	cacgatcttccatcgctaccaatcgggtatttcaa	agcaacgaagcattaccagtcacgtatgaacttcc	acgtgtccagatcatgaatcgttttcgaacctcac	gaagaagtaccgtcggctcaagcgatattggaaac	gctgttaagctacaacgaagaattgcgaactgcgt
		176	Transposase IS1167	E. faecium DO	NZ_AAAK03000135	3172-3764	5	2,2,2,2,2	tggtccgtttgaatagctgtagtgaagcgcatttt	tctggcagaccgctaatgtctctagttaaacaatcct	tggcgtaagttgggacaagcagctaacagtcgatc	tctcactttcagtcaaataatctttaaaactggcacgcca	tcgatgattcaagaaagttctgccaatatgttggacga
	IS982	129	Transposon ISEfm1	E. faecium	AF138282	297-1206	5	1,1,2,1,1	gcaacgcgatacagtgattattgcttgtgttatatggggc	cctaatggtgactttgctagtaggagtcgattcactcg	ttccgattacttactcaatcacgaatccaggtgtccat	aggtgtccatgacgtgaaagtgttagagactttatcggaa	tactggcttacagtgttctattaagtcgagcacaacga
	IS1182	138	Transposase IS1182 pUW786	E. faecium	AF516335	12234-12908	5	2,2,1,1,1	tctttgtgcctatattcaaggggtttattcggcc	tgctgatagatagtattcggatgcgctgcttatctcaaga	caaagtggcttaatcagtggcagtgctctatacattga	gcgattgaccaaaccgaaacagtagaagggggctg	gctcatgccattgtgcagtttgttgactctattccc
	IS200/IS605	140	Transposase-like protein A ISEfa4 (similar to putat	E. faecium	AY082011	900-1304	4	2,2,2,1	tgacaaaattcatcaatgcctataaaagtgccagctcaag	tgccagctcaagattgataaaacgtgactttccaaga	aaaagtttttgtcttctgacaactggtggcgcacc	acaactggtggcgcaccaattgatgtgattaagaa	
		142	Putative transposase ISEnfa200	E. faecalis	AF125554	296-766	5	1,1,1,2,1	gccaatatcctaagtatgttatgcaagaggaaaggcgt	agcagagatatgtccagatcacgtccatatgctgg	ggaaaagtacactaatgatattcgagcggcatgcca	tctggtgcagaggatactacgtagatacggtaggg	agccaatcagctacaggaagatctggaatatgatcaaatg
		166	Transposase IS605	E. faecium DO	NZ_AAAK03000014	6433-7581	5	2,2,1,1,1	tggcaagaaatctcttgaaattcgagaatggacttgtcc	tggacttgtcctgtttgtcatactcatcatgatcgtga	accgaaggtctaagactacattctatgggattggct	agaaattgatagccttgctttagccaatgcccaact	tcagatttgttcagagtgcggacataaagatggca
		170	Transposase IS200 family	E. faecalis V583	AE016830	575151-57554	5	2,1,1,1,2	tgaagcgcatgcaatgcccgaccatatccatatgtt	ccaccgaagttgagtgtatcaggttttatgggtttct	ggaagaagtgcagtaatcattcatgaacgtcatgcga	ggtcaaaaggttattatgtgagcactgtaggactgaacc	tgtgtgagatgaaagaagtcgaaatcattgaagcgca
	IS1380	126	Transposase ISEcp1-like E. casseliflavus plasmid	E. casseliflavus	AY939911	561-1823	5	2,1,2,1,1	tttgggcaaatgaagcggtgtttcaaatgatgatgc	agtgtaatcaacgtagaaggtcatcaagaaggtgcatca	aagaaactaggaaaccgatgctacaatatccagtttgca	gtggcaatacttacacagctaacggtgcatctgaa	aattcatcggttgtattcgttaaaggtgaagaaggcaga
		137	Putative transposase IS1678	E. faecium	AY859490	156-1475	5	1,1,1,1,1	gggaaattagtccaaacgggtagacgagtctatctca	ccaagaacttatcgataaagcacgcctcatccgtaa	catccattggtagcttttgacggattgactggtga	cactgacatcttggttcgtggagacagcgggttcg	agtacttctcgaccacttatcaagcacaatcctggt
	IS30	130	Transposase IS1252 IS6770 homolog pHKK701	E. faecium	L38972	665-1624	5	2,1,1,2,1	tcattcagtgctcgttgcgttggagtccctggatc	accagtggttcagagtattctcaatatcgatcgcct	gaactggcgataaagggttcttacggaacagctga	tctttatactgttggtagtattcgagggccgtatggc	aatcggtgttcgagatcgtttcaggtactcagcaa
		133	Putative transposase IS6770	E. faecalis	L28754	97-1056	5	1,1,1,1,2	gttgaaatcgctaaccgaatgggtcgtgctataca	agaaaggcctgtttcatgcggtatgagaacacttt	attatcacgattaaacccaacggccgtaaggcatt	ccctggaacaccttctcaacgcccattaaacgaga	tctgcgtcgtaatggactgccgaaatcaatggatt
		136	Transposase IS1062 plasmid	E. faecalis	X96976	2496-3455	5	2,1,2,1,1	tcatttaaacctctttgtgatggtgtaccaggatctgc	atgcttaggtacagattgcatccattggtcaaacga	tcctttcataggtaggtgagtcacttcaaatacgcct	gtttgttcatctgataagacaataggacgtctgccaca	cgcctctatcattgcaagttcatttgatgtaagatgggt
	IS4	168	Transposase IS4	E. faecium DO	NZ_AAAK03000061	5880-7001	5	1,1,1,1,1	aattcaatctgccaacgggcttgatacatgtcggc	ctgattatcgtcccatcacgggttgttgtatctgc	gtaatcaaagtagccgcgatccaccacatacgttg	acgagataacgagctgtaacacagggaatcaatgc	cgcgatagcttggcaattcttcataaaccgcatga
	IS21	160	Transposase IS21 family	E. faecium	AB105542	105-1343	5	1,1,2,2,1	aaggatttgacgggagctacagcctagtaaaagct	agcataacccgggactttccgcccaagttgattgg	tgaagacggtggttgatcattctcgcagtcagttt	ttcagtaaagacgccagcttcacgccaattgcctg	aggtactctgtagatcctcgctatatcggtaaagaggt
		417	Transposon IS21-558 (integrase/resolvase B, 23S	S. aureus	EU598691	1..3006	5	1,2,1,2,1	aaccattcatcagccgatttgcgacaggttatgct	aaatgattacagcagcaatcggtctcgacaacggc	aacagcagtacaaacgatcgtgccagatctgagag	gctttagggattccgttggcagcgaaaactggtac	tcagcaaccaaacgagcatcggaactattgcaata
	Various targets associated with transposable elements	144	Transposase ISEfm3	E. faecium	AY887084	111-1019	4	1,1,2,1	tactagccaaagagccacgtatagagcggtttgttacg	taatggtgactttcctagtaggagtcgattcactcgct	tttccgattacttactcaatcacgaatccaggtgtcca	caggtgtccatgacgtgaaagtgttagagactttatcg	
		147	Transposase	E. faecium	AJ223633	3011-3301	3	1,2,1	agattgctttacccatttgtcaaatgctgaagccgtta	tccgaatatcagcgctgcttgctttaaaatatcattttcc	cgtggctttccagctaagtaaagattgacgatttgttg		
		149	Transposase pEF418	E. faecalis	AF408195	12655-13092	5	1,1,1,2,1	tcaatatcaatcgcttgtctaccacacggtttcaatgt	tatatttgaagctgaattcgcgcgaccggatcagc	caccttcaaggtgaccaaactcatttgagaattggc	ttcacgttcatgaatagagcgacggaaagcttgtt	ggtttacgcttgcctttcatcggtaagtcagaaggatta
		152	Tranposase pAM373	E. faecalis	AE002565	5363-8335	5	1,1,1,2,1	tcagctgatggtattcgttatactactccacaacgttct	cgagctaactcctaatcagataatgacagatacagctggt	ccttattaggattccaatttagcccgcgaatcgca	tggcgaattgattctgatgcggattatcacgaact	tcagcttcgctatatttctgacgaaatttatgcacgaca
		153	Transposase Efm DO ctg590	E. faecium	NZ_AAAK03000049	1552-2133	5	2,1,1,1,1	aaatctacatgacagccgaatgctcgtgccacaaa	cgtgccacaaatcgaacgtgttaaatcatgttgtgg	ggaacgccaagtatttaatcgatcgaaaactacgaccc	tcaaacacttcagtttcgcacaactactcgagatgg	ggtggtagaagggaaaattagtgtcgcagatccag
		163	Transposase	E. faecium	AJ223633	2127-2975	5	2,1,1,1,1	tgtaccgtgcaaccgaagtggttccaccaattgac	ctactttcagcgacttataagttgattcagctacggcatt	agcacatccgagctcattatcaaggattcgctgcg	acagttaaagttagactgcctcgctagcgttagca	gtcaagtctgttacgatcgcttctaatgggttgtctc
		171	Transposase	E. faecium DO	NZ_AAAK03000155	1-218	4	2,1,2,1	tgaaatggagaatgacattttaaagcaagcggcgc	cattcgagattatgagctgactacctcggcctttg	ttgataagtgggtcaagcaagcgcaaactactggc	agcgcaaactactggctcgtttcaggaaaaagata	
		173	Transposase mutator type	E. faecium DO	NZ_AAAK03000050	3310-4500	5	2,1,2,1,2	tccaactctgaacgaaacagttcggttacagcctg	agatagtcattgttgctgagacttttcaccattgatggat	tctttgaattttagagcccgggaaaatgtcctgtattaca	tcagcggactcgtttggtgcaatcatatagccaag	ttccctggatacagtatcacggcgtaaaggcatgg
		177	Transposase	E. faecium DO	NZ_AAAK03000019	25202-25645	5	2,1,1,1,1	tgtggtagacgtccgcttgttttacccgaggaaca	gttgttcaaggatggacacccgatgtgattgttgg	ggtcgtgcagcgtttcctattagttgttctgctcg	cttctgacttaccgatgaaaggcaagcgtaaaaccgaa	tcaatctgttgctaaaactgcccattgcttgaatcg
		179	Transposase	E. faecium DO	NZ_AAAK03000007	51725-53149	5	1,1,1,1,2	accgcgtgccatgcattactctcgctattggaagt	gacatgtttagtgcctcgactactaccgatgcgta	tcaggtggcgttcctcggaaaatacggattgataa	gactgaacaagcggtgctgactgacggtttcctcc	tgtgcaatccacgtagcggaaacgagaaaggtagt
		181	Transposase of IS981J	E. faecium DO	NZ_AAAK03000010	53018-53332	1	2	tgtagcctatccatgatcggtttattagacctttgaggt				
		188	Transposase ISEfm2	E. faecium E59	AY887085	95-1273	5	1,1,1,2,2	cggacgaatgataccttagaggagaccgtcattcacc	accatgtcggaaatcgcagacttgattgagaaaatgta	cgacctacgtttggaatgagttgttacaggacatttact	tgtgttcacgtttcccgtaatattgctcataaagtacgt	ttccgcctggtatccgccgaagcatttactcaact
		196	Transposase mutator type	E. faecium DO	AAAK03000007	59056-59637	4	2,1,1,1	tcgcactggttttaattcaggtaattcgagaaacggttct	acggttcttactttcgatcaatcaaaaccgaatatggtga	ggtcatcactatactccacaaaccatgtccaacatgacta	gcgatttatattgccatggtatacgagaagacggca	
		422	ORF480 potential transposase Tn552	S. aureus	X52734	894-2336	5	2,1,1,1,1	tcctcaagacccaatgaaatatggcaagccgacca	accattattatggatgactatagtcgtgcaattgcaggc	cagatcacgggagtgactttacctcgcaccacatg	atgttttcaaaagttggtgtccctcgtggtcgcggg	gctatccagagcactcctatcaatcggtggaacag
		424	BinL/TnpR Tn552	S. aureus	X52734	2308-2901	5	1,1,2,1,1	gttgtttggagactcgacagactaggacgtaatatgga	cgaattgcagcacgtgctagaggacgctatggagg	tgaatttacaggaggaccgattaaacgcatatggttctg	atgataatggcacgccaattaagacaattgcagagc	agcagtggcaagtttcacgtacaacgatttatcgt
		206	Integrase/recombinase XerD, putative	E. faecalis V583	NC_004668	1492090-14929	5	2,1,1,2,1	aacgcctgatacaagtaaaccactaggtatgcgga	gatgtatgcaacgggcctgcgagttagtgaactaa	tcaatggattgagcgctatatgaacgaagccagac	tcatcatggcgaaccgttatctagacagggcattt	gcatacattgcggcatagctttgcgacacatttgt
		212	Excisionase putative PAI	E. faecalis V583	NC_004668	293125-293397	5	2,1,1,1,1	tttagagaaatggctggctttaatgcaggacacgc	gacacgccgcaattttcgtctggtgtaattcatcc	gtttgatgagtttgttcgctggatggataaaaaccgtt	ctggaagctgtcgaacagaatccagatcgtgcgtc	caaaaccggttaaggttgatctggaagctgtcgaa
		215	Resolvase (res beta) pAMbeta-1	E. faecalis	AF007787	5145-5762	5	1,2,2,2,1	atcggtcgaacgcccacaattacaagctatgctta	tgactaaagtagggtatgcacgtgtcagtagcaaaga	ttgtataagtaccaagcggaatctgaacgcaaacga	tggaattaatcgccgaacgtttagaaggtatcgatcaag	acaactgaaagcgttagagggcgtttctaaggtct
		219	Resolvase pLW043	S. aureus	AE017171	47565-48122	5	2,1,2,1,2	tggccaatattggttatgctcgtgtatctaccagaga	aggcaggactactaagcaactgatagatttagccca	tgagtgcttttgctgaattagaggctaacttattaagcga	ggtttagaatcagctagagcaagaggtaacatgggagg	ttggttgtgagaaaatatttagcgaaaaagttagtgggcg
		227	Resolvase pK214	L. lactis	X92946	21-593	3	1,1,1	agaatccttcgcaagaaatcctagagaagcattagcaac	atgacacacaagatgattgaacgaaaaactggcattagtg	agaaaaatttactggaacaacaaatagtcgcccagcct		
		224	Recombinase (resolvase family) pTEF1	E. faecalis V583	AE016833	59210-59806	5	1,1,1,1,1	gtgacgaaaattgatcggctagctagaagtatcgttgatt	atgcaaacgttaatgatgaatatgctgggtagctttgc	tatgctgggtagctttgcgcaattcgaaagagatttaat	gggaaacagtggcatcgagcgaacaataaaaattatcg	acagcatgcgagaagtggaaaggaaaacaggtatt
		220	Recombinase (phage integrase family) pPER272	B. cereus	DQ889678	171299-171991	5	2,1,1,1,1	aaagtcgagatcattacggcggcacatgtacaaat	gcgttgaagtcggtctttaagcgattgaatgagca	gacctatgcaggctatagagattactgcgccatgc	atgttgcactgcgggttgcgtgtaaacgaagtaaa	agaaccgtaaaactagagctgtacctatgacgaagaagg
		376	E_faecium_DO_contig655_putative_recombinase_	E. faecium		5674-4301	5	1,1,1,1,2	ggcgtctatcaagccttgattgcccaattgacgca	acaattgttatgtgaccatgtttgtcggatcgaggt	acaacggcaatacctatcttctgcggttccatatg	ccttctgtggtcgtcatctgagttcgaagtatcaatgg	tcttcgggcagaaagggttgagtcagaagtattgg
		378	E_faecium_DO_putative_recombinase_ccrB_like	E. faecium		4297-2660	5	1,2,1,1,1	catgccaattcgattcgtgccgagcaagcggaaac	tgtttccgatgttaaaagaccgcttagacgaattgact	aggagagccgattcaagtcaaagatgtccaacgga	agatattggagattacaatgaaattcacgcctgccgt	gcagggtatttagaatatgcgcgctacatcaattggg
		425	Recombinase (sin) pSK1	S. aureus	GU565967.1	19661-20269	5	1,1,1,1,2	agcactcaattttgtcagaatgggagatcgattcgttg	gggagatcgattcgttgtggaatctattgatcgtttagg	acccacagaaacgtgttatttatcatcgcgtggtg	atcatcgcgtggtggaaatgttagaagaaggcaaag	tttggtgtggagaaaatctttacagagaaacagtcggg
		423	Putative invertase (entiv) Tn5384	E. faecalis	U39859	1275-1883	5	1,1,1,1,1	agaagcacttaactttgtgagaatgggagatcgatttgt	gggagatcgatttgttgtagagtctattgaccgtttaggt	agaacgaaatgaaagtaaacgtagacaagcacaaggga	ggacggccattactatattcacctaatgcgaaagatcc	cagagaagcaatcaggcaaatcagttgaaaatagacctgt
		40	MocB-like protein	E. faecalis	AY271782	4020-4349	5	1,1,1,1,1	cagagttctataaaatgatgcgtgagctgtccgcc	gtccgccattggaaaccgtatcaatcagcttgcgg	gctccgtgaggaagcaagtaagtggcatgagtttc	gattgacattcgcaaaaggtatctgcttccacgca	gccgaataccttaatgagcttgtgcggaaatcggg
		418	Putative genes Tn5406	S. aureus	AF411128S1	1-1164	5	1,1,1,1,1	gcaatactcagtaacgccaacagtattatgctcggt	acgcattacctaagaaagtctacgtcattcgagaagatga	aatcatcacaatgtatccaaattatacgggccttcttcgt	gcccgttgttgaatcacgcgtcaatgaagtattaga	gaggatgcggcagaaatcttgcgttcattcattgg

Resistance traits	Glyco-peptides	237	vanA Tn1546	E. faecium	M97297	6979-8010	5	1,1,1,1,1	cgagccgttatacattggaattacgaaatctggtgtatgg	gggaaaacgacaattgctattcagctgtactctcgccg	gcatgacgtatcggtaaaatctgcaatagagatagccg	acggccgcattgtactgaacgaagtcaatactctg	gcaggtattgcacttcccgaactgattgaccgctt
		238	vanB (D-alanine:D-lactate ligase) Tn1549-like	E. faecium	AY655721	4857-5885	5	1,1,1,1,2	gagccacggtatcttccgcatccatcaggaaaacg	attacagttcccgcagacattccggtcgaggaacg	ggatggcggcatcgttctaaatgaggtcaatacca	gttttacatcgtacagccgctacccacgtatggtg	tgcactgattgacagcctgattacattggcgttaa
		280	vanE D-alanine,D-serine ligase	E. faecalis	FJ872411.1	39736-40794	5	1,1,1,2,1	agggatcaccgaagaaggtcattggtatctatttgaagg	ccagttttacatggaggttatggtgagaatggtgct	tatgtaggttgtggtatcggagctgcagcaatctc	ttgctgaagcaattggtgtaaaaagcacccctagt	cctggttttacagagcattcgagatttccaatgatgatga
		281	vanG D-Ala:D-Ser ligase	E. faecalis	AY271782	21049-22098	5	1,1,1,2,1	ctatgccctgttgtcgtttcccaaaatcgttccgt	aggaatacctgttgttggctgcgatacactctcat	agggcacataaactcgttagccttgcgggtatatc	tggcatagatgagctcattgttggcagagttgatga	aataccaggctttacctcgcacagtcgctatccaa
		284	vanG2 D-alanine,D-serine ligase	E. faecalis	FJ872410.1	39328-40377	5	1,1,1,1,1	atttcctgtattgcacggcaagaacggcgaggacg	aggcacataaacttgttagccttgcgggtatctct	caattagcgtttgaacacgatgcggaagtcattgt	gcggtacttggtatagatgaactgattgtcggcag	ccaggctttacctcgcacagtcgctatccaaatat
		313	vanL D-alanine-D-serine ligase	E. faecalis	EU250284	955-2004	5	2,1,1,1,1	tggggtgacgccttcggctatttgtatggacaaat	aatacttattgcatgagtttgctcagagtgtgggtgt	gtgcccctacgctcataattcgcactagaaactgc	aggttactaggctgttcaggactagcacgaattga	cctggttttactgagcattccagatatcccaaaatgatgg
	Amino-glycosides	247	Streptothricin acetyltransferase (sat4) Streptothric	E. faecium	AF330699	927-1469	5	2,1,1,2,1	ttggaaccggtacgcttatatagaagatatcgccgtat	gaaagatatcgataaacccagcgaaccatttgaggtga	ggcagcgcgcttatcaatatatctatagaatgggcaaa	tctatcataattgtggtttcaaaatcggctccgtcgat	cgagaattggacctttacagaattactctatgaagcgcc
		251	aminoglycoside phosphotransferase aph(2')-Ib ger	E. faecium	AF207840	122-1021	5	1,1,1,1,1	actgtaagctatcttatcaaatccctgcggtagtgtatca	aggaaaaggatgcccttgcatatgatgaagcgacg	ggagcagctgttaactgatgagatgttggaacatatcg	gtgcttgctggattgtagtacagatgatttcggga	caggaaggtattaaaatactatcagcataaggcgccgg
		252	Aminoglycoside modifying enzyme (aph(2'')-Id)	E. casseliflavus	AF016483	131-1036	5	2,2,1,1,1	ttcgaccaggtagaaaaggcaatagagcagttatatcct	tgccatcagaaacgtaccaaatgtctttcgcaggt	attgacacctcttctactcaataatctgccgaagcaatc	ggcagctaaggacctggcccgatttctaagtgaac	taatcgattttggagatgcagctatttctgatcccgacaa
		253	Gentamicin resistance protein	E. gallinarum	U51479	196-1116	5	2,2,1,2,1	tggaaatcccttcgtgggctaccgtaaagtccaag	tcccagatataagcatacaatccgtcgagtcgctt	agtctttaagggactatctcacgctgcgcttccaa	tgactcatccggtatatacacgatatacgccgagact	cgatcaagtcagttacctgttagaaggcttaagggc
		254	Aminoglycoside(6) N-acetyltransferase aac(6')-Ii	E. faecium	L12710	169-717	5	1,1,1,1,1	gaaatgatgaatccagaacgaatcgcggtagcagc	acggtatcacaggttgggaattgcatccattagtt	agctcccgacgaaagaaccaaataggtactcgatt	aggtacggatgatttagaccatggaacaacgttaagtca	ctttctgatttactgagactgacttggccggaagaatatg
		256	Aminoglycoside-3-adenylyltransferase (aadA) Stre	E. faecalis	AF052459	107-898	5	1,2,1,1,1	acaacgcggcgagctttgatcaacgaccttttgga	ttctccgcgctgtagaagtcaccattgttgtgcac	cgtggcgttatccagctaagcgcgaactgcaattt	gccacgatcgacattgatctggctatcttgctgac	gcgaaatgtagtgcttacgttgtcccgcatttggt
		258	Spectinomycin adenyltransferase (aad9) Spectinoı	E. faecalis	M69221	271-1038	5	2,2,1,2,2	ttcagcaagaaatggtaccgtggaatcatcctccc	ttttctgatgtgagaagagccattatggattcgtcagagg	atggacacgggtaaaatcataccaaaagatattgcggg	tgcagtggctgaatcttctccattagaacatagga	tggtacttacatgtttggatcaggagttgagagtgga
		263	Streptomycin-spectinomycin 3' adenyltransferase a	S. aureus	AB253625	1-975	5	1,1,1,2,2	gcttgatgaaacgacgcggcgagcattgctcaatg	ccggcgatgagcgaaatgtagtgcttacgttgtcc	gacatcatgagggtagcggtgaccatcgaaatttc	tgcccagtatcagcccgtcttacttgaagctaagc	aaatgatgtctaacaattcgttcaagccgaccgcg
		381	aph(3)-IIIa aminoglycoside phosphotransferase typ	E. faecalis	NC_008445	39505-40299	5	1,1,1,1,1	cacctatgatgtggaacgggaaaaggacatgatgc	ggcgtcctttgctcggaagagtatgaagatgaaca	ccctatacgaatagcttagacagccgcttagccga	ctgaataacgatctggccgatgtggattgcgaaaa	acaagtggtatgacattgccttctgcgtccggtcg
		382	aminoglycoside adenyltransferase aadD Kanamyci	S. aureus	AB037420	438-1199	5	2,1,1,1,2	tctcttggtcgtcagactgatgggccctattcgga	agcccaaacgttccacgatgcgatttgtgccctta	gcaaatggcgtaatattcgtgtgcaaggaccgaca	atctgttatacgacgagcgcttcggtcttaactga	aacgacacggatatatagtggatgtgtcaaaacgcatacc
		383	ant(6)-Ia or aadE aminoglycoside 6-adenylyltranse	E. faecalis	NC_008445 (X92945.2)	37965-38873	5	1,1,1,2,1	taatgatgcaaaagccggaggatatggaattattcccac	caagcgcaagggagtatgatgattgctgcaatgaa	tatcatggaaggtcggcatcgaaacaggctttaaa	tctatgccatcaattgttcagggcggtatccggtg	ggctgcctggatagcacatatgccgctgatataga
		384	Spectinomycin adenyltransferase (spc) Spectinom	S. sciuri	NC_005076	10755-11537	5	1,1,1,2,1	acagcgatgtagatgttctagtcgtcgtgaatcatagttt	gttacggttataaataggagtgaagttgtcccttggcaat	tacttgtctccgtacctttgacagatattcgaagagcaat	ttttaaccctagctcgaatgtggcaaacagtgact	gaaattacctcgaaagatgtcgctgcagaatgggc
		430	Streptomycin adenyltransferase str	E. casseliflavus	AY939911	7990-8724	1	2	tgctgtaatgagttttggagtgtctcaacgtatgtagc				
	β-lactamases	420	blaZ beta lactamase Tn4002	S. aureus	X16471	142-987	3	1,2,1	gacacttcaacacctgctgctttcggtaagactttaaata	tgatgttgcttttgtttatcctaagggccaatctgaacct	agtgataagccaaatgataagttgataagtgaaaccgcca		
		473	Low affinity penicillin binding protein pbp5	E. faecium	X84860	523-2559	5	2,1,1,1,2	aacaaccattcatcagccgatttgcgacaggttat	atcacagcagcaatcggtctcgacaacggcactatc	cttgctagctgatactggatatggacaaggcgaac	gcgaaacagcagtacaaacgatcgtgccagatctg	ttctgctttagggattccattggcagcgaaaactg
	Tetracyclines	242	Tetracycline resistance (tetL) efflux pump	E. faecium	AY081910	21-1397	5	1,1,2,2,2	caggaatcattgtcattagttggctggttaccttgaatgt	agcatttggtcttattggatcgatagtagccatggga	tggaacagtagcagggtttgtctctatggttcctt	aaagatgttcaccagctaagtactgccgaaatcgg	tcggctacattggtgggatacttgttgatagaagagg
		243	Tetracycline resistance (tetM)	E. faecalis	X92947	214-2133	5	2,1,1,2,1	tccgcttctacgatattacgtggattctacgacacatg	agattttcggatgcttgctcctattgtattggaacaagt	cgatgctcctaaatattgtgcgaacatcgtagacactc	tcttagtggagaaatccctgctcggtgtattcaagaata	gagttaaaagggtaccatgttactaccggtgaacctgt
		262	Tetracycline efflux protein tet(K) pT181	S. aureus	S67449	1-1380	5	1,1,1,1,1	gtctttctgaagaagaagttgcttctggaatgagtttgct	agggaacaggtatagcaattgtaggaggtttattgtcact	aggatcaattgtagctttaggtgaagggttaggtcct	aagggttaggtccttcaataggggaataatagcacat	gggaaaaacattccgtttatgcttggtttgttttctggt
		385	tetO tetracycline resistance	E. faecalis	AY660532	14-1933	5	1,1,1,1,1	gctccctattggaagcggagtgcagtatgaaagca	gcagggacagaactattagagccatatctccactttga	gcaggaatatctctcacgggcgtatcatgatgctc	ggagtctgcttgacagagttaaaaggataccagcc	ccgtatagataaggttcggcatatgttccacaagttagc
		386	tetS tetracyclin resistance	E. faecium	DQ295784	146-2086	5	1,1,1,2,1	aggtgaggtccaaatggaggtaacttgtactctga	ggtatacgctatgggtgtgaacaaggattgtacggttg	gatttccgaatgcttgcgcctattgtactagagca	ttccagcccgttgtattcaagagtatcgaaacagttt	actaacattaagtctgctttccaaccacgtcgtcc
	MLS	233	msrC protein putative	E. faecalis V583	AE016830	1392319-13937	5	2,2,1,1,2	tctcccaaagtccggttaggaacgtatcatcaattaga	tgaagcggacaaattattctgaaaagattgtgcgttcac	agtgcctatcaaggaacagtaattttcacttcgcatga	taatgccagaattgacagattacccgatgttgcgc	tcgcttatgagaatcgaacaattcttaaccaagtgaacgt
		234	Lincosamide nucleotidyltransferase (linB)	E. faecium	AF110130	127-930	5	2,1,1,2,1	tcatccaactggttgtttgacgtagctccgtactt	cctgatacgaaggctatgcttatttacgatgaaacaggg	aagtggtgcaagaccaaatagacttactgaagaaaatgct	tgggaatcaacgttctaaaaagaggagaatatgctcgttc	cagcttgtatgatgtatggatcgttaccaaaggagaagg
		240	Hydrolase VgB (inactivates streptogramin B family	S. aureus	AF117258	2869-3768	4	2,1,2,1	tccagcagtaattgcatgaggtcgagcgtttggcg	accaactggtcctgatgcaggtgtaggaattttaaact	tacgtccaatacggttgccattcatttctgtaaaccat	taccgtagggtgctgaatctgggttaggcaatgta	gacttttgcatctggtgtcggtagtgggtactctg
		260	ermA S-adenosyl-methionine dependent methylase	S. aureus	X03216	4551-5282	5	1,1,2,1,1	gtcgttcaagaacaatcaatacagagtctacacttggctt	ctccaccattaatagtaaacccaaagctcgttgcagattt	tgcaatcttttcgcaaatcccttctcaacgataagatagc	accgcttctttagtcacttgacataagcctccatcaatt	taatattagtgacatttgcatgcttcaaagcctgtcgga
		298	erm(2) pBT233	S. pyogenes	X64695	8009-8746	5	1,2,2,1,2	gaacatctgtggtatggcgggtaagttttattaagacact	tttaggatgaaagcattccgctggcagcttaagcaatt	ttgctgaatcgagacttgagtgtgcaagagcaacc	accctagtgttcggtgaatatccaaggtacgcttgtag	tccttcttcaacaatcagatagatgtcagacgcatggctt
		299	MsrSA Erythromycin, oleandomycin azithromycin,	S. aureus	AB016613	2005-3471	5	1,2,2,2,1	ccaccaaatagagggaattgattgttctcctaaagtgca	tcgtcaacttgcttatgaagacatgcgtgacgttt	aacttgggtttaaatgaagcacttgagcgttcttgtaatg	tgctgacacaatttgggatatacaagaagatggcaca	tagcgctcgtaggtgcaaatggtgtaggtaagaca
		301	streptograminB lactonase (vgbB) Resistance to str	S. cohnii	AF015628	399-1286	5	1,1,1,1,1	agaggagtttaacttgtctattcccgattcaggtcca	ccggattctggtccttacggaataacggaaggtct	actttaggttcggataacgcactttggttcacggag	ccagtgggtatcactagtggtaacgatggtgcact	tattccaactccaaacgcacgtccacacgctataa
		302	Acetyltransferase (vat) Streptogramin	S. aureus	L07778	258-917	5	1,2,2,2,2	acaatgaccatggacctgatcccgaaaatattttaccg	tgggagaagtatatgccttccttaaaagatcttcccttga	tgtatggataggtagagatgtaaccattatgcctgggg	tgttgctccctattctattgtcggtggaaatccct	tgatggagttatcgaagaatggttagctttacaatggtgg
		303	Acetyltransferase (vatB) Virginamycin A-like resist	S. aureus	U19459	67-705	5	2,1,1,1,2	tggttgggaaaaagcaactccatctcttgaagatttacc	accatttaagggagatactgttgttggaaatgatgtgtgg	gtgtggattggtcagaatgttactgttatgccagga	ataggagatggagcaattgttgctgcgaattcagt	tgctgcgaattcagttgttacaaaagatgtaccacca
		304	StreptograminA acetyltransferase (vatC) Resistanc	S. cohnii	AF015628	1307-1945	5	2,1,1,1,2	tccattcaatcttttcggaaatggttgggagaagca	ggaagaccttccttataagggtaacacggaaattggg	gtttggattggacgagatgtgacaattatgcccgg	aggaaacggggctattattgcagcaaaatcggttg	tgatccttattcagttgttggcggtaatccttcacg
		305	StreptograminA acetyltransferase (satA) Resistanc	E. faecium	L12033	162-791	5	2,1,1,1,1	tgaatggagcaaatcatagaatggatggctcaacatatcc	atgttgtaattatgccaggagtaaaaatcggggatggtgc	aaaaatcggggatggtgcaatagtagctgctaattct	gttgtaaaagatatagcgccatacatgttagctggaggaa	agctgcttgatataaaatggtggaattggccaatagacat
		306	SatG putative streptogramin acetyltransferase; cor	E. faecium	AF139725	63-707	5	2,1,1,2,1	tgaacggtgccaaccacgtaatgaaaggtatttcg	attttaggtggcgattggcaacaatacactcctga	taggtgacggtgccattatcggagcaaatagtgtt	aagacgtcgctccatatacaattgtcggtggcaat	acctgacgcaaatgcaatctatcctaactcagcca
		307	ATP-binding protein (vga)	S. aureus	M90056	909-2477	5	1,1,1,1,2	accaacaaactttcttgatatggaagctatagaggcgttt	agatgtctcgggtacaattgaaggacgggtattgt	aggagacaagatggcaattatcggatctaatggtacagg	gtgtcttaagtggtggagagcgagttaaagtagcac	ttgtgcatgggaatcctggtatttcattatcgcca
		308	Pristinamycin resistance protein VgaB pIP1633	S. aureus	U82085	629-2287	5	1,1,1,2,1	acaaataaaggttgcttttgccaagctatttgttagcga	accaacaaactatttggatatcgatgctgttgaggca	agcccccttcaaaaacaatgggaacatctgaatct	tggatttacctaatagagagcaatttcatggtcgcaatgt	agatgcttcatttgtcattaaaggtggagaaaaggttgct
		309	Macrolide-efflux protein (mef)	S. pyogenes	U70055	314-1531	5	1,1,1,2,2	gctggttcggtgcttactattgttgcattctatatggag	agaacagcttacgaaatgtgcaggctatagtcagtct	tataagctatattgttagtccggcggttgcagcac	tgggaactaaatgctattattgccatcgatgtattgggt	tctattacggtagcaattgtacgtattcctaagctgggt
		310	Lincosaminide nucleotidyltransferase (linA)	S. aureus	J03947	645-1130	5	1,1,1,1,1	agaagttgattggatgccttcacgtatggaacttaaaca	atgatggttccattactcaagcaaacccagaaggt	acccagaaggtggcaattacatctttcaaaatgaatggt	aagtaactcattggttagatggaggctggggcgta	ggaggctggggcgtagatgtattaactggaaaaca
		312	mphBM Marcolide resistance pSR1	S. aureus	AF167161	5665-6564	5	1,1,1,1,1	ccaaaacttacaggtaaacccgcagccacaatagat	tagaaaacgacgaactatggcctcgacatgcgacc	caagcaaacgtcacaggtctcatagactggactga	cactccgacccatcaatggactttataggacacca	gcagtattcccaatgtttatcgctgagtttgctatgga
		396	ermT erythromycin resistance p121BS	Lactobacillus	NC_004957 (AF310974)	934-1668	2	2,1	tgagattggttcagggaaaggtcatttcacgcttg	acgcttgaattagctaaaaggtgtaattatgtaaccgcca			
		398	ermF Macrolide-lincosamide-streptogramin-resista	B. fragilis	M17124	1182-1982	5	1,2,1,1,2	acaatgttgttgctattgaaaacgacacagctttggt	ttctgatgcccgaaatgttcaagttgtcggttgtg	tcaaagtggtgtcaaatattccttatggcattacttccga	aatcctgatgtttgagagtcttggaaattttctgggagg	tcgattttcaggaaaagtcaggtcaggtcaatttcgg
		399	ermG CTnGERM1	B. ovatus	AJ557257	6631-7365	2	2,1	tgcagggaaaggtcattttactgctgaattggtaaaga	agatacaaacagatcactagcattgctgttaatggcagag			
		401	ermQ erythromycin resistance	C. perfringens	L22689	262-1035	4	1,2,1,1	ccaactgatatgtggctagttatggagaaaggttccg	tggagaaaggttccgcaaaaagatttatgggaatacct	agtgcactattttaatagagaagacttccatcccatgcct	acttccatcccatgcctagtgtagattgcgtctta	
		402	ermTR rRNA adenine N-6-methyltransferase	S. pyogenes	FM162351	5220-5951	5	2,1,1,1,1	tgggtcaggaaaaggacattttaccaaggaacttgt	aggaacttgtggaaatgagtcaacgggtgaatgct	gcaaaatacccaacgagctttaggtttgctgttaatgg	gtgccacgagcatattttcaccctaagcctaatgt	caggtgctgaagcatgcgaatgttactgatcttgat
		403	mel Efflux gene Tn2009	S. pneumoniae	AF376746	2709-4172	5	1,1,1,1,1	acccagtaacttccttgacataccaagtttagaggct	tcacccacgataaacgattactcgaaaatgtagcagatg	ctgcagaatacgaacaatttattgcggaacgtgcc	gaagctccggaaggcattcgcagaattcgtttcag	ggagctccataatccataccctatagtcggtgcag
	Oxazolidinone	394	rRNA methylase cfr PhLOPSA resistance pSCFS6	S. warneri	AM408573	10028-11077	5	2,2,1,1,1	tgcggatgtaattttgggtgtaaattttgtgctacaggc	tgaagctctagccaaccgtcaagtatttgatgctc	gcctggtgtaaatgattctcttgagcatgcaaacg	gtgcacctgagatgtatggagaagcaaacgaaggg	gccttttacaaagttttgaagtctgctggtatccatgtca
	Chlor-amphenicol	387	Chloramphenicol acetyltransferase orf10 pRE25	E. faecalis	X92945	8935-9582	2	1,2	acctgaaaacaccataccgatttcaatgattccttgga	tgctctgcaacttcatcattctgtatgtgatggttacc			
		389	Chloramphenicol acetyltransferase CAT pKH19	S. aureus	EU170347	1735-2385	3	1,1,2	actggttacaatagcgacggagagttaggttattggg	accgctatctttacaggtacatcattctgtttgtgatgg	tctattcaggaattgtcagataggcctaatgactggct		
		390	Chloramphenicol acetyltransferase CAT pSCS7	S. aureus	M58516	90-719	1	1	gccgatacctgaaaacacagttcctatctcgatgattcc				
		391	Chloramphenicol acetyltransferase catD Tn4453a	C. difficile	AF226276	3584-4207	5	1,1,2,1,2	ccgccattcagagtttaggacggcaatcaatcaag	gtgatacgcaacggtatggaaacaatcatagaatggaagg	tgataccgtggtcaaccttcgatggctttaatctg	acttcctttggcaattcaagttcatcacgcagtatgt	tttgcaagtgtaccttgtacatacagcatgaccgt
		392	Chloramphenicol acetyltransferase catQ	S. pneumoniae	AJ971089	27589-28248	5	1,2,1,1,1	cacaaggagttccgcaccagttttgatcaaaacgg	ttggacagagtatgacgagaacttcccacgtttct	gtgtccagcattccttgggtgaattttaccggattc	ggtgcatcatgcggtttgcgacggttaccatacca	atgcggtaaggtgcacttacagtatgactgcaaat
		393	Phenicol exporter fexA phenicol resistance	S. warneri	AM408573	14999-16426	5	2,2,1,2,2	tgcattcacgaattctcccgcaaataacgccgcag	ttatccgctcgacgtgatgctactgagccgataaa	acgctatgtcctactcagatgcgttccttgcagct	tgcctactacgcgttaccgacaattaaaccggcag	tgttcgtcccattactagtcgttgaggtgaatgga
	Trimethoprim	232	Dihydrofolate reductase Class I integron (dhfrXII) (	E. faecalis	AB196348	106-603	5	2,1,1,2,1	tctaccgaaccgtcacacattggtaatctcacgcc	gttgtttcaacgctgtcgcacgctatcgctttggc	cctcacgcccacggcatgtttctatctgaggtaca	aactcggaatcagtacgcatttatctcgttgctgc	acagaattcgagcttgtctcaaccgaaaccattca
		421	S1 DHFR dfrA Tn4003 pSK1	S. aureus	GU565967.1	16269-16754	5	1,1,1,2,1	gaccactgggaatacacttgtaatggcacggaaaa	gccaaatagacgtaacgtcgtactcactaaccaagc	aggacaaacgttatacgaagcaatgattgaccaggt	tctttccaccatacacattcgaaaactgggaagtcg	accgcatacattcttacatttagtgcgtagaaaagggaa
	Biocides	406	Transferable copper resistance tcrB	E. faecium	AY048044	4747-6879	5	2,1,1,1,1	tcgttgccttattgtcaggtatcgaaggtggatct	ctggcgctggtgtagaaggtcaagctaacggacat	gtatagctgttggtgccggaactcaagtagcattg	gggtataactttatcgcgatcccaatagctgcagg	cagtcggtgccgtcttgatgtcactttcgactgta
		407	Mercuric reductase merA	S. aureus	AB425427	5675-7318	5	1,2,1,1,1	agtcatcgaagccgatcaagtcctcgtggcaacag	tttgcaaacgtcgaataaccgaatatatgccgcgg	cgaaacgcaaaatcgatcttcgctttgttcccggc	cttcaccaatccatcgatcgccacagtcggcttga	acggatagctttgcgccttatttaacgatggctga
		408	Organomercurial lyase merB	S. aureus	AB425427	7400-8050	5	1,1,2,1,1	gcccgtcgaggaagttaagcaagtcctacagactc	ggaagcaactatatgcatggtgcgcccttgacaca	tggcttaaccaacaccctgagtcgagcgttttgcc	agccgtctctatggagtggttattccgtccgttgc	atttgggagcgcgttatgaggagtcaggacctact
		409	Cadmium-efflux ATPase Cadmium resistance cad.	S. aureus	J04551	1064-3247	5	2,1,1,1,1	tctatctcgagaaattaggagccattaagacagtcgca	gcaaatgcaatcggtacacatgtaggcgtttctga	cggagtatgataatgtagctatgattggcgatggcg	ggcggtgctggaacggatactgcaattgaaacagc	ccctttggatagcgattctttccgatatgggagct
	Metals	410	Quaternary ammonium qacA Antiseptic resistance	S. haemolyticus	AJ400722	6466-8010	5	1,2,1,2,1	gcggcgcgatttggaccgaaaatagtgttaccttc	tggagctggtatggcttcactagcagttgcatctg	atctgtagtgggcgctgtcgaagtagctaaagcta	ttaatgatgcttttgtagcaactgctttagtaggtgggat	caattgcaggcatgattggactggtatggagtatcaaag
		412	Multidrug resistance efflux protein Smr (formerly q	S. aureus	AF051917	40055-40378	3	1,1,1	acacctaccactaaatataacttatgcaacttgggcgg	ggcgggactaggtttagtcttaacaaccgtagtct	agtcggcgtagtttcgttaaacattttcggaacatcg		
		413	Quaternary ammonium efflux pump qacG	S. haemolyticus	EU622633	1-204	3	1,1,1	acgcaacatgggcaggtctaggattagtatcaaca	aggcttcacaaagttatggccaacattaggtacact	atttttgcccttaaatataacttacgcaacatgggcagg		
		414	Quaternary ammonium efflux pump qacH	S. haemolyticus	EU622634	1-225	1	1	ccacttaatattacttacgcaagttgggcaggtttagga				
		415	Quaternary ammonium efflux pump qacJ	S. haemolyticus	EU622635	1-242	4	1,2,1,2	accaatcaatgtatcttatgcaacttgggcagggt	tgggcagggttaggactagttcttacaacaatagtttca	agcagaaggatttacaaaactttggccaacattaggc	tggccaacattaggcacacttatttcattcgggat	
Phage	*E. faecium* phage	315	phage protein HK97gp10family EfmE980_contig0(	E. faecium	NZ_ABQA00000000	15284-15679	5	1,1,2,1,2	agcatgttcttgcatatacgaaccacgtttaccgt	acgtttaccgtttgacttcgttccatcccagtaag	ttcttgagcgtactccccagcttttctaagtgctg	ctaagcgctttgtacacttcggttgcatcgacttc	tggacgttgtttgattgttccgaactcgataaagtgc
		316	Putative phage terminase large subunit EfmE980_	E. faecium	NZ_ABQA00000000	19424-21284	5	1,1,1,1,1	ccatccaatttccggctatctgaggcgcgtggttc	agcgacgctctcctgtatcacgatgtacccatccg	ctgcaaatcacgcatatgccgtttagcagcttgaa	gctggctggtatgcttccatcgattaatcgttccg	ggctttgatccataatcttgggtcatcagcctctt
		317	Prophage pi3 protein33 EfmE980_contig00019	E. faecium	NZ_ABQA00000000	26937-27254	5	1,1,2,2,1	tcacgcatctgatcgaaccctttttgaatttcgttgta	caatgtttgctgctgctttccaatgcctgctacttc	tgctcctgtttcagccatttctaacttaactaattcagct	tctacaaatttgaaacgatcttgatggagcttctgactcc	cctgtttgctgatccagctcacgcatctgatcgaa
		318	Phage protein putative EfmE980_contig00096	E. faecium	NZ_ABQA00000000	1584-2264	5	1,1,2,1,1	ataaggtagctgaggcttatcctgaatcggcacaa	ccagtggtgcctaagtttgtggcggagtggtttgaa	aaaccctcatccgcatgaaagacggttacgaggtc	cgatttagtcattcgtaaaggtgagcatgaggcga	atgagcatggtcaaggaaggtacggcgcttatgaa
		319	Phage terminase large subunit EfmE980_contig0(	E. faecium	NZ_ABQA00000000	6835-8079	5	1,1,2,2,1	aggaatatgacgatgtactagactccgttcgatacgc	aagctatttggcgttaaggtcgtacaagcttatacagg	ttggtgcaattcgtggtatgacagcatatggcgct	tcgtagttataggtgaaacggatgacggaacagct	taaagcacggttatctggagtcgaagaagtcgcca
		320	Phage protein HK97gp10family EfmE980_contig0(	E. faecium	NZ_ABQA00000000	13120-13458	5	1,2,1,1,1	ggtgcgccagtggatacaggatacttacgaagatc	tggtttaactggtattgtaggaccgacagcagact	gaccgacagcagactatgctccttatgtagaatatgga	tatggaactcgctttatgtcggcccagccttatgt	acaacttgagttcaagggaatcgatcagctgatgc
		321	Integrase EfmE1039_contig00025	E. faecium	NZ_ACOSO1000000	17400-17986,1	5	2,1,1,2,2	tgagtgcagcgtaagcggagtgccacttgtagata	atattgatcgggatctgtgggtctacctgcaagaa	gcacgattgggtaattgcacttcacgttgcgaata	aactcagctattatgccgtgtagttttctgcgagg	ttggaaccttcaaatagctagtacgtttaacatgctgct
		322	Phage anti-repressor protein EfmE1039_contig00(	E. faecium	NZ_ACOSO1000000	20736-21491	5	1,1,1,1,1	ggaggacgcccacgtattgatcacgcaatgaaact	gcgtatcagaaattatagcgctaaatactctcgattggcg	gcaaactcattgataaatcaagttgctcgcgtgca	gttgaacgacgagctggagctaatctttctcaacg	caaagcgtgacaaacttaatcaggtagatgtgatagctga
		323	Excisionase putative EfmE1039_contig00025	E. faecium	NZ_ACOSO1000000	21504-21803	4	2,1,1,2	aacactttcagagttgatagaagctgctgagaaggc	agaaggaattgttcgtccaggacattcaaccacat	agacgactggtaccgacaaagtttaatcttggagaagt	tggagaagttccacggcatgtcaaaaactactttagt	
		324	Phage protein EfmE1039_contig00043	E. faecium	NZ_ACOSO1000000	3042-3752	5	1,1,1,2,2	atcccgttactggtctcgagaaaaggttgcaacca	ccaaagaagtttctaagtggtcctcgagtgattctgg	gagaaatagcggaaaagtatggtgtatccatcagcaca	tgaacactcaagttgatctagctaaagcacaattgaagca	tgatgacattccgttagttgaaagcgaggctggtt
		325	Phage terminase EfmE1039 large subunit pbsx fa	E. faecium	NZ_ACOSO1000000	3718-5043	5	1,1,2,1,2	aattacgattgattcggcagaaggcgcattaagga	gacatgattgatcacgttcaaagcttacttgcacaagg	tgcgataggcgatattatctcattgtggtatgaagaagct	ctctatcgtggcgaagtcattggattagggactca	tttctcaatcgatagcgggcaccaagtgtcagcta
		326	Phage minor structural protein GP20 EfmstrainE1(	E. faecium	NZ_ACOSO1000000	7845-8420	5	1,1,1,1,2	ggcttaactgatgaacagattggatcaattatggctttgc	gctgctaaagccttgctagacagcgaatcattgga	aaggattagatgaacaactggccgcactcaaagag	caggttccacctaatcccgatggtaagaaggctac	tgactgtgaacgagttgaatagtcgggtgtctacc
		327	Phage terminase small subunit EfmstrainE1039	E. faecium	NZ_ACOSO1000000	4229-4684	5	2,1,1,1,1	ttcaaactaagaggcatttaacaatggaagaagctttggc	agctttggctattactgcttctattgcaaaaggagaacc	acgaaatcatagaacgtgaagtgagtgaatattcagcagg	agcaggtttcaaagaacgtaaccaagcacttgagc	tcacaggcaatgcttatcagtcagcgctgaaagct
		328	Phage terminase large subunit EfmE1039_contig(	E. faecium	NZ_ACOSO1000000	4827-5975	5	1,1,2,2,1	accgcgtggtaactacatgatatttcgtggagcgc	aagtaacgactatcaccaactccctattgcgtgga	tggtaaagctattggatctggtattgtaccgttcgaca	tcgccaagggcttgactacggttacgcaacagatc	atcactaccaggatatcatcgcagaggttgaacct
		329	Phageprotein HK97gp10family EfmstrainE1039	E. faecium	NZ_ACOSO1000000	12702-13073	5	1,1,1,1,1	gtgcacccgacaggcttcactagacgatcaattcg	cacaagttggaccgcagagtgattacagtccgtac	ggtgtggatgagctgcttaaacacttggaagaagc	atgagcgctcaacctttcgttggtcccgcatttaa	gaagctgtcacgcttagagatgtgcaaatggtagt
		342	Prophage Lp2 protein 33 EfmE1039_contig00056:	E. faecium	NZ_ACOSO1000000	7817-8080	5	2,1,1,2,1	tgccaaatggattacctggtattgaaacattagaaggca	ggcgttcatggtgaattttatccgtgcaaaccaga	agttactgtcgagattgtgcaacttaaaatgctatctgca	tgctatctgcacgttcatatcgcaaatgcaaagaattc	gcaaagaattcgttggtgaagcatgggttgaccat
		333	Phage prohead protease HK97family EfmE1679_c	E. faecium	NZ_ABSC00000000	1943-2449	5	2,1,2,2,1	tcttgctaccgttgtgtctggcaaatcgacttcga	accatgatcgtcttctgacaatcgaagggttccgc	tcggagttactaatcgttctagcaaaagcgcctgg	tgttgggctattgaatacagcagcatatccactaatgg	tcagaacgcgttgaaaggttggtgatgtcaatcgt

	334	Putative phage terminase large subunit EfmE1679	E. faecium	NZ_ABSC00000000	3672-5339	5	1,1,2,2,1	tcagaagcgcatgcataacaatcgactgacaaagc	ctccatacattgcatagcctaatgaaccgacagca	ttggacatacgcttcacgagtaacacggacaatttg	tgttttaaatgcggactctaaagcggaaagatcgact	tcacgatatacgcgcattccaacagacgatgaacc
	336	Phage anti-repressor protein EfmU0317_contig00	E. faecium	NZ_ABSW01000000	51174-51950	5	1,2,2,1,1	gggttattcaaacactaaagatgctttgtcgcggc	tcgcgaatcacgacctcaggtcaatcaagagagat	tttaaacgttgggtaacaagtgaagtgttgccagc	tcagattgcagcagattatgggatgtctccacaac	tcatacaactgagatcccgaaagccgatggtggca
	337	Phage endonuclease EfmU0317_contig00056	E. faecium	NZ_ABSW01000000	62625-62843	4	1,1,2,2	atttatgaaagagaaggtggtcattgtcagcggtgt	ggtcattgtcagcggtgtggtcagttcatatttgga	tttggaaagagagcacacgtccatcacattgtacc	tccatcacattgtaccaatcaaagacaacgaactgct	
	338	Phage prohead protease EfmU0317_contig00056	E. faecium	NZ_ABSW01000000	66354-66923	5	1,1,1,1,1	agcgaagtgattgttcctggtgcattctcaaaggc	ccaatgtgctaggacgtactaaaagcggaacccttcg	gaacgtggtttgaaattcgaagttgatttaccggacac	gtgggactacaattctgaaccaatgcttcgaacaatcca	cggctacgctgctgtattcaacagtcaaacacttt
	339	Phagesite-specific recombinase EfmU0317_ctg00	E. faecium	NZ_ABSW01000000	6735-8087	5	1,1,1,1,1	ctctaagactatctccatgtttggcttgttgttcagttga	cttgctttaatagtttgaccagctactgatccctgacag	tagcctaacggagttttcccgcttactacttcacc	gcgttctgaatcaatttgggcttccaattcagcaa	acttgtgcaatgaaagttctgccatacgctgtagt
	340	Phage terminase small subunit EfmU0317_contig	E. faecium	NZ_ABSW01000000	21549-22382	5	1,2,1,2,1	agtaacgttactaatcaaggtggcgctcctattggt	aacaaaggaaatagccgagcttctccgccagtggg	acaatttcgtatgatgaagagaatccaacaagctgaagct	tggactaaatgatgaagaagtcgaacgattgcagca	aattgaagattcattgactcggattagcaaccagttagct
	341	Prophage pi2 protein 43 EfmU0317_contig00121s	E. faecium	NZ_ABSW01000000	33975-34715	4	1,2,1,2	agtgggaagatcgaatcactgaatggttagatatgtcgt	tccctttttgaatttacagtgagcattcgaccatttaagga	cttcaaaaccgcttattaaattgagtggttctggagatgc	tctgtttcagctagtcgagtaattgaattgagagagcga	
***E. faecalis* phage**	343	Phage site-specific recombinase EF2955 phage in	E. faecalis V583	NC_004668	2828409-28295	5	1,1,1,1,1	aggtgactgcaaacttgacgaaataaccgttatacagt	aggttgctagcctttacaggttgtcgtaagagtga	agcgtatatataggcaagaccgtagcacaagacga	acagcttctagcacacgttctatcagcttagacaatga	gctaaatggcgtacaatccaacgtagtgactatttcca
	344	Site-specific recombinase EF2855 phage integras	E. faecalis V583	NC_004668	2735627-27368	5	1,1,1,2,2	agttagggaatccacattcatagctactgaacgacgt	cattcgggaaaatgcgacttgagcgtttaacagtc	actggtatacgtgttggtgaagctttagcattgaattgg	tcccgaagtcaagacatttaccaacgttctaaaagattgg	tctattggttgtcatggttttcggcatacccacgc
	348	Site-specific recombinase EF2043 phage integras	E. faecalis V583	NC_004668	1961337-19624	5	1,1,1,1,1	agcaaacattaagtacgtttcaactcggttaggacaca	agtctggataccttacgcccaagacaattggagtg	gtggcataaaattgtaatgaggatgattaacgctgcgg	atcaaatcatccctagcaacacgctaacaggcttt	cataaccgtacacggcctaagacatacccatgcaa
	351	Phage integrase EF0303 (pp1)	E. faecalis V583	NC_004668	289354-290533	5	1,2,1,1,1	cgagggtcggtgtatctttcgcgatattggtatgt	tcctcgggtgtgactacgtgttgcatgatcgtaag	accaaaacgcatcccagtgtgaaattgtagttcga	tataggcgagagtaaatgcaccacggataatcgca	aatcaatgccatagctatatcccgagagcgtggaa
	353	Site-specific recombinase EF1417 phage integras	E. faecalis V583	NC_004668	1398097-13993	5	2,2,2,2,1	tgccagtaccattgctacgtccatttgaaacgaag	tccatttgaaacgaagtattcccaacgttcttgacca	tgctcttttctatacaaacgctgcttgtcaaatgtgaga	ttacttcgcctttccgcaagcctaagtcgaaaatg	cggatagcatctcgatagacaattcgtgatccttcct
**CRISPR-cas**	474	csn1 family CRISPR-associated protein	E. faecalis OG1RF	CP002621.1	416527-42054(	5	2,2,2,1,1	aaccacggttcacgaaggatggcgaaattctttgg	ttgatagtcctgtggttgcttatacagtgttattcacgc	ttcctgcggcctcgtgtattaatgaaattgcctaa	agagattgcggcatcctttatccaacttatgcagt	tacgggactgtatgaaacacgcagaaaggttgtcg
	475	Cas1 CRISPR Associated Protein	E. faecalis OG1RF	CP002621.1	420541-421407	5	2,1,1,2,1	tgcgatgataagcgtttgccaattggtaaaattttgcc	gtagcttgcaattgacacgccagttggcttggacg	gggctggcgaacggtagtggttaacaagcattcaa	ttacacttagcgcaacgagactatggtcaaaaggc	ctatgcgcgctgaattacgtctgtttgatccagcc
	476	Cas2 CRISPR Associated Protein	E. faecalis OG1RF	CP002621.1	421404-421733	5	2,2,2,2,1	ttgttgaatgatactgctaataaagcaatgttggctcgc	tgcgtctattattaatgtttgatatgccaacggacacagc	tgttggctcgcttaaagcagaataatccgcaaagag	tggagaacaagacaatcgagttgctaattcagatgaacg	cacagccagtgaccggaaagcgtatcggaagttcc
	477	csn2 family CRISPR-associated protein	E. faecalis OG1RF	CP002621.1	421734-422393	5	1,2,2,1,1	attttaggctatgacgttaattcggctgccacgct	tgccacgctgaaattaatctatggggatttagaagca	tttggaagaagatggcatcactgtgcaggaacttt	gcgtgtacgtatttgacagaggatgaagtgcaaca	acgattaaagacgttcaaacctttgcccatctagtga
	478	Conserved CRISPR sequence Repeat pHTbeta	E. faecium	AB183714.1	43550-43717	1	2	gttttggagctatataaaacgacatggttctcaaat				
	479	Conserved CRISPR sequence Repeat	E. faecalis OG1RF	U41084.1	1355-1786	1	2	gttttagagtcatgttgtttagaatggtaccaaaac				

*SSTE; Site specific transposable element
