# Supplementary material for: Investigating the mobilome in clinically important lineages of Enterococcus faecium and Enterococcus faecalis
Source: BMC Genomics. 2015 Apr 10;16:282. doi: 10.1186/s12864-015-1407-6 (PMC4438569; doi:10.1186/s12864-015-1407-6)
Supplement: Additional file 5: Figure S3. — Summarized gene profile of four enterococcal STs. Number of positive E. faecium ST17 (n = 10) and ST78 (n = 10) and E. faecalis ST6 (n = 10) and ST40 (n = 10) for selected targets within five groups; A: plasmid replicons and plasmid addiction systems; B: transposable elements; C: resistance determinants; D: phage; and E: CRISPR-Cas, determined by DNA microarray. Detailed information of each microarray target is found in Additional file 4: Table S2. Note that the presence of pIP501 repR, pCF10 prgW, pLG1 rep, and aac(6’)-aph(2”)-Ia are determined by PCR. The presence of ω-ε-ζ, vanA, vanB, and vanG genes are also verified by PCR. Chi square (χ2) calculations are done without corrections, and with 1 degree of freedom using the following online calculator: http://www.graphpad.com/quickcalcs/contingency1.cfm (last accessed December 10th, 2014). Where statistically significant differences (p < 0.05) between number of positive strains in each species (E. faecium/E. faecalis) are indicated by light grey (of the species with most positive strains) and between STs (ST17/78 or ST6/40) by dark grey (of the ST with most positive strains). White boxes indicate no significant differences. Verifying PCRs are not included in the calculations. [file 12864_2015_1407_MOESM5_ESM.pdf]

FIGURE S3

A

Rep family/ *rep* class*	Target no.	Gene/ORF	Plasmid	*E. faecium* ST17	*E. faecium* ST78	*E. faecalis* ST6	*E. faecalis* ST40
**Rolling-circle replicating plasmids**							
Rep_trans							
**14**	**19**	*rep*	pEFNP1	7	7	1	5
	**21**	ORF	pKQ10	5	8	0	2
	**371**	*rep*	pRI1	3	2	0	0
**7**	**360**	*rep* (CDS11)	pRE25	0	0	7	4
	**374**	*repM*	pRUM	2	0	1	0
	**431**	*repD*	Plasmid	0	0	4	3
**Theta replicating plasmids**							
Rep_3							
**4**	**361**	*repB*	pMBB1	0	0	0	0
**5**	**362**	*rep*	pN315	0	0	0	2
	**363**	*rep*	pN315	0	0	0	0
**6**	**7**	*rep*	pS86	0	0	2	0
11	***30***	*repA*	*pB82*	*1*	*4*	*0*	*2*
	13	*repA*	*pEF1071*	*0*	*0*	*0*	*0*
	28	*rep*	*pEFR*	*1*	*4*	*0*	*0*
18	***31***	*repA*	*p200B*	*4*	*4*	*0*	*0*
	9	*repA*	*pEF418*	*2*	*1*	*0*	*0*
	20	*rep*	*pCIZ2*	*1*	*6*	*0*	*0*
Inc18							
**1**	**17**	*repE*	pAMβ1	1	1	0	0
	**PCR**	*repR*	pIP501	3	1	3	4
**2**	**3**	*rep* (CDS1)	pRE25	7	7	9	5
	4	*repS*	*pTEF3*	*0*	*0*	*3*	*0*
	27	*rep*	*pHTβ*	*1*	*0*	*0*	*4*
RepA_N							
8	***6***	*rep*	*pAM373*	*0*	*0*	*0*	*0*
	10	*repA*	*pEJ97-1*	*0*	*0*	*0*	*1*
9	***365***	*prgW*	*pCF10*	*0*	*0*	*0*	*0*
	PCR	*prgW*	*pCF10*	*0*	*0*	*10*	*5*
	14	*repA-1*	*pTEF1*	*3*	*1*	*1*	*6*
	16	*repA*	*pTEF2*	*0*	*0*	*9*	*4*
17	***5***	*repA*	*pRUM*	*9*	*9*	*0*	*0*
19	***375***	*repA*	*pUB101*	*0*	*0*	*0*	*0*
20	***PCR***	*rep*	*pLG1*	*6*	*8*	*0*	*0*
**Plasmid addiction systems**	***457***	*axe* (antitoxin)		*8*	*7*	*0*	*0*
	458	*txe* (toxin)		*9*	*8*	*0*	*0*
	460	*ζ* (toxin)		*1*	*7*	*0*	*10*
	461	*ε* (antitoxin)		*3*	*8*	*7*	*10*
	462	*ζ* (toxin)		*2*	*3*	*1*	*10*
	463	*ω* (repressor)		*8*	*8*	*8*	*10*
	PCR	*ω-ε-ζ*		*6*	*8*	*7*	*10*
	464	*mazF* (toxin)		*0*	*0*	*10*	*10*
	465	*mazE* (SpoVT/AbrB-like protein)		*0*	*0*	*10*	*10*
	466	*relE* (toxin)		*1*	*2*	*0*	*4*
	467	*relB* (bifunctional antitoxin)		*1*	*0*	*1*	*0*
	468	*par* (toxin RNA I)		*0*	*0*	*4*	*6*
	469	*par* (antidote RNA II)		*0*	*0*	*3*	*6*

B

Transposable element or IS family	Target no.	Gene/ORF	Associated transposon	*E. faecium*		*E. faecalis*	
				ST17	ST78	ST6	ST40
Tn*916/1545*	199	Integrase (ORF2)	Tn*916*	2	0	10	10
	213	Excisionase (xis-Tn)	Tn*916*	2	0	10	10
	75	Orf16 *tra* gene	Tn*916*	5	2	10	10
Tn*1549*/*5382*	452	Integrase	Tn*1549*	3	2	2	1
	454	Excisionase	Tn*1549*	3	2	2	0
	82	Conjugative transfer protein	Tn*1549*	4	2	1	0
	39	Relaxase	Tn*1549*	3	2	1	5
Tn*1546*	146	Transposase	Tn*1546*	2	3	3	1
	216	Resolvase	Tn*1546*	2	3	3	1
Tn*917*	145	Transposase	Tn*917*	0	0	2	0
	229	Resolvase	Tn*917*	0	0	1	0
IS*256*	96	Transposase IS*256* family		6	7	10	9
	15	Transposase IS*256*		8	9	10	9
	154	Transposase IS*256*		8	9	10	8
	102	Transposase IS*1542*	Tn*1546*	4	0	0	0
	98	Transposase IS*16*		10	10	1	2
	134	Transposase IS*Ef1*		10	10	3	4
	157	Transposase IS*1310*		0	0	0	0
IS*110*	104	Transposase IS*Enfa110*	Tn*5382*	0	0	0	0
	165	Transposase IS*110* family		0	1	10	1
	167	Transposase IS*110* family		10	10	0	0
IS*3*	109	Transposase IS*Enfa3*		10	10	0	0
	110	Transposase IS*1485*		10	10	10	10
	162	Transposase IS*3*-like		0	0	0	0
	143	Transposase IS*981*		7	10	7	5
IS*6*	111	Transposase IS*6* family		0	0	0	1
	112	Transposase IS*1216*		10	10	10	6
	156	Transposase IS*Enta*1		9	9	10	6
IS*L3*	118	Transposase IS*L3* family		0	0	0	1
	120	Transposase IS*L3*-like		0	0	0	0
	117	Transposase IS*1251*	Tn*1546*	6	3	1	0
	119	Transposase IS*Efa11*		10	10	0	0
	123	Transposase IS*1476*		8	8	0	1
	176	Transposase IS*1167*		10	10	10	6
IS*982*	129	Transposon IS*Efm1*		10	10	4	0
IS*1182*	138	Transposase IS*1182*		5	2	6	0
IS*200*/IS*605*	170	Transposase IS*200* family		0	0	6	10
	142	Transposase IS*Enfa200*	Tn*5382*	0	0	0	0
	140	Transposase IS*Efa4*		7	9	0	0
	166	Transposase IS*605*		6	10	0	0
IS*1380*	126	Transposase IS*Ecp1*-like		0	0	0	2
	137	Transposase IS*1678*	Tn*1546*	0	8	2	0
IS*30*	130	Transposase IS*1252*		0	1	1	2
	133	Transposase IS*6770*		8	10	6	10
	136	Transposase IS*1062*		1	0	2	1
IS*Enta1*	156	Transposase IS*Enta1*		9	9	10	5
	168	Transposase IS*4*		5	1	0	0
IS*21*	160	Transposase IS*21 family*		0	0	0	0
	417	Transposase IS*21-558*	Tn*558*	0	0	0	0

C

Antibiotic	Target no.	Gene/ORF	*E. faecium* ST17	*E. faecium* ST78	*E. faecalis* ST6	*E. faecalis* ST40
Glycopeptides	**237**	*vanA* Tn*1546*	3	3	3	1
	**PCR**	*vanA*	4	2	3	1
	**238**	$vanB_2$	3	2	1	0
	**PCR**	*vanB*	3	2	1	0
	**280**	*vanE*	0	0	0	0
	**PCR**	*vanE*	1	2	0	0
	**281**	*vanG*	0	0	0	1
	**284**	$vanG_2$	0	0	0	0
	**PCR**	*vanG*	0	0	0	0
	**313**	*vanL*	0	0	0	2
Aminoglycosides	**251**	*aph(2')-Ib*	0	1	0	1
	**253**	*aph(2')-Ic*	0	0	0	0
	**252**	*aph(2'')-Id*	0	0	0	0
	**PCR**	*aac(6')-Ie-aph(2'')-Ia*	5	6	7	2
	**381**	*aph(3)-IIIa*	5	8	7	4
	**254**[1]	*aac(6')-Ii*	10	10	0	0
	**383**	*ant(6)-Ia*	6	7	7	5
	**256**	*aadA*	0	0	0	0
	**258**	*aad9*	0	0	0	0
	**430**	*str*	0	0	1	5
	**382**	*aadD*	0	0	0	0
	**263**	*aadA2*	0	0	0	0
	**384**	*spc*	0	0	0	1
	**247**	*sat4*	5	6	7	4
β-lactams	**473**	*pbp5*	7	10	0	1
	**420**	*blaZ* Tn*4002*	0	0	0	0
Tetra-cyclines	**243**	*tetM*	7	3	10	10
	**242**	*tetL*	3	3	1	3
	**262**	*tetK*	1	0	1	3
	**385**	*tetO*	0	0	0	0
	**386**	*tetS*	1	0	1	8
Macrolides, lincosamides, streptogtamins (MLS)	**260**	*ermA* Tn*554*	0	0	0	0
	**298**	*erm2*	8	9	10	3
	**398**	*ErmF* Tn*4351* (IS*4351*)	0	0	0	0
	**399**	*ermG* CTnGERM1	0	0	0	0
	**401**	*ermQ*	0	0	0	0
	**396**	*ermT*	1	0	0	2
	**402**	*ermTR*	0	0	0	0
	**309**	*Mef*	0	0	1	6
	**233**	*msrC*	0	0	10	10
	**403**	*mel* Tn*2009*	0	0	0	0
	**312**	*mphBM*	0	0	0	0
	**299**	*msrSA*	0	0	0	0
	**240**	*vgB*	0	0	0	2
	**301**	*vgbB*	0	0	0	0
	**302**	*Vat*	0	0	0	0
	**303**	*vatB*	0	0	0	1
	**304**	*vatC*	0	0	0	1
	**305**	*satA*	0	0	0	1
	**306**	*satG*	1	1	1	3
	**307**	*Vga*	0	0	0	0
	**308**	*vgaB*	0	0	0	0
	**310**	*linA*	0	0	0	0
	**234**	*linB*	0	4	1	2
Oxa-zolidinone	**394**	*cfr*	0	0	0	1

[1] This target is species specific for *E. faecium*

**Chloram-phenicol**	**387**	*orf10* pRE25	0	0	6	3
	**389**	*cat* pKH19	1	0	0	0
	**390**	*cat* pSCS7	3	0	2	3
	**391**	*catD* Tn*4453a*	0	0	0	0
	**392**	*catQ*	0	0	0	1
	**393**	*fexA*	0	0	0	0
**Trimetho-prim**	**232**	*dhfrXII*	0	0	0	0
	**421**	*dfrA*	0	0	0	0
**Biocides**	**406**	*tcrB*	0	1	1	2
	**407**	*merA*	0	0	0	0
	**408**	*merB*	0	0	0	0
	**409**	*cadA pI258*	1	0	1	6
	**410**	*qacA*	0	0	0	2
	**412**	*smr*	0	0	1	6
	**413**	*qacG*	0	0	0	0
	**414**	*qacH*	0	0	1	0
	**415**	*qacJ*	0	0	0	0

D

**Phage sequences**	**Target no**	**Gene/ORF**	***E. faecium***		***E. faecalis***	
			**ST17**	**ST78**	**ST6**	**ST40**
***E. faecium* E980**	**315**	phage protein HK97gp10 family EfmE980_contig00019	1	0	0	7
	**316**	Putative phage terminase large subunit EfmE980_contig00019	0	0	0	7
	**317**	Prophage pi3 protein33 EfmE980_contig00019	8	4	0	2
***E. faecium* E980**	**318**	Phage protein putative EfmE980_contig00096	0	1	0	0
	**319**	Phage terminase large subunit EfmE980_contig00096	1	0	0	0
	**320**	Phage protein HK97gp10 family EfmE980_contig00096	2	0	0	0
***E. faecium* E1039**	**321**	Integrase EfmE1039_contig00025	1	1	0	0
	**322**	Phage anti-repressor protein EfmE1039_contig00025	0	0	1	0
	**323**	Excisionase putative EfmE1039_contig00025	0	0	0	0
***E. faecium* E1039**	**324**	Phage protein EfmE1039_contig00043	0	0	0	0
	**325**	Phage terminase EfmE1039 large subunit pbsx family	0	0	0	0
	**326**	Phage minor structural protein GP20 EfmstrainE1039	0	0	0	0
***E. faecium* E1039**	**327**	Phage terminase small subunit EfmstrainE1039	2	0	0	0
	**328**	Phage terminase large subunit EfmE1039_contig00056 pbsx family	1	0	0	0
	**342**	Prophage Lp2 protein 33 EfmE1039_contig00056strain	2	0	0	0
	**329**	Phageprotein HK97gp10 family EfmstrainE1039	1	0	0	0
***E. faecium* E1679**	**333**	Phage prohead protease HK97family EfmE1679_contig00158	0	0	0	0
	**334**	Putative phage terminase large subunit EfmE1679_contig00158	0	0	0	0
***E. faecium* U0317**	**336**	Phage anti-repressor protein EfmU0317_contig00056	9	9	0	0
	**337**	Phage endonuclease EfmU0317_contig00056	3	7	0	2
	**338**	Phage prohead protease EfmU0317_contig00056strain HK97family	3	7	0	0
***E. faecium* U0317**	**339**	Phagesite-specific recombinase EfmU0317_ctg00121	0	8	0	0
	**340**	Phage terminase small subunit EfmU0317_contig00121strain	2	6	0	3
	**341**	Prophage pi2 protein 43 EfmU0317_contig00121strain	3	8	0	0
***E. faecalis* V583**	**343**	Phage site-specific recombinase phage integrase family	0	0	2	0
	**344**	Site-specific recombinase phage integrase family	0	0	9	8
	**345**	Site-specific recombinase phage	0	0	0	2
	**346**	Site-specific recombinase phage integrase family	0	0	9	1
	**347**	Site-specific recombinase phage integrase family	0	0	0	0
	**348**	Site-specific recombinase phage integrase family	0	0	9	9
	**350**	Site-specific recombinase phage integrase family	0	0	10	10
	**351**	Phage integrase	1	0	4	8
	**352**	Site-specific recombinase phage integrase family	3	2	10	8
	**353**	Site-specific recombinase phage integrase family	0	0	6	0
	**354**	Site-specific recombinase phage integrase family	0	0	9	0

E

	Target no	Gene/ORF	*E. faecium* ST17	*E. faecium* ST78	*E. faecalis* ST6	*E. faecalis* ST40
CRISPR/ Cas	**474**	Cas_Csn1 CRISPR Associated Protein	0	0	0	9
	**475**	Cas1 CRISPR Associated Protein	0	0	0	9
	**476**	Cas2 CRISPR Associated Protein	0	0	0	9
	**477**	Cas_csn2 CRISPR Associated Protein	0	0	0	9
	**478**	Conserved CRISPR sequence Repeat pHTbeta	0	0	0	0
	**479**	Conserved CRISPR sequence Repeat	0	0	10	10
